# The extent, nature, and pathogenic consequences of helminth polyparasitism in humans: A meta-analysis

**DOI:** 10.1371/journal.pntd.0007455

**Published:** 2019-06-18

**Authors:** Rose E. Donohue, Zoë K. Cross, Edwin Michael

**Affiliations:** 1 Department of Biological Sciences, University of Notre Dame, Notre Dame, Indiana, United States of America; 2 University of Utah, Salt Lake City, Utah, United States of America; University of Washington, UNITED STATES

## Abstract

**Background:**

Individual helminth infections are ubiquitous in the tropics; geographical overlaps in endemicity and epidemiological reports suggest areas endemic for multiple helminthiases are also burdened with high prevalences of intestinal protozoan infections, malaria, tuberculosis (TB), and human immunodeficiency virus (HIV). Despite this, pathogens tend to be studied in isolation, and there remains a need for a better understanding of the community ecology and health consequences of helminth polyparasitism to inform the design of effective parasite control programs.

**Methodology:**

We performed meta-analyses to (i) evaluate the commonality of polyparasitism for helminth-helminth, helminth-intestinal protozoa, helminth-malaria, helminth-TB, and helminth-HIV co-infections, (ii) assess the potential for interspecies interactions among helminth-helminth and helminth-intestinal protozoan infections, and (iii) determine the presence and magnitude of association between specific parasite pairs. Additionally, we conducted a review of reported health consequences of multiply-infected individuals compared to singly- or not multiply-infected individuals.

**Principal findings:**

We found that helminth-helminth and helminth-intestinal protozoan multiple infections were significantly more common than single infections, while individuals with malaria, TB, and HIV were more likely to be singly-infected with these infections than co-infected with at least one helminth. Most observed species density distributions significantly differed from the expected distributions, suggesting the potential presence of interspecies interactions. All significant associations between parasite pairs were positive in direction, irrespective of the combination of pathogens. Polyparasitized individuals largely exhibited lower hemoglobin levels and higher anemia prevalence, while the differences in growth-related variables were mostly statistically insignificant.

**Conclusions:**

Our findings confirm that helminth polyparasitism and co-infection with major diseases is common in the tropics. A multitude of factors acting at various hierarchical levels, such as interspecies interactions at the within-host infra-parasite community level and environmental variables at the higher host community level, could explain the observed positive associations between pathogens; there remains a need to develop new frameworks which can consider these multilevel factors to better understand the processes structuring parasite communities to accomplish their control.

## Author summary

Helminth infections are a highly prevalent global health problem. These parasitic worm infections occur in areas also burdened with intestinal protozoan infections, malaria, tuberculosis, and human immunodeficiency virus. While these pathogens tend to be studied in isolation, there remains a need to better understand the nature, extent, and health consequences of helminth polyparasitism and co-infection with major diseases. Here, we reviewed the literature and performed meta-analyses to evaluate the commonality of helminth polyparasitism and co-infection, the potential for interspecies interactions between parasites, the association between parasite pairs, and the health consequences among multiply-infected individuals. We confirmed that polyparasitism and co-infection with major diseases are common in the global South and found that multiply-infected individuals experienced worse health consequences when compared to singly or not-multiply infected individuals. Our analysis suggested the potential presence of interspecies interactions and we identified the existence of positive associations between parasite pairs. These findings support the call for integrating deworming into malaria, TB, and HIV treatment protocols and suggest there remains a need to improve our understanding of the factors influencing co-transmission to achieve sustainable parasite control.

## Introduction

Helminth infections continue to be ubiquitous in the tropics with the 2016 Global Burden of Disease study indicating that currently 800 million individuals are likely to be infected worldwide with *A*. *lumbricoides*, 451 million with hookworm, 435 million with *T*. *trichiura*, and 190 million with schistosomiasis [1]. These figures suggest that worm infections may continue to induce significant morbidity on the world’s poorest populations; indeed, the latest 2016 disability-adjusted life year (DALY) estimates suggest that infection by these parasites could contribute to a loss of 6.6 million years lived with disability (YLD) presently, representing up to 6.5% of all the YLD due to communicable, maternal, neonatal, and nutritional diseases globally [1].

It has long been recognized that polyparasitism with helminths is a common feature of human infections in helminth-endemic regions. Areas endemic for multiple helminthiases have been shown to also harbor a higher burden of intestinal protozoan infections, malaria, tuberculosis (TB), and human immunodeficiency virus (HIV) [2]. Geographic patterns in endemicity, for example, have demonstrated that helminth co-infection with TB and HIV are pervasive in tropical geographies [2], and a recent meta-analysis indicated that soil-transmitted helminths and malaria may also be similarly co-endemic [3]. The transmission dynamics of these infections are influenced by polyparasitic infections; infection by one parasite species can alter host susceptibility to additional parasite species [4]. There is growing literature demonstrating that helminth infections can detrimentally reduce host resistance to the microbes causing TB, HIV, and malaria [5]. Additionally, helminth infections have been found to affect vaccine efficacy [6], which may also influence the occurrence of these major co-infections. For example, studies have found that helminths hinder the immune response to the oral cholera vaccine [7] and similarly that helminth-infected individuals have impaired immune responses to vaccines for tuberculosis and tetanus compared to non-helminth infected individuals [8–12].

These results, coupled with insights from studies of infectious disease transmission taking a community ecology perspective [13,14], suggest that helminth infections may continue to persist in the world’s poorest communities in spite of the enactment of large-scale national control programs. Indeed, increasing research has also demonstrated how interventions focused on one species alone in such a complex could result in unintended and potentially perverse health consequences resulting from the remaining infections [15–17]. These results indicate that gaining a better understanding of the extent and community ecology of helminth polyparasitism is a major need if effective control of these widespread and persistent infections is to be achieved [13,14,18,19]. In spite of these findings, parasites, including helminths, tend still to be studied in isolation, presumably because of the diagnostic challenges of undertaking multiple infection studies [20,21].

Despite the commonality and potential importance of helminth polyparasitism, the health consequences are not well-studied [20–23], likely due to the diagnostic challenges as well as the non-specific morbidity and chronic nature of helminth infections [20,24]. A 2008 review of existing studies on the health implications of soil-transmitted helminths, schistosomiasis, and malaria indicates that polyparasitism may have an additive and/or synergistic effect on nutrition and organ pathology [22]. An additional review of the literature related to all co-infections published in 2009 also found co-infections to be associated with larger negative health effects [23]. These studies indicate that by examining diseases individually, the true human health burden induced by the polyparasitic nature of helminth infections could be seriously underestimated [22,23].

The above indicates that quantifying the fundamental patterns of helminth polyparasitism, including the relative frequency of co-infection with various major pathogens and infection/morbidity differences between single-species and co-infection, will constitute a first step in assessing the potential impact that polyparasitism can play not only in shaping observed parasitic infection prevalences and pathology, but also for improving prospects for achieving effective parasite control in endemic communities [13].

Here, we report on a survey and analysis of the published data on helminth polyparasitism to address these questions. We performed a meta-analysis of the assembled data following PRISMA guidelines [25] to evaluate the frequency of helminth co-infections and the presence and magnitude of the observed interspecific associations between specific parasite pairs; whereas we conducted a review together with a vote-counting-based analysis of compiled studies to evaluate the morbidity outcomes associated with each specific helminth polyparasitism type.

## Methods

### Meta-analysis framework

We collected and synthesized information from three different types of data: Type I (single and multiple infection prevalence data), Type II (frequency of individuals infected with 0,1, 2, …, N parasite species), and Type III (association data).

### Search strategy and selection criteria

We searched the PubMed and Web of Science databases for studies published from inception to March 2017. We developed a search strategy using the following MeSH terms and keywords: “polyparasitism” AND “human”, “helminth” AND “malaria” OR “tuberculosis” OR “HIV”, “helminth” AND “coinfection” AND “human”, and “parasitic” AND “coinfection” AND “human.” We also identified additional references from the bibliographies of included studies.

Overall, study inclusion criteria are as follows: 1) study written in English, 2) study assessed human populations, 3) study included both sexes, and 4) standard diagnostic measures for helminths and the investigated co-infections were met. For tuberculosis, we excluded studies using the TB skin test due to the possibility of obtaining a false positive test from the Bacillus Calmette–Guérin vaccine. Due to the different objectives for each study type, specific inclusion and exclusion criteria for the different types of data are listed below.

Type I data evaluated the difference between single and multiple infection prevalence for helminth-helminth, helminth-intestinal protozoa, and helminth-malaria at the community level. To most accurately gain insight into the prevalence of co-infections that would be found in a community rather than a subset of the population, the relevant studies here had to meet the following criteria: 1) community- or school-based cross-sectional study design, 2) single and multiple co-infection data available for extraction, and 3) analysis of at least three helminth species for helminth-only, and two helminths for helminth-intestinal protozoa and helminth-malaria investigations.

Due to a paucity of community-based studies for helminth-HIV and helminth-TB studies, we assessed the mean difference of helminth-co-infected and HIV or TB singly infected individuals, respectively. This allowed the inclusion of additional study designs as well as studies conducted on subsets of the population. Inclusion criteria for these studies included: 1) case-control, cross-sectional, cohort or baseline randomized controlled trial study design, 2) provision of helminth prevalence among infected individuals, and 3) examination of at least two helminths. Studies were excluded if they focused on individuals presenting with diarrheal symptoms.

The Type II analysis evaluated the potential for interspecies interactions by comparing the observed species density frequency distributions to those expected assuming parasitic infection events are independent. Type II data used the same selection criteria as Type I, except that the Type II data required the number of individuals infected with 0,1, 2, …, N parasites and the prevalence of each individual parasite in the study community.

Type III studies providing association data had to meet the following criteria: 1) case-control, cross-sectional, cohort, or randomized controlled trial study design; 2) evaluation of associations between specific parasite pairs; and 3) provision of crude odds ratio, adjusted odds ratio, and/or data available to construct a 2x2 contingency table.

Identified titles and abstracts were examined by two independent reviewers (ZKC and RED). The full texts of potentially relevant articles were also evaluated by the same two reviewers. Articles meeting the inclusion criteria for the meta-analysis were subsequently screened for inclusion in the review of morbidity outcomes associated with polyparasitism.

### Meta-analysis methods

For Type I studies that provided single and multiple infection prevalence data, we generated corrected mean difference values, weighting for sample size using the correction statistic J as presented by Poulin [26]:

$$J=1-\frac{3}{4(N_{s}+N_{m}-2)-1}$$

The J values were then used to calculate the corrected mean difference (*d*) values:

$$d=J\;\left(\frac{crude\;mean\;difference,multiple-single}{100}\right)$$

Note, here for helminth-intestinal protozoa studies, we simply compared multiple versus single infection prevalences, irrespective of whether single infections were due to helminth or protozoan infection only. By contrast, for helminth-malaria, helminth-HIV, and helminth-TB, given the lack of information regarding single helminth infections, we compared the prevalence of helminth-malaria, helminth-HIV, and helminth-TB co-infected against malaria, HIV, and TB infection only, respectively.

For Type I helminth-malaria, helminth-HIV, and helminth-TB infected-only data, we additionally evaluated the prevalence of helminth infections among those harboring a malaria, HIV, or TB infection using the Freeman-Tukey double arcsine transformation [27] to address the problems of confidence limits extending beyond the 0,1 range and variance instability [28]. We back-transformed the results to proportions using a formula derived for the inverse of the Freeman-Tukey double arcsine transformation [29]. Heterogeneity between studies was assessed using the I^2^ statistic [30]. We used fixed effects models where heterogeneity was not significant (I^2^ <50%) and random-effects models for all other analyses. We used resampling methods to obtain bootstrapped 95% confidence intervals. We additionally conducted a meta-regression to evaluate the effect of a moderator variable, publication year, on the helminth-helminth polyparasitism mean difference outcome. All analysis was conducted using the ‘metafor’ package [31] in R statistical software version 3.4.1 [32].

To analyze Type II data, we compared the observed species density frequency distribution (number of individuals infected with 0,1, 2, …,N parasite species) from the collated field studies to the expected theoretical species density distribution computed using a null model developed by Janovy and colleagues to test for potential regularly occurring interspecies interactions [33]. This multiple-kind lottery model calculates the expected number of individuals infected with 0,1, 2, … N parasite species assuming independence of parasitic infection events and using the prevalence of a parasite species as the probability of infection success. The expected theoretical distribution was computed in this study via the implementation of the step-wise recurrence algorithm developed by Janovy and colleagues (S1 Text) [33]. The observed species density frequency distributions obtained directly from the studies were compared to the model-calculated expected distributions using chi-squared tests. Deviations in the observed data from the model-computed expected distribution can result from several processes, such as competitive interactions among parasite species or high host heterogeneity to infection [26].

The main summary measure used for Type III association data was the odds ratio (OR) [95% Confidence Interval (CI)]. Adjusted odds ratios were used preferentially, and the crude and adjusted odds ratios were analyzed separately in addition to pooled. Studies with zero-count cells were adjusted by adding 0.5 to all cell counts [34]. Data was entered as log OR and variance of the log OR and a fixed effects or random effects model was run in the ‘metafor’ package [31] depending on the existence of significant between-study heterogeneity.

Study quality was assessed via a quality score computed using the NIH Quality Assessment Tool for Observational Cohort and Cross-sectional Studies and the NIH Quality Assessment Tool for Case-Control Studies [35]. Quality assessment for cohort and cross-sectional study designs was conducted differently for the Types I and II data and the Type III data due to their different objectives. For Types I and II data, which assessed prevalence and species density distributions, questions 6, 7, 13, and 14 were not included in the quality assessment score as they were not applicable to cross-sectional prevalence studies. The quality assessment score for Type III association data, and Type I and III case-control studies included all questions. Quality assessment scores are reported as percentages obtained by dividing the number of studies reporting a “Yes” answer to each included question by the number of included questions.

Reporting bias was assessed using visual inspection of funnel plots and statistical evaluation using Egger’s regression test, where bias is evident when p<0.1 [36].

The following variables were extracted for all data: study design, age range, study site (country), treatment status of community, diagnostic method(s), and the data relevant to each type.

### Morbidity assessments

We undertook morbidity assessments by including any study that statistically evaluated the difference in a morbidity outcome between polyparasitized and singly parasitized individuals or polyparasitized and not polyparasitized individuals. Reported polyparasitism combinations were characterized as either having a positive, neutral or negative effect on the specified morbidity outcome. Positive and negative effects indicate the polyparasitized individuals experience a significantly better or worse health outcome, respectively, while neutral effects indicate the difference in morbidity outcomes was statistically insignificant. Chi-squared tests were conducted to determine if the total counts of observed positive, negative and neutral outcomes differed from those expected assuming the null hypothesis of equal proportions, which provides a vote-counting method based on deriving parameters for assessing outcomes against confidence intervals (α = 0.05) [23,37].

## Results

**Fig 1:**
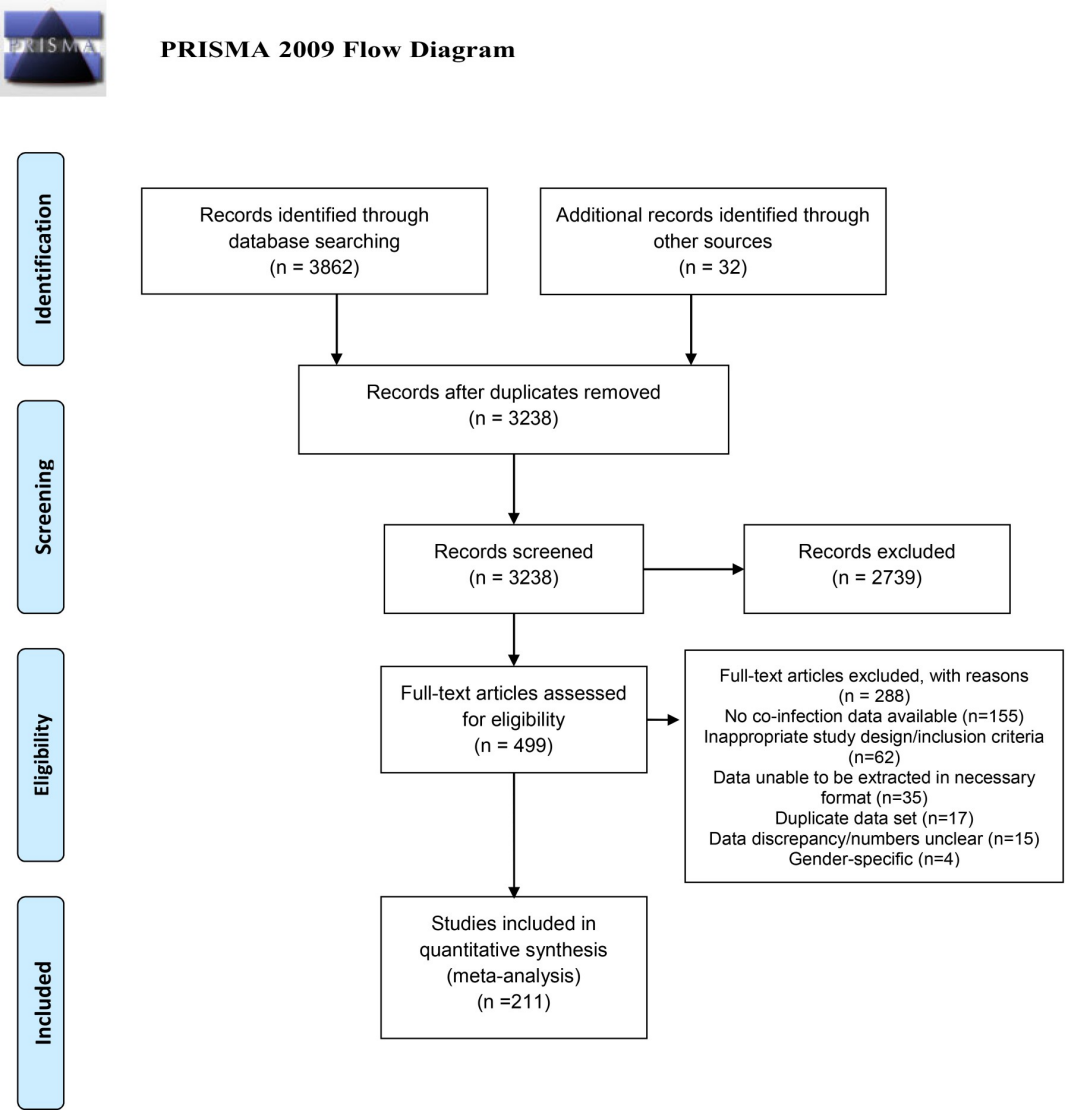
Flowchart of study selection process.

A total of 3862 studies were identified using the search strategy followed in this study (Methods). After removing duplicates and irrelevant studies (based on perusal of information given in study titles and abstracts), we conducted full-text article assessments for eligibility on 499 of these studies, of which 211 were subsequently included in the meta-analysis (Fig 1). An overview of study characteristics for each analysis performed is presented in Table 1, while tables of individual study characteristics can be found in the Supplementary Information (S1–S5 Tables).

**Table 1:** Overview of study characteristics for studies included in the meta-analyses performed for the three different types of data: Type I (single and multiple infection prevalence data), Type II (prevalence of host infection status class, from C = 0 for uninfected hosts to C = N for maximally-infected hosts), and Type III (association data). PSAC = pre-school aged children; SAC = school-aged children.

Data type	Parasite Combination	Number of studies	Study Population^+^	Continent	Single Infection*	Multiple Infection*	Refs
**Type I**	Helminth-Helminth	50	PSAC: 5 SAC: 20 Adults: 1 Combination: 23	Africa: 23 Asia: 20 North America: 3 South America: 4	2.8% - 58.0%	0.1% - 95.2%	[38–85]
	Helminth-Protozoa	40	PSAC: 4 SAC: 13 Adults: 1 Combination: 21	Africa: 11 Asia: 17 North America: 3 South America: 9	8.4% - 42.0%	1.1% - 87.3%	[45,47,53,59,86–120]
	Helminth-Malaria	15	PSAC: 1 SAC: 8 Adults: 0 Combination: 6	Africa: 13 Asia: 1 North America: 0 South America: 1	5.9% - 61.0%	3.4% - 64.1%	[63,66,75,121–132]
	Helminth-Tuberculosis	13	PSAC: 1 SAC: 0 Adults: 9 Combination: 3	Africa: 8 Asia: 2 North America: 0 South America: 3	NA	7.6% - 70.9%	[133–145]
	Helminth-HIV	23	PSAC: 0 SAC: 0 Adults: 14 Combination: 9	Africa: 18 Asia: 4 North America: 0 South America: 1	NA	1.9% - 69.4%	[146–168]
**Type II**	Helminth-Helminth	30	PSAC: 4 SAC: 14 Adults: 0 Combination: 12	Africa: 10 Asia: 15 North America: 2 South America: 3	2.8% - 58.0%	0.1% - 95.2%	[38,39,41–44,47,48,51–56,58,63,64,68,70,72,73,76–79,81–84]
	Helminth-Protozoa	18	PSAC: 4 SAC: 6 Adults: 0 Combination: 8	Africa: 4 Asia: 9 North America: 2 South America: 3	8.4% - 40.3%	1.5–78.3%	[47,53,86–92,95,97,101,102,110,112,113,116,119]
**Type III**	Helminth-Helminth	113	PSAC: 6 SAC: 49 Adults: 7 Combination: 51	Africa: 82 Asia: 23 North America: 0 South America: 8	NA	1.28–4.21	[41,45,48,49,51,52,60,63,64,66,75,80,81,85,95,99,104,106,110,111,115,124,131,169–191]
	Helminth-Malaria	56	PSAC: 6 SAC: 30 Adults: 1 Combination: 19	Africa: 53 Asia: 0 North America: 0 South America: 3	NA	0.84–1.49	[63,66,75,121,124,125,128,131,171,173,175,187,192–206]
	Helminth-Tuberculosis	16	PSAC: 0 SAC: 0 Adults: 9 Combination: 6	Africa: 14 Asia: 0 North America: 0 South America: 2	NA	1.31–1.88	[133,134,136,138,141,142,145,207]
	Helminth-HIV	45	PSAC: 0 SAC: 0 Adults: 13 Combination: 31	Africa: 31 Asia: 4 North America: 0 South America: 10	NA	0.88–2.13	[155,156,158,161–163,166,207–215]

*Refers to the range in prevalence for Type I and II prevalence studies and the range in computed odds ratios for Type III association studies

^+^ Combination refers to any combination of the age groups PSAC, SAC and Adults

For the 211 studies included in the meta-analysis, study quality was rated as either good (>70%), fair (50–70%), or poor (<50%) for each type of data for which a study met the inclusion criteria (S6 and S7 Tables). All studies for Type I and Type II data were rated as either good or fair and were thus included in the analysis. For Type III data, studies were rated in all three categories; those rated as poor were not included in the meta-analysis due to the significant risk of bias [35].

**Fig 2:**
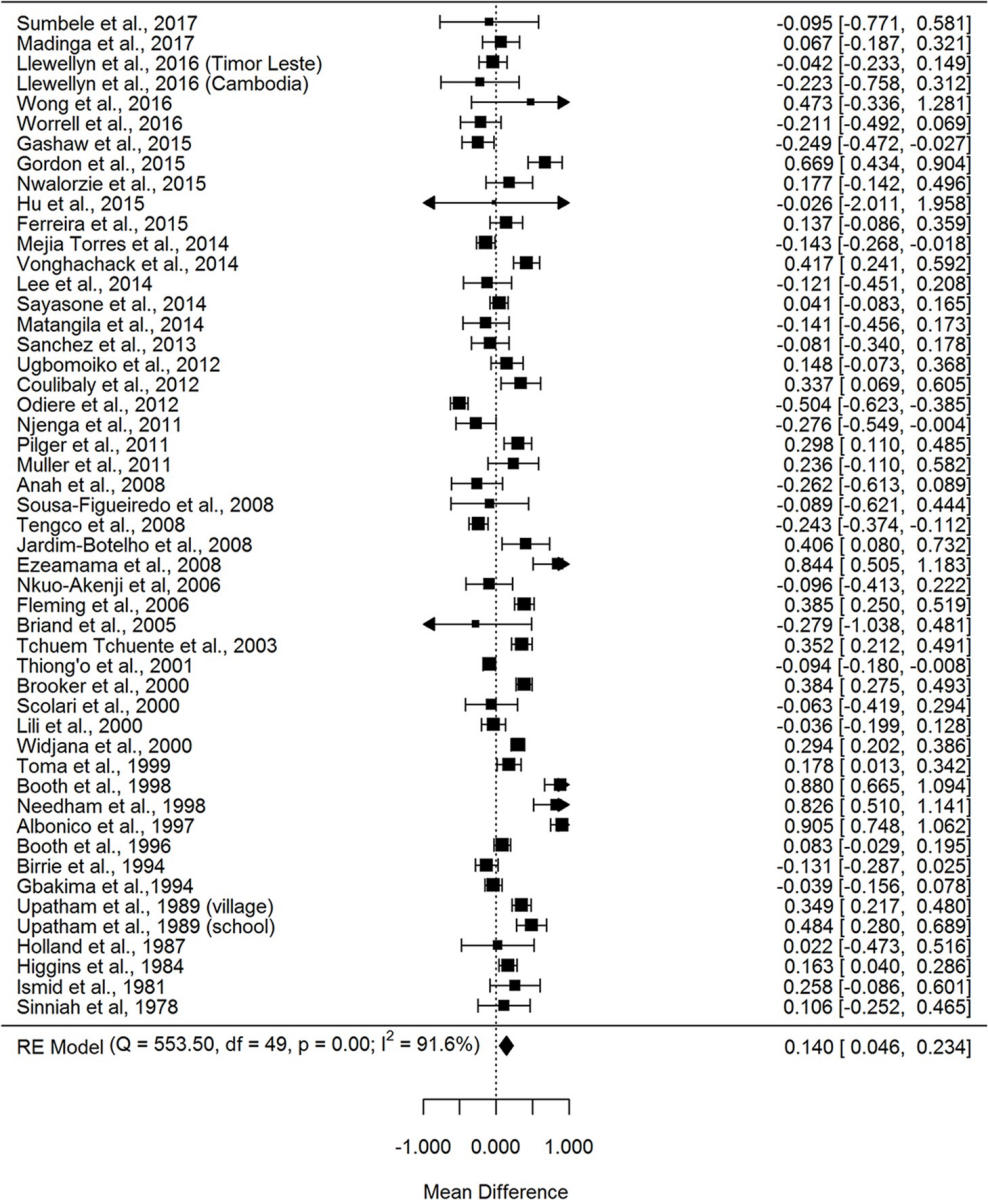
Forest plot of the mean community prevalence difference between humans infected with multiple helminth infections compared to humans infected with a single helminth infection. RE = random effects.

**Fig 3:**
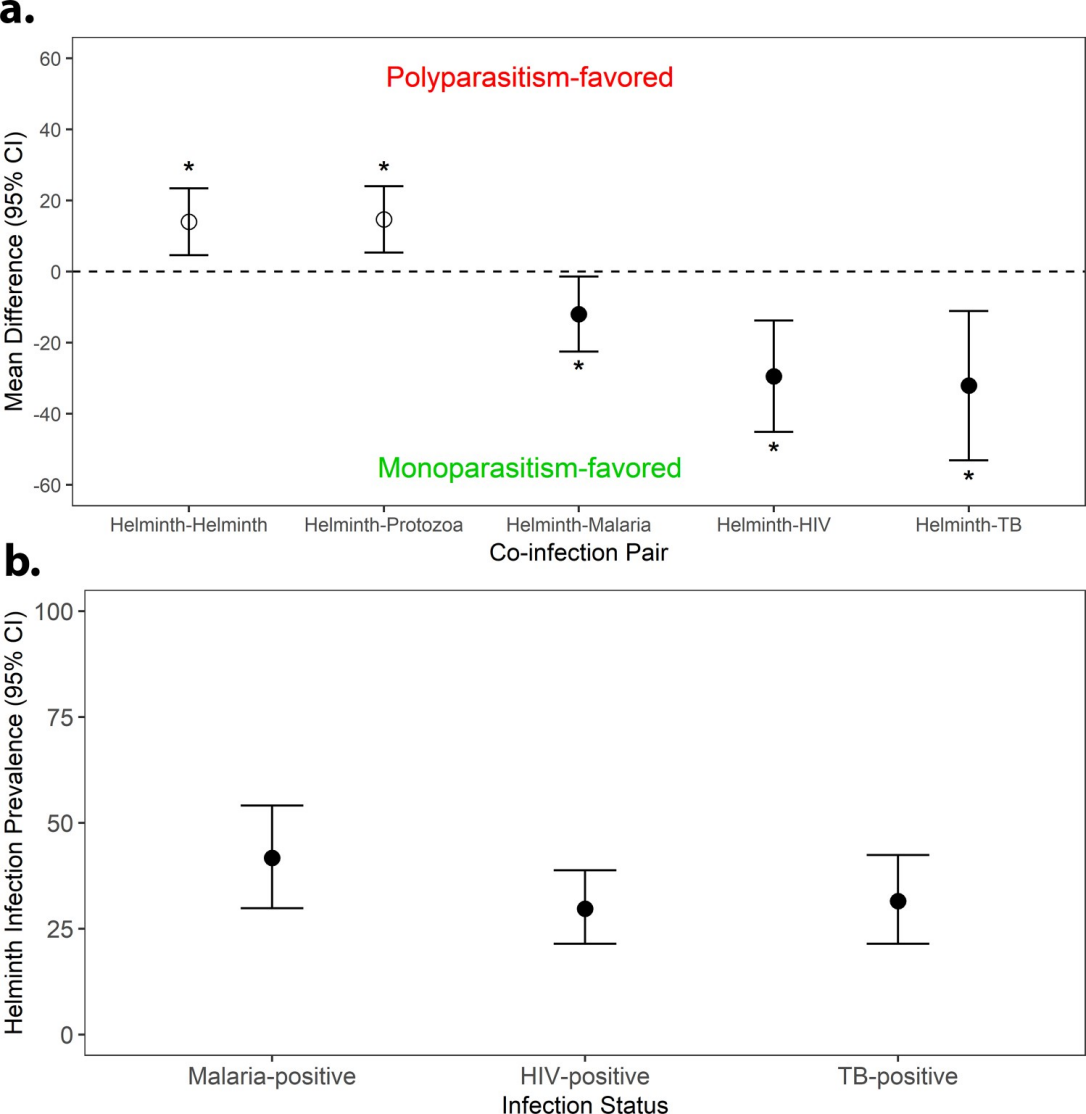
**Summary effect sizes and 95% confidence interval plots of (a) the mean prevalence difference between polyparasitized and monoparasitized human hosts with helminth-helminth, helminth-intestinal protozoa, helminth malaria, helminth-HIV, and helminth-TB infections and (b) the proportion of malaria-, HIV- and TB-infected individuals concurrently infected with at least 1 helminth**. Open circles indicate studies evaluated community-based differences in prevalence between multiply- and singly- infected individuals, while closed circles indicate studies evaluated the difference in prevalence between helminth co-infected and singly infected malaria, HIV, and TB positive individuals only, respectively. An asterisk denotes statistical significance at the 5% level.

**Fig 4:**
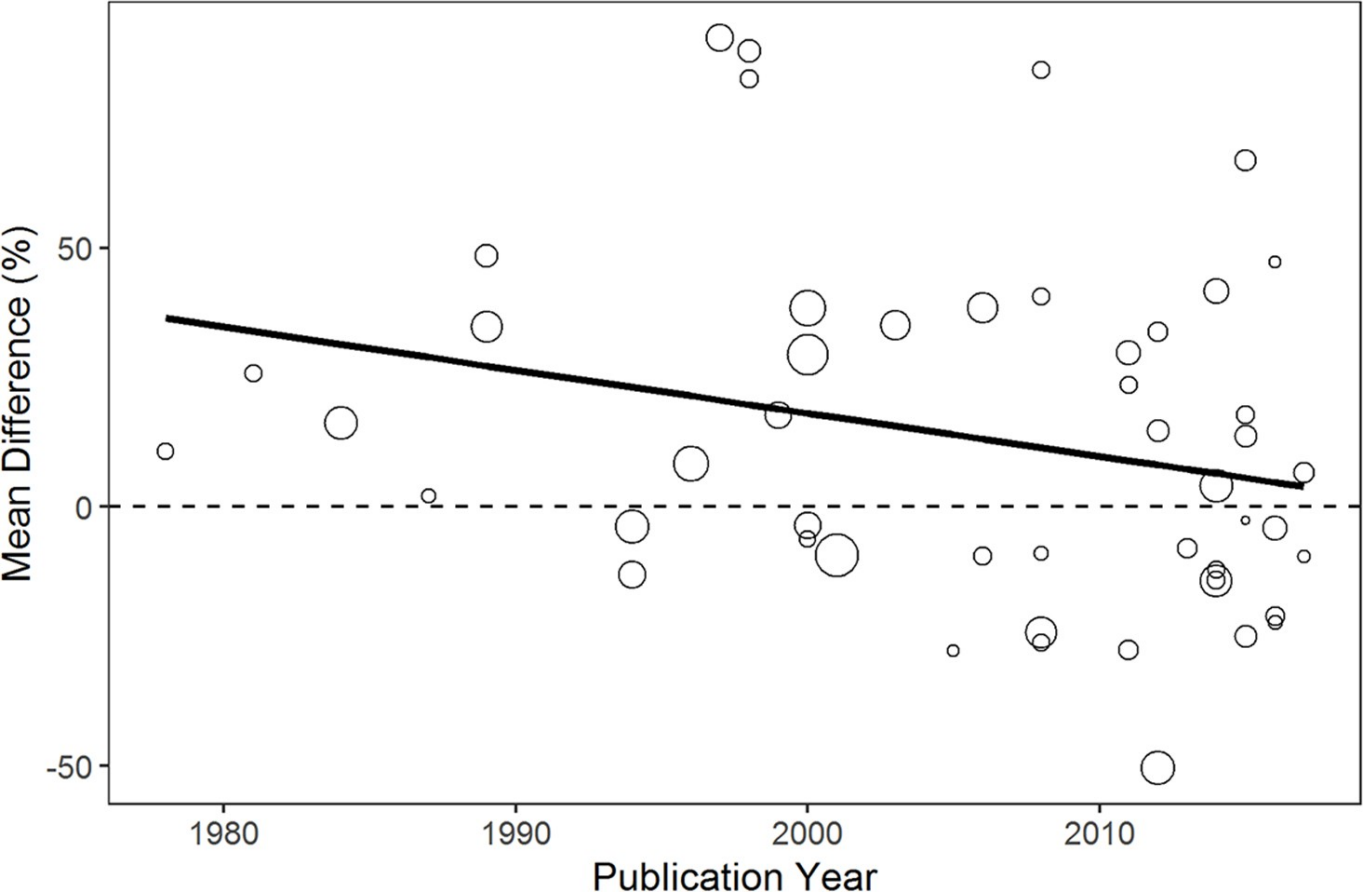
Random-effects univariate meta-regression between publication year and the mean difference between helminth-polyparasitized and helminth-monoparasitized individuals. Each circle represents a study and the circle size is representative of its weight (inversely proportional to the variance of that study) in the meta-regression analysis. The solid line indicates the regression prediction (coefficient -0.008 [95% CI -0.017–0.001], p = 0.072). The dashed line indicates an equal number of polyparasitized and monoparasitized individuals in the study, while study estimates above or below zero indicate a greater or lesser number of polyparasitized individuals when compared to monoparasitized individuals, respectively.

Studies meeting the inclusion criteria for the meta-analyses of the mean prevalence difference between multiple and single infections numbered 50 for helminth-helminth studies [38–85], 40 for helminth intestinal-protozoa studies [45,47,53,59,86–120], 15 for helminth-malaria studies [63,66,75,121–132], 13 for helminth-TB [133–145], and 23 for helminth-HIV [146–168]. All type I mean difference analyses were conducted used random effects models due to significant heterogeneity, ranging from a helminth-malaria I^2^ of 61.3% to a helminth-helminth I^2^ of 91.6% (Fig 2, S1–S4 Figs). The prevalence of polyparasitized helminth-helminth and helminth-protozoa individuals exceeded the prevalence of singly-infected helminth and protozoa individuals by 14.0% (95% CI 4.6–23.4%) and 14.7% (5.3–24.0%), respectively (Figs 2 and 3A, S1 Fig). For helminth-malaria, helminth-HIV, and helminth-TB, the prevalence of malaria-helminth, HIV-helminth and TB-helminth co-infected individuals was less than the prevalence of individuals singly-infected with malaria, HIV, and TB, respectively, with mean differences of -12.0% (-22.5 - -1.4%) for helminth-malaria, -29.5% (-45.1 - -13.8%) for helminth-HIV, and -32.1% (-53.1 - -11.1%) for helminth-TB (Fig 3A, S2–S4 Figs). However, it is important to note that among those infected with malaria, HIV, and TB, the prevalence of helminth infections was notable; among malaria-infected individuals, 41.7% (29.8–54.1%) were co-infected with at least one helminth infection (Fig 3B). Similarly, 31.5% (21.4–42.4%) of TB-positive individuals harbored at least one helminth infection and 29.7% (21.4–38.8%) of HIV-positive individuals were co-infected with at least one helminth infection (Fig 3B, S5–S7 Figs). The Egger’s Regression Test for Funnel Plot Asymmetry indicated bias for the mean difference Type I helminth-malaria studies (p = 0.085) and helminth-HIV studies (p = 0.007), but none for the helminth-only (p = 0.589), helminth-protozoa (p = 0.233), and helminth-TB (p = 0.520) studies. For the proportion of helminth co-infected individuals among those malaria-, HIV-, and TB-positive individuals, no bias was indicated for the helminth-malaria (p = 0.818), helminth-HIV (p = 0.361), or helminth-TB (p = 0.734) studies. Additionally, the meta-regression analysis indicated a downward trend in the helminth polyparasitism mean difference (helminth polyparasitism prevalence–helminth monoparasitism prevalence) over time (Fig 4), although this trend was only approaching significance (coefficient -0.008 [95% CI -0.017–0.001], p = 0.072).

**Fig 5:**
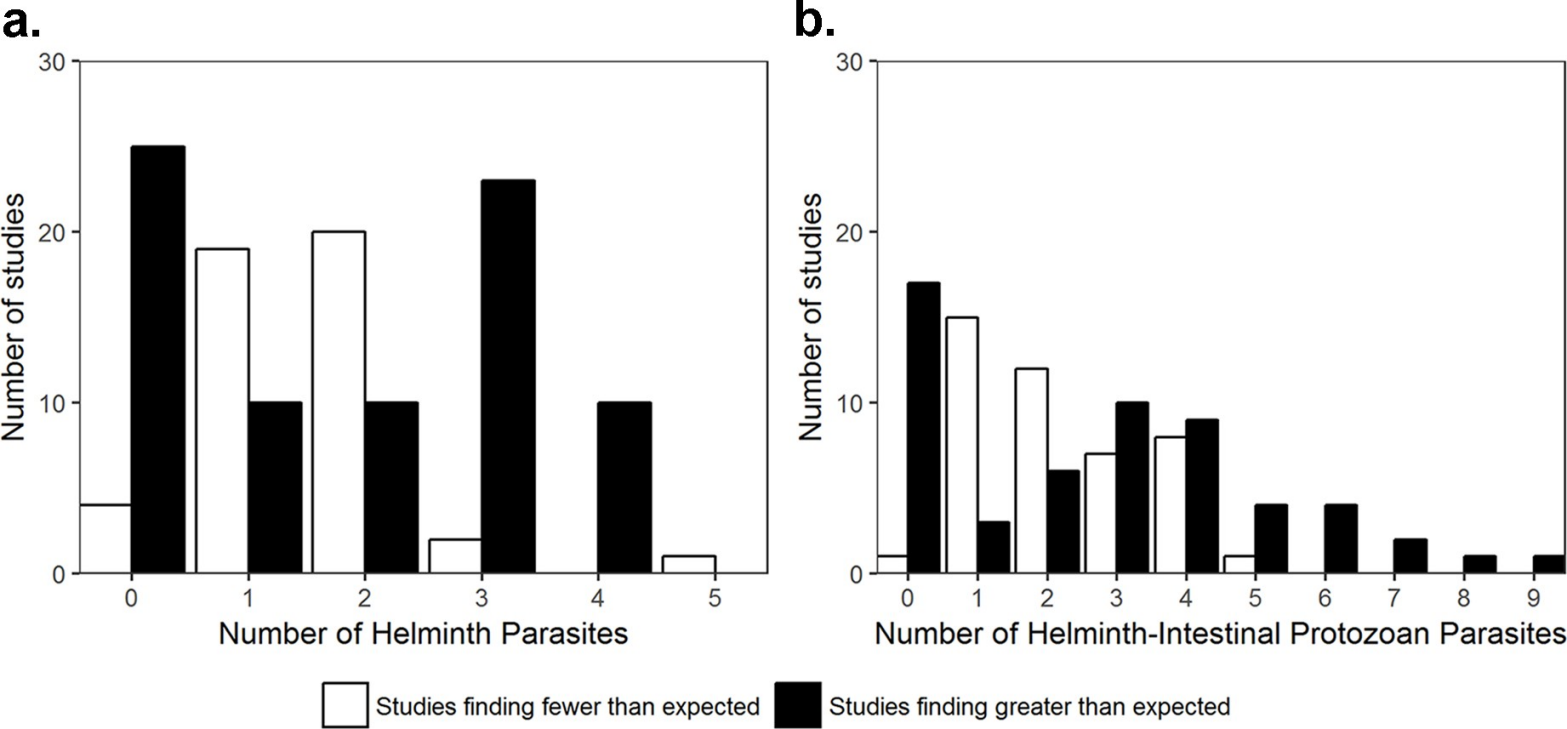
Number of studies finding fewer or greater individuals harboring the specified number of parasites compared to the expected number of individuals calculated using the Janovy null model for (a) helminth-only studies and (b) helminth-intestinal protozoa studies.

A total of 30 helminth-only [38,39,41–44,47,48,51–56,58,63,64,68,70,72,73,76–79,81–84] and 18 helminth-intestinal protozoa [47,53,86–92,95,97,101,102,110,112,113,116,119] studies provided categorical data concerning the number of parasites in each host and were evaluated using the Janovy model. Twenty studies demonstrated significantly different observed frequency distributions of infection compared to the frequency of host infection expected in each host class if infections were independent events for helminth-only studies, while ten studies demonstrated significant differences in these distributions for helminth-intestinal protozoa studies (Tables 2 and 3). For both helminth-only and helminth-protozoa infections, most studies found greater than expected numbers of individuals infected with zero and greater than two parasites, while the majority of studies found fewer than expected numbers of individuals infected with one and two parasites (Fig 5).

**Table 2:** Observed (O) and expected (E) species density frequency distributions of helminths in human hosts.

Study [reference]	O/E	total (n)	n = 0	n = 1	n = 2	n = 3	n = 4	n = 5	n = 6	n = 7	X^2^ Statistic	p-value
Wong et al., 2016 [84]	O	33	1	8	15	9					0.063	0.996
	E		1	8	16	9						
Gordon et al., 2015 [51]	O	545	10	85	196	188	66				4.434	0.3504
	E		6	81	208	191	59					
Hu et al., 2015 [54]	O	1403	1363	39	1	0					0.598	0.8968
	E		1362	41	0	0						
Ferreira et al., 2015 [47]	O	444	121	131	168	21	3	0	0		55.577	<0.001*
	E		81	204	142	17	1	0	0			
Vonghachack et al., 2014 [82]	O	729	81	172	276	169	31	0	0	0	48.268	<0.001*
	E		44	210	301	152	20	1	0	0		
Sanchez et al., 2013 [70]	O	320	88	129	76	27					47.863	<0.001*
	E		62	164	83	10						
Odiere et al., 2012 [68]	O	4064	1398	2356	296	13	1				1.769	0.778
	E		1399	2342	312	11	0					
Muller et al., 2011 [63]	O	156	17	51	76	11	1				10.949	0.027*
	E		9	65	72	10	0					
Anah et al., 2008 [39]	O	350	176	133	39	2					10.783	0.013*
	E		162	159	28	1						
Tengco et al., 2008 [77]	O	1990	879	797	293	21					139.413	<0.001*
	E		762	1016	206	6						
Jardim-Botelho et al., 2008 [56]	O	196	14	51	94	37					2.057	0.561
	E		10	56	93	37						
Fleming et al., 2006 [48]	O	1332	231	294	554	253	0	0	0	0	186.136	<0.001*
	E		116	458	547	209	5	0	0	0		
Briand et al., 2005 [43]	O	474	327	140	7	0	0				0.574	0.966
	E		329	136	9	0	0					
Tchuem Tchuente et al., 2003 [76]	O	1044	102	287	358	286	11				72.745	<0.001*
	E		60	293	458	229	5					** **
Thiong'o et al., 2001 [78]	O	3158	1017	1219	654	225	43				130.172	<0.001*
	E		891	1356	732	166	13					** **
Brooker et al., 2000 [44]	O	1738	146	462	542	485	103				132.655	<0.001*
	E		79	451	726	414	69					** **
Lili et al., 2000 [58]	O	766	190	302	197	77					41.157	<0.001*
	E		162	344	218	42						
Scolari et al., 2000 [72]	O	236	113	69	53	1					37.366	<0.001*
	E		94	111	30	1						
Widjana et al, 2000 [83]	O	2394	312	689	995	381	17				218.714	<0.001*
	E		175	843	1088	284	4					
Toma et al., 1999 [79]	O	654	60	239	241	114					20.663	0.001*
	E		49	234	287	84						
Booth et al., 1998 [41]	O	1539	3	91	541	904					7.352	0.061
	E		2	74	579	884						
Needham et al., 1998 [64]	O	543	8	43	233	259					18.919	<0.001*
	E		2	48	240	252						
Albonico et al., 1997 [38]	O	3497	1	167	979	2350					68.350	<0.001*
	E		2	99	1123	2272						
Booth et al., 1996 [42]	O	1276	45	563	569	99					0.975	0.807
	E		50	558	562	105						
Upatham et al., 1989 {[81]}	O	1142	92	326	444	280					194.919	<0.001*
	E		34	250	570	188						
Upatham et al., 1989 [81]	O	518	17	125	277	99					11.208	0.011*
	E		11	119	308	80						
Holland et al., 1987 [53]	O	140	77	30	20	12	1				74.177	<0.001*
	E		56	61	21	2	0					
Higgins et al., 1984 [52]	O	1387	325	418	368	276					275.53	<0.001*
	E		194	550	499	144						
Ismid et al., 1981 [55]	O	158	15	51	79	13					5.375	0.146
	E		10	59	80	9						
Sinniah et al., 1978 [73]	O	150	27	54	56	13					2.372	0.499
	E		23	63	52	12					** **	** **

* Indicates statistical significance (p<0.05)

**Table 3:** Observed (O) and expected (E) species density frequency distributions of helminth-intestinal protozoa parasites in human hosts.

Study [reference]	O/E	total (n)	0	1	2	3	4	5	6	7	8	9	10	11	12	13	14	15	X^2^ Statistic	p-value
Chin et al., 2016 [95]	O	186	41	75	51	18	1	0											12.111	0.033*
	E		26	84	60	14	1	0												
Al-Mekhlafi et al., 2016 [89]	O	1218	680	422	103	12	1	0	0	0									1.560	0.980
	E		671	436	100	10	0	0	0	0										
Mekonnen et al., 2016 [110]	O	1021	489	405	114	13	0	0	0	0	0	0	0						20.534	0.025*
	E		456	465	92	7	0	0	0	0	0	0	0							
Bless et al., 2015 [91]	O	228	68	83	52	19	6	0	0	0	0	0	0	0	0	0			13.593	0.403
	E		56	97	57	16	2	0	0	0	0	0	0	0	0	0				
Ahmad et al., 2014 [86]	O	131	118	11	2	0	0	0	0										1.651	0.949
	E		117	14	1	0	0	0	0											
Ferreira et al., 2015 [47]	O	444	59	127	178	69	11	0	0	0	0	0	0						8.714	0.559
	E		46	151	167	71	9	0	0	0	0	0	0							
Munoz-Antoli et al., 2014 [112]	O	382	27	56	79	78	65	36	25	11	3	2	0	0	0	0	0	0	152.890	<0.001*
	E		5	34	84	109	86	45	16	4	1	0	0	0	0	0	0	0		
Schar et al., 2014 [116]	O	218	27	64	72	36	15	3	1	0	0	0	0	0	0	0	0		4.154	0.994
	E		21	68	74	40	12	2	0	0	0	0	0	0	0	0	0			
Al-Delaimy et al., 2014 [88]	O	498	8	140	189	88	54	19	0										89.734	<0.001*
	E		5	114	207	132	36	4	0											
Boonjaraspinyo et al., 2013 [92]	O	253	159	79	15	0	0	0	0	0	0	0	0	0					2.186	0.998
	E		156	85	11	1	0	0	0	0	0	0	0	0						
Verhagen et al., 2013 [119]	O	390	126	122	89	46	7	0	0										29.274	<0.001*
	E		97	159	100	30	4	0	0											
Goncalves et al., 2011 [102]	O	133	94	30	9	0	0	0	0	0	0	0							15.201	0.086
	E		71	48	12	1	0	0	0	0	0	0								
Nematian et al., 2008 [113]	O	19209	15675	3150	365	19	0	0	0	0	0	0							134.055	<0.001*
	E		15519	3453	231	6	0	0	0	0	0	0								
Al-Agha et al., 2000 [87]	O	209	119	77	10	3	0	0											5.797	0.327
	E		120	74	15	1	0	0												
Gamboa et al., 1998 [101]	O	292	132	96	37	19	6	1	1	0	0	0							51.209	<0.001*
	E		105	124	51	10	1	0	0	0	0	0								
Chunge et al., 1991 [97]	O	1129	212	250	234	230	134	52	13	4	0	0	0	0					240.781	<0.001*
	E		99	299	362	239	98	27	5	1	0	0	0	0						
Holland et al., 1987 [53]	O	140	65	34	23	17	1	0	0										40.890	<0.001*
	E		45	59	29	6	1	0	0											
Annan et al., 1986 [90]	O	422	126	130	97	47	21	1	0	0	0	0	0	0					76.434	<0.001*
	E		83	172	121	39	6	1	0	0	0	0	0	0						

* Indicates statistical significance (p<0.05)

**Fig 6:**
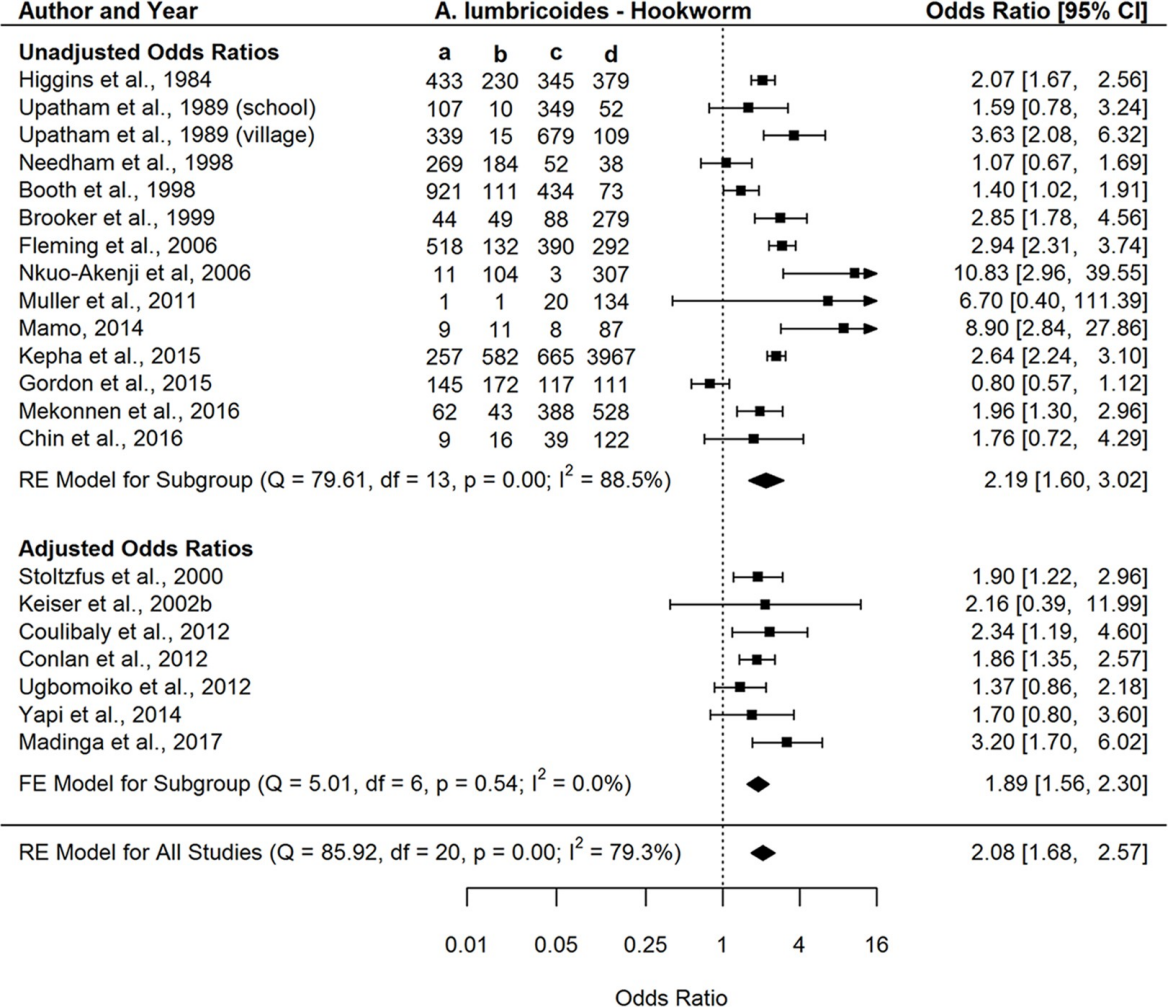
Forest plot of the association between *A*. *lumbricoides* and hookworm infections stratified by studies reporting unadjusted and adjusted odds ratios. a = AL+/HW+; b = AL+/HW-; c = AL-/HW+; d = AL-/HW-; RE = random effects. Odds ratio compares the odds of *A*. *lumbricoides* infection among hookworm*-*positive individuals (a/c) to the odds of *A lumbricoides* infection among hookworm-negative individuals (b/d).

**Fig 7:**
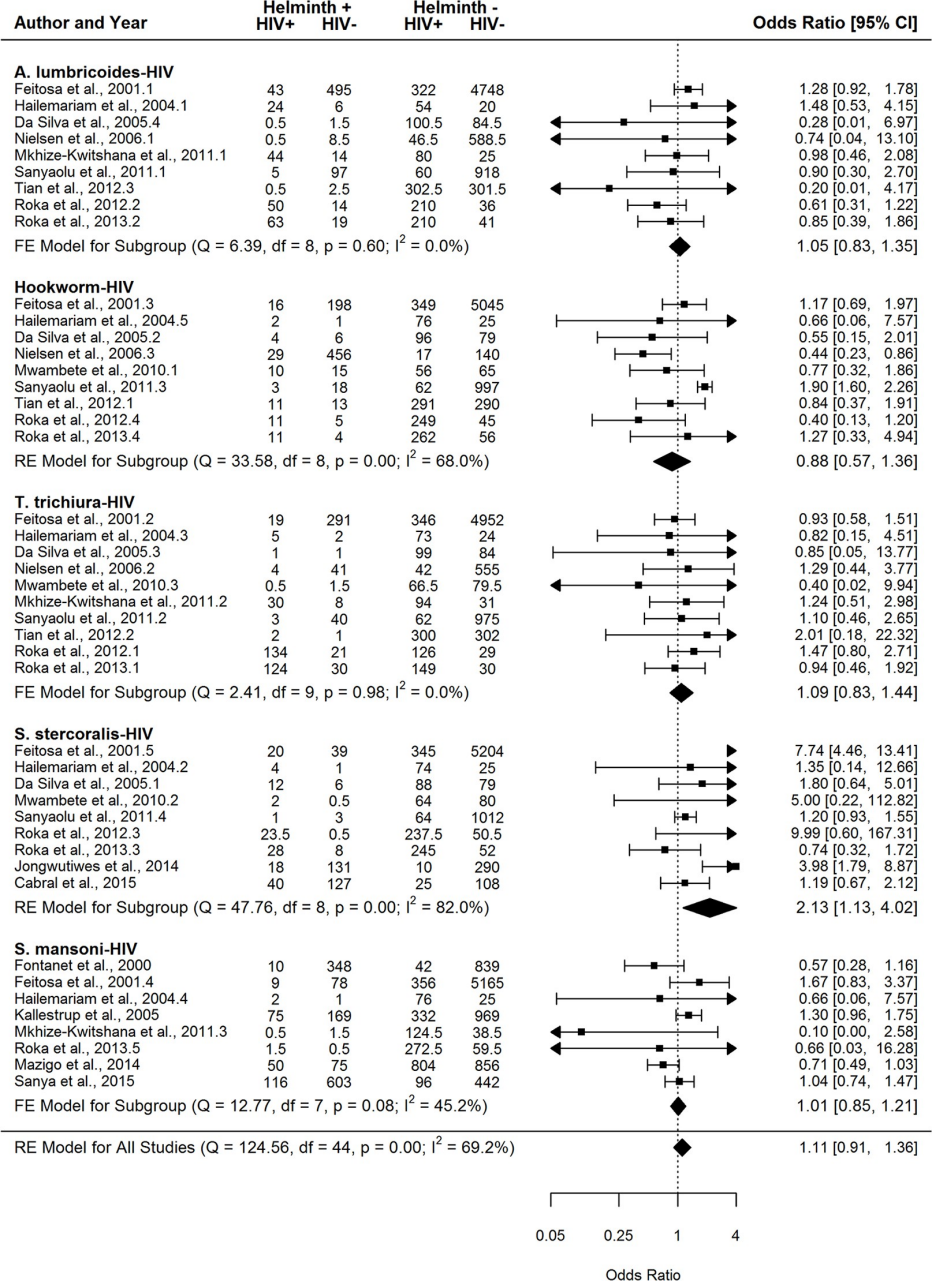
Forest plots for the meta-analyses comparing the association between the helminth infections *A*. *lumbricoides*, hookworm, *T*. *trichiura*, *S*. *stercoralis*, *S*. *mansoni* and HIV infection. FE = fixed effects; RE = random effects.

**Fig 8:**
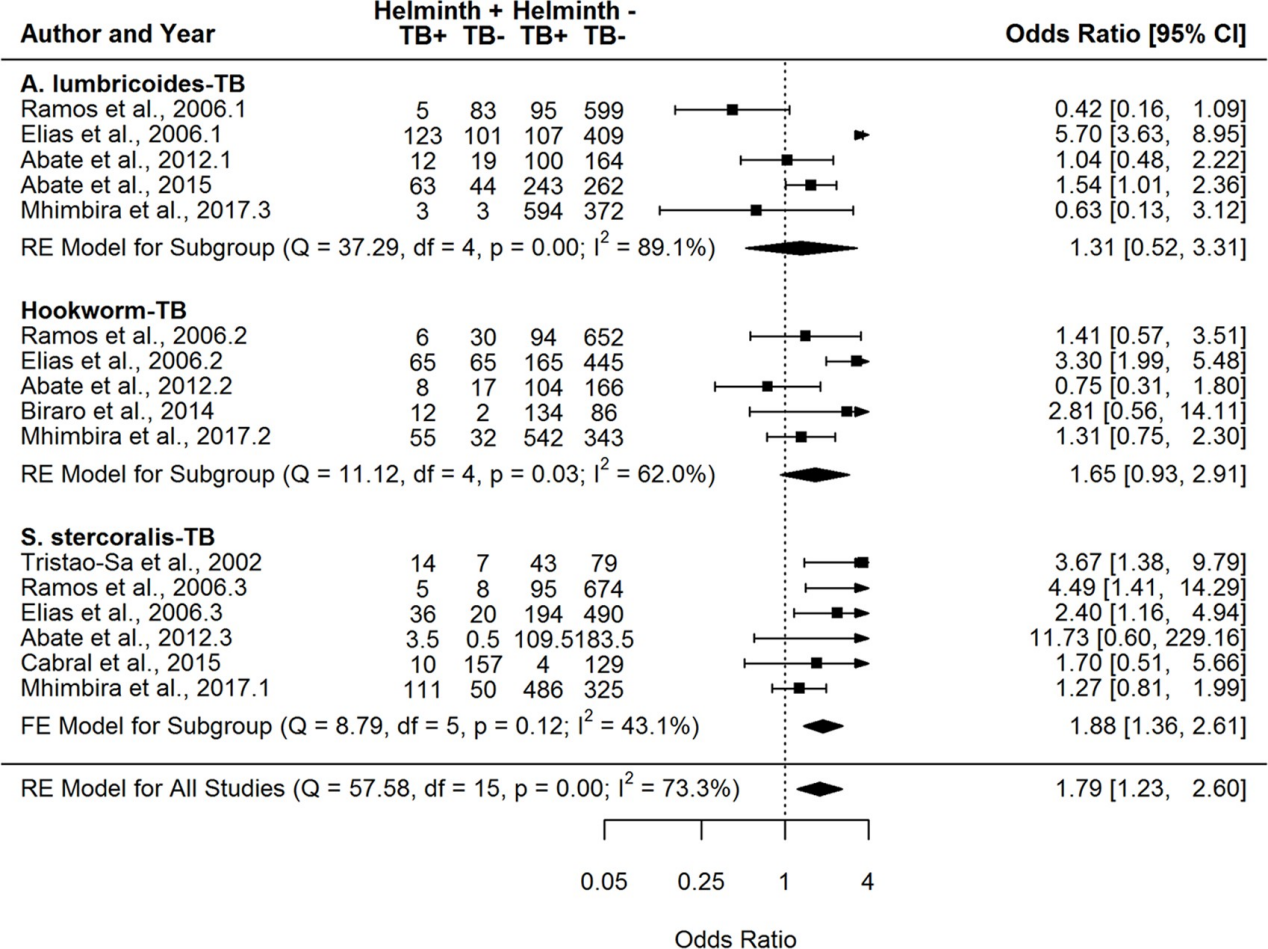
Forest plots for the meta-analyses comparing the association between the helminth infections *A*. *lumbricoides*, hookworm, *S*. *stercoralis* and tuberculosis (TB) infection. FE = fixed effects; RE = random effects.

A total of eight helminth-helminth, five helminth-malaria, five helminth-HIV, and three helminth-TB parasite pairs had at least five studies with study quality ≥50% and were thus included in the Type III meta-analyses (Table 4). Both fixed and random-effects models were run for the above parasite pairs based on absence or presence of significant between-study heterogeneity given that I^2^ values for the different parasite pairs varied from 0% to 90%. Seven of the eight helminth-only pairs demonstrated a significant positive association (Table 4, Fig 6 and S8–S14 Figs), with the *A*. *lumbricoides-T*. *trichiura* pairing overall showing the association of highest magnitude. Our findings indicate that *S*. *stercoralis* was the only parasite found to be significantly positively associated with both HIV and TB (OR 2.13 [1.13–4.02] and 1.88 [1.36–2.61], respectively) (Figs 7 and 8), while hookworm and *S*. *mansoni* were the two parasites found to be significantly positively associated with malaria (OR 1.35 [1.08–1.69] and OR 1.49 [1.04–2.14], respectively) (Table 4 and S15–S19 Figs). Overall, no parasite pairs exhibited a statistically significant negative association. The following parasite pairs exhibited bias based on Egger’s test: hookworm-HIV (p = 0.054), *S*. *stercoralis*-TB (p = 0.015), *A*. *lumbricoides*-hookworm (p = 0.054), *T*. *trichiura*-hookworm (p = 0.078), and *T*. *trichiura*-*S*. *stercoralis* (p = 0.001).

**Table 4:** Summary of computed odds ratios representing the association between helminth species and other helminth species, malaria, HIV, and TB.

Parasite pair	Overall odds ratio	References
**Helminth-Helminth**		
*A*. *lumbricoides* + Hookworm	2.08 (1.68–2.57)*	[41,45,48,51,52,60,63,64,66,80,81,95,110,124,131,171,172,176,179,189]
*T*. *trichiura* + Hookworm	2.58 (1.84–3.89)*	[41,52,60,64,66,80,81,95,110,131,171,172,176,178,179,186,189]
*A*. *lumbricoides* + *T*. *trichiura*	4.21 (3.21–5.52)*	[41,45,52,60,64,66,75,80,81,85,95,99,110,111,115,131,170–172,174,179,184,188,189,191]
*A*. *lumbricoides* + *S*. *mansoni*	1.29 (0.87–1.91)	[48,49,63,80,110,176,179,180,183,187]
*T*. *trichiura* + *S*. *mansoni*	1.68 (1.10–2.55)*	[45,80,110,176,179,180,183,187]
Hookworm + *S*. *mansoni*	1.74 (1.28–2.37)*	[48,63,80,104,106,110,131,175,176,179,182,184,185,187]
*T*. *trichiura* + *S*. *stercoralis*	2.43 (1.27–4.66)*	[60,80,110,172,179,186]
*S*. *haematobium* + *S*. *mansoni*	2.19 (1.02–4.73)*	[45,63,80,104,169,173,175,177,181,190]
**Helminth-Malaria**		
Malaria + *T*. *trichiura*:	0.87 (0.71–1.07)	[66,75,128,131,194,195,199,202,206]
Malaria + *A*. *lumbricoides*:	0.84 (0.64–1.08)	[63,66,75,124,131,192,194,195,199,202,206]
Malaria + Hookworm:	1.35 (1.08–1.69)*	[63,66,121,124,125,128,131,171,194,195,197,199,201,202,204–206]
Malaria + *S*. *mansoni*:	1.49 (1.04–2.14)*	[63,175,187,198,201,202]
Malaria + *S*. *haematobium*:	1.34 (0.92–1.97)	[63,121,128,131,173,193,196,200,202,203]
**Helminth-HIV**		
HIV + *T*. *trichiura*:	1.09 (0.83–1.44)	[156,158,161–163,166,208,210,213,215]
HIV + *A*. *lumbricoides*:	1.05 (0.83–1.35)	[156,161–163,166,208,210,213,215]
HIV + Hookworm:	0.88 (0.57–1.36)	[158,161–163,166,208,210,213,215]
HIV + *S*. *mansoni*:	1.01 (0.85–1.21)	[155,156,162,208–210,212,214]
HIV + *S*. *stercoralis*	2.13 (1.13–4.02)*	[158,161–163,207,208,210,211,215]
**Helminth-TB**		
TB+ *S*. *stercoralis*	1.88 (1.36–2.61)*	[133,138,141,142,145,207]
TB + Hookworm	1.65 (0.93–2.91)	[133,136,138,141,142]
TB + *A*. *lumbricoides*	1.31 (0.52–3.31)	[133,134,138,141,142]

* Indicates statistical significance (p<0.05)

**Fig 9:**
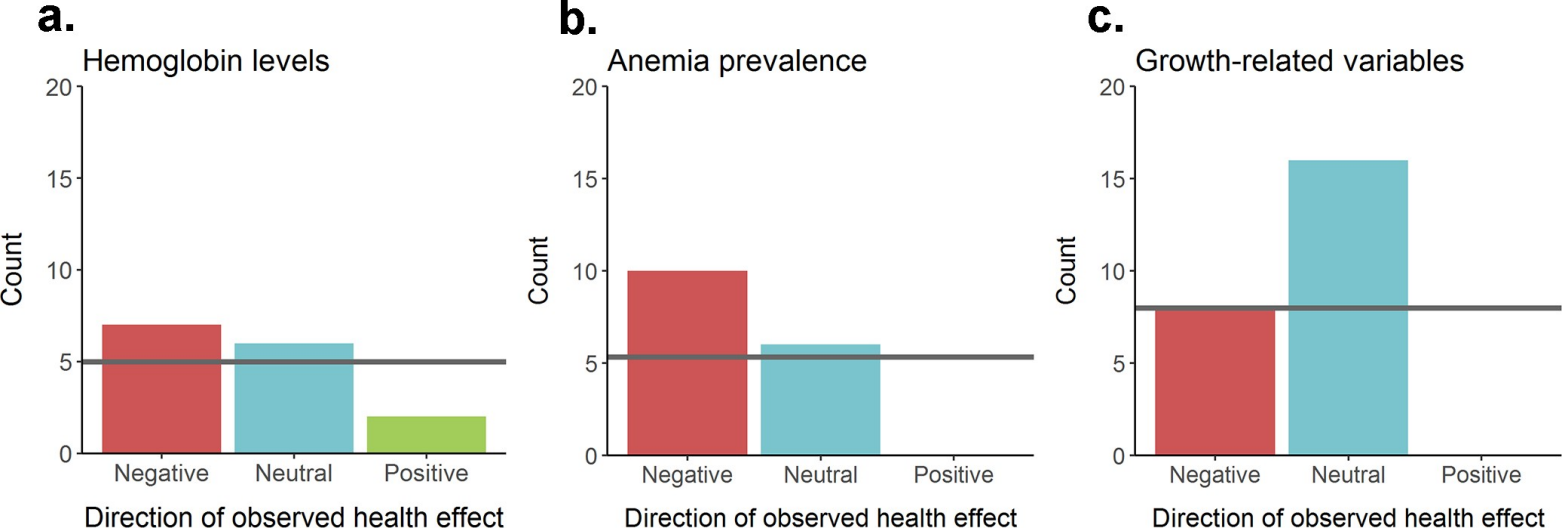
**Direction of reported health outcomes of helminth co-infections for (a) hemoglobin levels, (b) anemia prevalence, and (c) growth-related variables.** Horizontal line indicates expected value assuming the null hypothesis of equal proportions.

From the studies included in our meta-analysis, we identified three broad groups of morbidity-related outcomes for which multiple studies existed (Table 5): anemia prevalence, hemoglobin levels, and growth-related outcomes (Fig 9). Nine studies reporting differences in anemia prevalence between individuals with multiple infections compared to individuals with either single infections or no multiple infections showed 10 negative, 6 neutral, and 0 positive effects respectively on human health (Table 5). Of the studies providing data on the difference in hemoglobin levels, the most common observation was that multiply-infected individuals had significantly lower hemoglobin levels; we classified 7 negative, 6 neutral, and 2 positive effects on human health from 11 studies. Seven studies provided information on growth-related outcomes; from these studies we classified 8 negative, 16 neutral, and 0 positive effects from a range of indicators including BMI, stunting, age-for-height, and weight-for-height. The pattern of observed effects was significantly different than that expected assuming the null model of equal proportions for anemia prevalence (X^2^ = 9.5, df = 2, p = 0.009) and growth-related factors (X^2^ = 16.0, df = 2, p<0.001), while the hemoglobin levels pattern was not statistically significant (X^2^ = 2.8, df = 2, p = 0.247).

**Table 5:** Summary of morbidity outcomes reported by studies included in this meta-analysis which statistically evaluated the difference between polyparasitized and singly parasitized or not polyparasitized individuals. MI = multiple infection; SI = single infection; NI = no infection; H = helminth; M = malaria; P = intestinal protozoa; TB = tuberculosis; HIV = human immunodeficiency virus; Hb = hemoglobin; SH = *S*. *haematobium*; HW = hookworm; HN = *H*. *nana*; TT = *T*. *trichiura*; AL = *A*. *lumbricoides*; PF = *P*. *falciparum*; SCH = schistosomiasis; STH = soil-transmitted helminths; IPs = Intestinal parasites; BMI = body mass index; S = significant; NS = not significant; y/o = years old; OR = odds ratio; AOR = adjusted odds ratio; PR = prevalence ratio; MD = mean difference.

Morbidity Outcome	Study [reference]	Parasite Combination	Comparison	Specific Comparison	Statistical Analysis	Significance	Meaning
Anemia	Ezeamama et al., 2008 [46]	H-H	MI vs not MI	MI (Moderate intensity) vs SI (low intensity) or NI	OR	2/3 S	**MI ↑**
	Adedoja et al., 2015 [121]	H-H	MI vs not MI	SH+HW+ vs not; SH+HN+ vs not; HW+HN+ vs not	OR	1/3 S	**2/3 MI ↑(1S)**; *1/3 MI ↓*
	Sanchez et al., 2013 [70]	H-H	MI vs SI vs NI	MI vs SI vs NI for AL, TT, and HW	X^2^	NS	MI < SI but > NI
	Burdam et al., 2016 [123]	H-M	MI vs SI	M+H+ vs SI	OR/AOR	OR S/ AOR NS	**MI ↑**
	Sumbele et al., 2017 [75]	H-M-H/M	MI vs SI	MI (PF+AL+ or PF+TT+ or AL+TT+) vs SI	X^2^	S	**MI ↑**
	Adedoja et al., 2015 [121]	H-M	MI vs not MI	PF+SH+ vs not; PF+HW+ vs not; PF+HN+ vs not	OR	3/3 S	**MI ↑**
	Humphries et al., 2011 [197]	H-M	MI vs not MI	HW+M+ vs not	OR	NS	**MI ↑**
	Njua-Yafi et al., 2016 [127]	H-M	MI vs not MI	M+H+ vs not	OR	NS	*MI ↓*
	Arndt et al., 2013 [147]	H-HIV	MI vs SI	HIV+/H+ vs HIV+/H-	PR	S	**MI ↑**
	Idindili et al., 2011 [153]	H-HIV	MI vs SI	HIV+ Helminths only vs HIV+H-	AOR	S	**MI ↑**
Hb levels	Midzi et al., 2010 [126]	H-H	MI vs SI	SCH vs. SCH+STH+	MD	S	**MI ↓**
	Matangila et al., 2014 [61]	H-H	MI vs SI vs NI	MI vs SI vs NI	ANOVA	S	**MI lowest**
	Sanchez et al., 2013 [70]	H-H	MI vs SI vs NI	MI vs SI vs NI	ANOVA	NS	MI < SI but > NI
	Muller et al., 2016 [111]	H-H	MI vs SI vs NI	AL+TT+ vs AL+TT- vs AL-TT+ vs AL-TT-	X^2^	S	MI and 1 SI lowest
	Pullan et al., 2010 [204]	H-M	MI vs not MI	HW+M+ vs not	Unclear	S	**MI ↓**
	Matangila et al., 2014 [61]	H-M	MI vs not MI	H+M+ vs not	t-test	S	**MI ↓**
	Sanchez-Arcila et al., 2014 [129]	H-M	MI vs SI	M+IPs+ vs IPs+M-	ANOVA	NS	**MI ↓**
	Sumbele et al., 2017 [75]	H-M	MI vs SI	(H-H or H-M) vs (H or M)	Mann Whitney U-test	S	**MI ↓**
	Midzi et al., 2010 [126]	H-M	MI vs SI	SCH vs. PF+SCH+STH+	MD	S	**MI ↓**
	Kung'u et al., 2009 [199]	H-M		Interaction term of H*M predictor for Hb score	Regression	NS	
	Righetti et al., 2012 [205]	H-M	MI vs SI	PF+/HW+ vs PF+	t-test	S: 8y/o; NS: 7y/o, 6y/o	*MI ↑*: *8y/o*, *7y/o;* **MI ↓: 6y/o**
	Arndt et al., 2013 [147]	H-HIV	MI vs SI	HIV+/H+ vs HIV+/H-	PR	S	**MI ↓**
	Mhimbira et al., 2017 [142]	H-TB	MI vs SI	TB+/H+ vs TB+/H-	Unclear	S	*MI ↑*
Stunting	Saldiva et al., 1999 [115]	H-H, H-P	MI vs not MI	TT+/AL+ vs not; TT+/GL+ vs not; AL+/GL+ vs not	OR/AOR	1 S, 1 NS, 1 OR S/AOR NS	**MI ↑**
	Sanchez et al., 2013 [70]	H-H	MI vs SI vs NI	MI vs SI vs NI for AL, TT, and HW	X^2^	NS	**MI highest**
	Muller et al., 2016 [111]	H-H	MI vs SI vs NI	AL+TT+ vs AL+TT- vs AL-TT+ vs AL-TT-	Unclear	S	**MI highest**
Height	Muller et al., 2016 [111]	H-H	MI vs SI vs NI	AL+TT+ vs AL+TT- vs AL-TT+ vs AL-TT-	Unclear	S	MI and 1 SI lowest
	Nematian et al., 2008 [113]	H-P	MI vs SI	MI (3) vs MI [121] and MI [121] vs SI	t-test	NS	**MI lowest**
Height-for-age	Sanchez et al., 2013 [70]	H-H	MI vs SI vs NI	MI vs SI vs NI for AL, TT, and HW	ANOVA	NS	**MI lowest**
	Quihui-Cota et al., 2004 [114]	H-P	MI vs not MI	MI vs not MI for H and/or P	ANOVA	S	**MI ↓**
Weight	Nematian et al., 2008 [113]	H-P	MI vs SI	MI (3) vs MI [121] and MI [121] vs SI	t-test	NS	**MI lowest**
	Mhimbira et al., 2017 [142]	H-TB	MI vs SI	TB+/H+ vs TB+/H-	Unclear	NS	**MI ↓**
	Muller et al., 2016 [111]	H-H	MI vs SI vs NI	AL+TT+ vs AL+TT- vs AL-TT+ vs AL-TT-	Unclear	S	MI and 1 SI lowest
Weight-for-age	Sanchez et al., 2013 [70]	H-H	MI vs SI vs NI	MI vs SI vs NI for AL, TT, and HW	ANOVA	S	**MI ↓**
Weight-for-height	Quihui-Cota et al., 2004 [114]	H-P	MI vs not MI	MI vs not MI for H and/or P	Z score	S	**MI ↓**
BMI	Muller et al., 2016 [111]	H-H	MI vs SI vs NI	AL+TT+ vs AL+TT- vs AL-TT+ vs AL-TT-	Unclear	S	1 SI lowest
	Mhimbira et al., 2017 [142]	H-TB	MI vs SI	TB+/H+ vs TB+/H-	Fisher’s Exact	NS	**MI lowest**
	Alemu et al., 2017 [135]	H-TB	MI vs SI	TB+/H+ vs TB+/H-	OR/AOR	S	**↓ BMI more likely to be MI**
BMI-for-age	Sanchez et al., 2013 [70]	H-H	MI vs SI vs NI	MI vs SI vs NI for AL, TT, and HW	ANOVA	NS	**MI lowest**
% thin	Sanchez et al., 2013 [70]	H-H	MI vs SI vs NI	MI vs SI vs NI for AL, TT, and HW	X^2^	NS	MI and SI highest
% underweight	Sanchez et al., 2013 [70]	H-H	MI vs SI vs NI	MI vs SI vs NI for AL, TT, and HW	X^2^	NS	**MI highest**
% wasted	Muller et al., 2016 [111]	H-H	MI vs SI vs NI	AL+TT+ vs AL+TT- vs AL-TT+ vs AL-TT-	Unclear	NS	MI and 1 SI highest
Body fat %	Mhimbira et al., 2017 [142]	H-TB	MI vs SI	TB+/H+ vs TB+/H-		S	**MI ↓**
MUAC	Mhimbira et al., 2017 [142]	H-TB	MI vs SI	TB+/H+ vs TB+/H-		NS	*MI ↑*
Waist hip ratio	Mhimbira et al., 2017 [142]	H-TB	MI vs SI	TB+/H+ vs TB+/H-		NS	MI = SI

In the ‘meaning’ column, bold text denotes the morbidity outcome was worse among those multiply-infected, while italicized text denotes the morbidity outcome was better among those multiply-infected.

## Discussion

Although studies have suggested the ubiquity of polyparasitism in the tropics [20,21], a systematic assessment of the frequency, magnitude, direction and clinical outcome of co-infections between the major human helminths and other pathogens has been lacking. This is despite increasing recognition that host co-infection with multiple pathogens is the norm, and that a better quantitative understanding of the nature and extent of polyparasitism can have important epidemiological, clinical and control implications [3,5,14,22,23].

Here, we have conducted analyses of the available published data on the occurrence of helminth polyparasitism to provide a first comprehensive assessment of the extent, nature and health consequences of helminth co-infection in humans. Our results indicate overall that co-infection with helminths is generally more prominent and produces poorer host health outcomes compared with single infections, irrespective of the diversity of inter-parasite associations studied, although this outcome is less apparent in the case of some interspecies infections (Fig 3, Table 4), or type of data by which these co-infections are reported. Thus, our meta-analyses of infection prevalence data demonstrated that helminth polyparasitism was significantly more abundant than single infections for both helminth-helminth (d = 14.0%; CI 4.6–23.4%) and helminth-intestinal protozoa (d = 14.7%; CI 5.3–24.0%) infections (Fig 3A). By contrast, while this predilection for a higher level of co-infection was not found for malaria, TB, and HIV infections, it is notable that helminthiasis was still common among those hosts infected with these pathogens (Fig 3B). Similarly, assessment of the frequency distribution of species richness among different host classes revealed that for both helminth-helminth and helminth-intestinal protozoa studies, single and double infections are observed less than expected by chance, while uninfected host classes and host classes with greater than two species occurred more frequently than expected (Fig 5). Finally, our analysis of the direction and magnitude of the interspecies associations recorded (Table 4) show that while the majority of evaluated pairs of helminths were found to be significantly positively associated, signifying those infected with a specific helminth were significantly more likely to be infected with another compared to uninfected hosts, we found *S*. *mansoni* and hookworm to be the only two helminths significantly positively associated with malaria, whereas *S*. *stercoralis* was the only helminth exhibiting a significant positive association with TB and HIV.

A multitude of factors acting at various hierarchical levels from the within-host infra-parasite community level to the higher host community level could explain the observed positive associations between specific helminth and helminth, malaria, TB, and HIV pairs; such factors may include similar transmission routes, genetically-modified and immunologically-mediated host responses to infection, overlapping environmental distribution of parasite fauna, and commonly occurring social risk factors [216–219]. At the individual host level, an additional consideration is interspecies interactions, where specific helminth species can either interact within the human host with both other worms and microparasites directly in a negative or positive manner or act to regulate co-infections top-down via interactions with the host immune system [14,19]. If these bottom-up or top-down interspecies interactions among parasites in a host community are common and strong, then the distribution of within-host infracommunity species richness would not be expected to simply reflect the prevalences of the various parasite species. Thus, the findings based on the Janovy null model analysis of the interspecies associations among helminth-helminth and helminth-protozoa communities, which showed in general that more studies reported a greater than expected numbers of individuals with zero infections or infected with greater than two parasites while the majority of studies found fewer than expected numbers of individuals infected with one or two parasites (Fig 5), could be due to shared common transmission routes [220,221], or modifications affected by either direct interactions between parasites or via the host immune system [17,18].

With regard to the involvement of helminth-mediated top-down control of microparasites through the immune system, several studies have suggested that helminth infection may alter host susceptibility to TB [5]; one study not only found an association between helminths and TB but noted associations of increasing magnitude with an increasing number of helminths harbored [138]. Our study finding of positive associations between helminths and TB, with *S*. *stercoralis* being significant, supports this observation. By contrast, the effect of helminth co-infections on the clinical presentation of TB is not conclusive; some studies have found no significant effects of helminth infection on TB severity [134,137,222], while one study demonstrated that TB-helminth co-infected individuals have been found to have more advanced clinical presentation [144], although the extent to which this can be attributed to helminth-induced immunity changes or larval migration through the lungs remains unclear [5]. A study which found that deworming may result in a significant improvement in pro-inflammatory cytokine responses in latent-TB infected individuals which may reduce disease progression from latent to active TB suggests the importance of helminth-induced immunity changes in disease progression [223].

Researchers have hypothesized that helminth infections might increase one’s susceptibility to HIV due to the helminth-induced strong T helper 2 (Th2) response and downregulation of the antiviral T helper 1 (Th1) response [224–226]; a recent study provided prospective data demonstrating lymphatic filariasis increased the likelihood of HIV infection [227]. Our findings of predominantly positive associations, although only one was significant, provide support to this hypothesis. It is to be noted, here, that in addition to immunological factors, detrimental physical conditions, such as anemia and malnutrition, which are associated with helminthiasis, may also increase susceptibility to HIV and disease progression to AIDS [5]. A recent review on the effect of deworming medications on HIV disease progression concluded that while deworming of HIV-infected adults may positively affect HIV disease progression markers in a small and short-term manner, more research is needed to better understand this result [228].

Likewise for malaria, helminth-induced alteration of the balance between Th1 and Th2 type immune responses may increase susceptibility to malaria, although helminth-induced immunity is also thought to protect against severe complications of malaria [5,229]. However, population studies have provided conflicting reports of the relationship between helminths and malaria [229], although a recent review suggested these conflicting reports might be due to differences in the association of individual helminths with malaria [230], which is additionally reflected in this meta-analysis.

Ecological research into assembly rules structuring within-host parasite infracommunities suggests that apart from the action of factors at the host level, species richness in such parasite assemblages may also reflect the outcome of forces acting at the broader host community level [218]. Such factors may range from environmental and climatic variables that govern the biogeography of parasite and host species, including latitudinal gradient effects [218,231], epidemiological factors, such as exposure intensity, herd immunity and population density, to socio-economic factors that underlie host community sensitivity and adaptive response to parasitic infection [232–235]. This macroecological perspective to unravelling and predicting observed species richness in parasite assemblages means that investigative frameworks that can integrate species interactions at the within-host level with factors that govern parasite richness at the broader host community and ecological levels need to be developed and applied if we are to better understand the forces that govern the observed helminth polyparasitic patterns uncovered in this study. This will also include the derivation and evaluation of process-driven hierarchical approaches if better mechanistic understandings of the transmission and control of the human helminths are to be ultimately achieved [18].

Of the studies included in this meta-analysis which evaluated morbidity outcomes, most exhibited negative effects of helminth co-infections on hemoglobin levels and anemia prevalence (Table 5; Fig 9). While the etiology of anemia is multifactorial [236], many of the diseases studied in this meta-analysis are known to contribute to anemia, including malaria, schistosomiasis, hookworm, HIV and tuberculosis [237–240]. The proposed mechanisms by which these various diseases contribute to anemia vary, but an additive effect of such co-infections seems likely [22]. Thus, the finding that helminth co-infections are overwhelmingly associated with negative health outcomes of both higher anemia prevalences and lower hemoglobin levels is not unexpected. By contrast, the analyses evaluating growth-related variables were mostly neutral in outcome, although all statistically significant results were negative with no significant positive effects reported (Table 5). Malnutrition and associated poor growth outcomes have been found to be associated with helminth and intestinal protozoan infections [241,242], which comprise the bulk of the reviewed health effects, and thus the finding of either neutral or negative outcomes in this study is similarly not surprising. Nevertheless, the consistency of these detrimental effects observed across the range of pathogens investigated indicates that multiple infections associated with helminths generally result in worsened health outcomes. This result suggests that the health burden of helminthiases may be significantly underestimated currently. It also implies that more systematic holistic data on the outcomes of helminth polyparasitism, including co-infection with pathogens types that were not represented in the present studies, will be required if more accurate estimates of helminth disease burden is to be quantified.

A positive finding for disease control efforts from this study is that helminth-helminth polyparasitism prevalence appears to be decreasing over time (Fig 4), although this finding was only approaching significance (p = 0.072). This general trend can likely be attributed to deworming programs being instituted in endemic countries, although development may also be contributing to this decline. Overall, this result suggests the benefit of continuing deworming programs to reduce the prevalence of helminth polyparasitism. However, this analysis was limited by the dependence in this study on published data in the literature; while this meta-analysis was based on epidemiological studies conducted in endemic countries, these collated studies are not necessarily representative of the different geographies as they were not designed to obtain a representative sample of helminth prevalence within a political boundary. Routine surveillance data with a consistent approach to measuring and reporting polyparasitism would provide more accurate estimates and allow for additional analysis of trends in the data.

These study findings also have important implications for global health interventions seeking to alleviate the disease burden. There is a tendency in medicine and public health to consider infectious diseases in isolation [21]; however, the findings of this paper challenge this inclination. Not only is polyparasitism common in the tropics, potential interactions between helminths and other co-infections may also exacerbate both susceptibility to and disease progression of major infectious diseases including malaria, TB, and HIV. Co-infected individuals largely exhibit more severe morbidity outcomes than those either singly infected or not co-infected. While the exact mechanisms by which helminths interact with microparasites to affect host susceptibility and disease progression require further research, our findings of frequent co-infections and positive associations support the call for integrating deworming into routine treatment of malaria, HIV, and TB [243–245]. While there recently has been an effort to integrate treatments of the helminthic neglected tropical diseases [243–245], the call to integrate deworming into malaria, TB, and HIV treatment protocols has largely gone unanswered [246]. A recent mathematical modelling exercise suggested that a mass drug administration strategy reducing lymphatic filariasis transmission could potentially increase malaria prevalence, underscoring the importance of taking an integrated approach to disease control [15,16]. Overall, our study results suggest that new community ecology-based frameworks that can combine biomedical research into interspecies interactions at the individual host level with epidemiological, social, and ecological studies of factors that drive parasite species diversity at the host community level, will be ultimately needed if we are to shed better light on the direct and indirect processes that structure within-host parasite communities, parasite pathology, and on methods to accomplish the control of such communities [13,14,18].

This meta-analysis has several limitations. Firstly, Egger’s regression test indicated seven of the twenty-nine analyses presented here exhibited reporting bias. This bias could be attributed to publication bias, whereby the results are influenced by the publication or non-publication of studies, or to language bias as we only accepted studies published in English. For three of the analyses with potential reporting biases, (Type I helminth-HIV and Type III hookworm-HIV and *T*. *trichiura*-hookworm), larger studies indicated higher prevalence or odds ratio. However, for the other four analyses (Type I helminth-malaria, Type III *S*. *stercoralis*-TB, *A*. *lumbricoides*-hookworm, and *T*. *trichiura*-*S*. *stercoralis*) the prevalence of co-infections and the associations may be overestimated as larger studies indicated lower prevalence or odds ratios. The asymmetry noted in the funnel plots could also be due to true heterogeneity, whereby there are differences in underlying risk in the different sampled communities [36]. An additional limitation of this study is that the diagnostic method used to detect helminths was predominantly microscopic examination of stool samples using the Kato-Katz technique [247]; this method is known to underestimate the prevalence of single and multiple helminth infections, particularly when only a single stool sample is conducted and in areas of low intensity infections [248,249]. Therefore, this meta-analysis likely underestimated the true prevalence of helminth polyparasitism and co-infections. An additional limitation is that Type III studies were largely cross-sectional in nature which precludes temporal analysis to evaluate if a specific parasitic infection affects susceptibility to an additional parasitic infection. Finally, the analysis is further limited by the lack of a consistent approach to studying polyparasitism and analyzing polyparasitism data as evidenced by the multiple analyses we conducted and the ineligibility of many studies for all types of analysis. A consistent methodology to quantify and evaluate polyparasitism would provide improved estimates of its magnitude and allow for additional analyses of patterns that could inform more targeted interventions to combat polyparasitism.

## Supporting information

S1 Checklist PRISMA checklist. (DOC)

S1 Text More detailed methodology for meta-regression and Type II data analysis. (DOCX)

S1 Table Study characteristics of Type I and II helminth-helminth studies included in the meta-analysis. (DOCX)

S2 Table Study characteristics of Type I and II helminth-protozoa studies included in the meta-analysis. (DOCX)

S3 Table Study characteristics of Type I helminth-malaria studies included in the meta-analysis. (DOCX)

S4 Table Study characteristics of Type I helminth-tuberculosis (TB) studies included in the meta-analysis. (DOCX)

S5 Table Study characteristics of Type I helminth-HIV studies included in the meta-analysis. (DOCX)

S6 Table Quality assessment scores for each study considered in this meta-analysis using the NIH Quality Assessment Tool for Observational Cohort and Cross-sectional Studies. (DOCX)

S7 Table Quality assessment scores for each study considered in this meta-analysis using the NIH Quality Assessment Tool for Case-Control Studies. (DOCX)

S1 Fig Mean community prevalence difference between humans infected with multiple helminth-intestinal protozoa infections compared to humans infected with a single helminth or intestinal protozoa infection. (TIF)

S2 Fig Mean community prevalence difference between humans co-infected with helminth and malaria infections compared to humans infected with a single malaria infection. (TIF)

S3 Fig Mean community prevalence difference between humans co-infected with helminth and HIV infections compared to humans infected with a single HIV infection. (TIF)

S4 Fig Mean community prevalence difference between humans co-infected with helminth and TB infections compared to humans infected with a single TB infection. (TIF)

S5 Fig Forest plot for the meta-analysis evaluating the proportion of helminth co-infected individuals among malaria-positive individuals. (TIF)

S6 Fig Forest plot for the meta-analysis evaluating the proportion of helminth co-infected individuals among HIV-positive individuals. (TIF)

S7 Fig Forest plot for the meta-analysis evaluating the proportion of helminth-infected individuals among tuberculosis (TB)-positive individuals. (TIF)

S8 Fig Forest plot for the meta-analysis comparing the association between *A. lumbricoides* (AL) and *T. trichiura* (TT). a = AL+/TT+; b = AL+/TT-; c = AL-/TT+; d = AL-/TT-; RE = random effects. Odds ratio compares the odds of *A*. *lumbricoides* infection among *T*. *trichiura-*positive individuals (a/c) compared to the odds of *A lumbricoides* infection among *T*. *trichiura*-negative individuals (b/d).
(TIF)

S9 Fig Forest plot for the meta-analysis comparing the association between *T. trichiura* (TT) and hookworm (HW). a = TT+/HW+; b = TT+/HW-; c = TT-/HW+; d = TT-/HW-; RE = random effects. Odds ratio compares the odds of *T*. *trichiura* infection among hookworm*-*positive individuals (a/c) compared to the odds of *T*. *trichiura* infection among hookworm-negative individuals (b/d).
(TIF)

S10 Fig Forest plot for the meta-analysis comparing the association between *T. trichiura* (TT) and *S. stercoralis* (SS). a = TT+/SS+; b = TT+/SS-; c = TT-/SS+; d = TT-/SS-; RE = random effects. Odds ratio compares the odds of *T*. *trichiura* infection among *S*. *stercoralis-*positive individuals (a/c) compared to the odds of *T*. *trichiura* infection among *S*. *stercoralis*-negative individuals (b/d).
(TIF)

S11 Fig Forest plot for the meta-analysis comparing the association between *A. lumbricoides* (AL) and *S. mansoni* (SM). a = AL+/SM+; b = AL+/SM-; c = AL-/SM+; d = AL-/SM-; RE = random effects. Odds ratio compares the odds of *A*. *lumbricoides* infection among *S*. *mansoni-*positive individuals (a/c) compared to the odds of *A lumbricoides* infection among *S*. *mansoni*-negative individuals (b/d).
(TIF)

S12 Fig Forest plot for the meta-analysis comparing the association between *S. mansoni* (SM) and hookworm (HW). a = SM+/HW+; b = SM+/HW-; c = SM-/HW+; d = SM-/HW-; RE = random effects. Odds ratio compares the odds of *S*. *mansoni* infection among hookworm*-*positive individuals (a/c) compared to the odds of *S*. *mansoni* infection among hookworm-negative individuals (b/d).
(TIF)

S13 Fig Forest plot for the meta-analysis comparing the association between *T. trichiura* (TT) and *S. mansoni* (SM). a = TT+/SM+; b = TT+/SM-; c = TT-/SM+; d = TT-/SM-; RE = random effects. Odds ratio compares the odds of *T*. *trichiura* infection among *S*. *mansoni-*positive individuals (a/c) compared to the odds of *T*. *trichiura* infection among *S*. *mansoni*-negative individuals (b/d).
(TIF)

S14 Fig Forest plot for the meta-analysis comparing the association between *S. haematobium* (SH) and *S. mansoni* (SM). a = SH+/SM+; b = SH+/SM-; c = SH-/SM+; d = SH-/SM-; RE = random effects. Odds ratio compares the odds of *S*. *haematobium* infection among *S*. *mansoni-*positive individuals (a/c) compared to the odds of *S*. *haematobium* infection among *S*. *mansoni*-negative individuals (b/d).
(TIF)

S15 Fig Forest plot for the meta-analysis comparing the association between *A. lumbricoides* (AL) and malaria (M). a = AL+/M+; b = AL+/M-; c = AL-/M+; d = AL-/M-; RE = random effects. Odds ratio compares the odds of *A*. *lumbricoides* infection among malaria-positive individuals (a/c) compared to the odds of *A*. *lumbricoides* infection among malaria-negative individuals (b/d).
(TIF)

S16 Fig Forest plot for the meta-analysis comparing the association between hookworm (HW) and malaria (M). a = HW+/M+; b = HW+/M-; c = HW-/M+; d = HW-/M-; RE = random effects. Odds ratio compares the odds of hookworm infection among malaria-positive individuals (a/c) compared to the odds of hookworm infection among malaria-negative individuals (b/d).
(TIF)

S17 Fig Forest plot for the meta-analysis comparing the association between *T. trichiura* (TT) and malaria (M). a = TT+/M+; b = TT+/M-; c = TT-/M+; d = TT-/M-; RE = random effects. Odds ratio compares the odds of *T*. *trichiura* infection among malaria-positive individuals (a/c) compared to the odds of *T*. *trichiura* infection among malaria-negative individuals (b/d).
(TIF)

S18 Fig Forest plot for the meta-analysis comparing the association between *S. haematobium* (SH) and malaria (M). a = SH+/M+; b = SH+/M-; c = SH-/M+; d = SH-/M-; RE = random effects. Odds ratio compares the odds of *S*. *haematobium* infection among malaria-positive individuals (a/c) compared to the odds of *S*. *haematobium* infection among malaria-negative individuals (b/d).
(TIF)

S19 Fig Forest plot for the meta-analysis comparing the association between *S. mansoni* (SM) and malaria (M). a = SM+/M+; b = SM+/M-; c = SM-/M+; d = SM-/M-; RE = random effects. Odds ratio compares the odds of *S*. *mansoni* infection among malaria-positive individuals (a/c) compared to the odds of *S*. *mansoni* infection among malaria-negative individuals (b/d).
(TIF)
