# Supplementary material for: The extent, nature, and pathogenic consequences of helminth polyparasitism in humans: A meta-analysis
Source: PLoS Negl Trop Dis. 2019 Jun 18;13(6):e0007455. doi: 10.1371/journal.pntd.0007455 (PMC6599140; doi:10.1371/journal.pntd.0007455)
Supplement: S1 Text — (DOCX) [file pntd.0007455.s002.docx]

**Type I analysis**

*Meta-regression method*

Meta-regression models are used to evaluate the effect of study characteristics on the effect estimates.

The meta-regression model we used is a mixed-effects model which for this analysis can be written as:

$$y_{i}=\beta_{0}+\beta_{1}x_{i1}+u_{i}+e_{i}$$

where $u_{i} \sim N(0,\tau^{2}$) and $e_{i}\sim N (0,v_{i})$, $x_{i1}$represents the value of the publication year moderator variable for the *i*th study, $\beta_{0}$ represents the average outcome when the value of the publication year moderator variable equals zero, $\beta_{1}$represents how the average mean difference changes for a unit increase in publication year, $x_{i1}$, $u_{i}$ is a random effect describing the study-specific deviation from the distribution mean, and $e_{i}$ is a random error term describing sampling variability.

The regression model is weighted using the standard inverse-variance method where the weights are equal to $w_{i}=1/(\tau^{2}+v_{i})$, where $\tau^{2}$ represents the amount of residual heterogeneity in the outcomes and $v_{i}$represents the within-study variance (Viechtbauer, 2015).

**Type II analysis**

The type II analysis was conducted to evaluate whether inter-species interactions might be occurring in helminth-helminth and helminth-intestinal protozoa communities. By comparing the observed species density distribution (the number of humans infected with 0, 1, 2, … *N* parasites) with an expected species density distribution assuming no interactions, significantly different distributions indicate inter-species interactions may be occurring. The observed species density distribution was obtained directly from individual studies, while the expected species density distributions were computed using a multiple-kind lottery model which uses a recurrence vector algorithm developed by Janovy and colleagues (1995).

In the recurrence vector algorithm described below, $N$= the number of parasite species added to the distribution, ${P(n)}_{N}$ = the probability of infection by *n* parasite species when $N$ parasite species have been added to the distribution, $p_{i}$= probability of successful infection by the *i*th parasite species, $q_{i}$ (equivalent to ${1-p}_{i}$) represents the probability of no infection by the *i*th parasite species.

The addition of the first parasite species establishes the vector:

For $N=1$:

${P\left( 0 \right)}_{N}= {P\left( 0 \right)}_{1}=q_{N}=q_{1}$,

${P\left( 1 \right)}_{N}= {P\left( 1 \right)}_{1}=p_{N}=p_{1}$.

As additional parasite species are added, three algorithms are used to recalculate the expected distribution. For all parasite species added after the first, where the host class *C* goes from 0, 1, 2, … N,

If $C=0$:

${P\left( C \right)}_{N}= {P\left( 0 \right)}_{N}={P(0)}_{N-1}q_{N}$;

If $0<C<N$:

${P\left( i \right)}_{N}= {P\left( i-1 \right)}_{N-1}p_{N}+{P(i)}_{N-1}q_{N}$ for all *i,* where *i* goes from 1 to $N-1$;

If $C=N$:

${P\left( C \right)}_{N}= {P\left( N \right)}_{N}={P(N-1)}_{N-1}p_{N}$.

**References**

Janovy Jr, J., Clopton, R. E., Clopton, D. A., Snyder, S. D., Efting, A., & Krebs, L. (1995). Species density distributions as null models for ecologically significant interactions of parasite species in an assemblage. *Ecological Modelling*, *77*(2-3), 189-196.

Viechtbauer, W. (2015). Package ‘metafor’. *The Comprehensive R Archive Network. Package ‘metafor’. http://cran. r-project. org/web/packages/metafor/metafor. pdf*.
