# Supplementary material for: The extent, nature, and pathogenic consequences of helminth polyparasitism in humans: A meta-analysis
Source: PLoS Negl Trop Dis. 2019 Jun 18;13(6):e0007455. doi: 10.1371/journal.pntd.0007455 (PMC6599140; doi:10.1371/journal.pntd.0007455)
Supplement: S1 Table — (DOCX) [file pntd.0007455.s003.docx]

**S1 Table.** **Study characteristics of Type I and II helminth-helminth studies included in the meta-analysis.** QA = Quality Assessment; CS = Cross-sectional; UC= unclear.

| **QA Score** | **Study Author and Publication Year** | **Study Population** | **Study Design** | **Age Range** | **Country** | **Diagnostic Methods** | **total (n)** | **single (%)** | **multiple (%)** | **total prev (%)** |
| --- | --- | --- | --- | --- | --- | --- | --- | --- | --- | --- |
| 87.5% | Sumbele et al., 2017 | Community | CS | 4-60 yrs | Cameroon | Kato-Katz | 450 | 11.8 | 2.2 | 14 |
| 87.5% | Madinga et al., 2017 | Community | CS | 1-80 yrs | DRC | Multiplex real-time PCR detection | 330 | 33 | 39.7 | 72.7 |
| 62.5% | Llewellyn et al., 2016 | Community | cRCT | UC | Timor Leste | Multiplex qPCR | 467 | 47.5 | 43.3 | 90.8 |
| 62.5% | Llewellyn et al., 2016 | Community | CS | 2-84 yrs | Cambodia | Multiplex qPCR | 213 | 30.5 | 8 | 38.5 |
| 62.5% | Wong et al., 2016 | School | CS | 7-9 yrs | Malaysia | Direct wet method, formal ether concentration, KK, Parasep | 33 | 24.2 | 72.7 | 97 |
| 100.0% | Worrell et al., 2016 | Community | CS | 0-14 yrs | Kenya | Kato-Katz | 676 | 30.7 | 9.5 | 40.2 |
| 87.5% | Gashaw et al., 2015 | School | CS | 5-17 yrs | Ethiopia | Kato-Katz thick smear | 550 | 45.6 | 20.6 | 66.2 |
| 75.0% | Gordon et al., 2015 | Community | CS | All | Philippines | Multiplex qPCR | 545 | 15.6 | 82.6 | 98.2 |
| 87.5% | Nwalorzie et al., 2015 | School | CS | 1-15 yrs | Nigeria | Kato-Katz, anal swabs | 220 | 27.7 | 45.5 | 73.2 |
| 50.0% | Hu et al., 2015 | Community | CS | 5-65 yrs | China | Kato-Katz | 1403 | 2.8 | 0.1 | 2.9 |
| 62.5% | Ferreira et al., 2015 | School | CS | 1-5 yrs | São Tomé and Príncipe | direct observation of stools in iodine and saline solutions after a formol-ether concentration procedure; Kato-Katz | 444 | 29.5 | 43.2 | 72.7 |
| 75.0% | Mejia Torres et al., 2014 | School | CS | 7-14 yrs | Honduras | Kato-Katz | 2554 | 28.9 | 14.6 | 43.5 |
| 87.5% | Vonghachack et al., 2014 | Community | CS | 2-95 yrs | Laos | Kato-Katz thick smear, Baermann technique | 729 | 23.6 | 65.3 | 88.9 |
| 62.5% | Lee et al., 2014 | Community | CS | 1-70 yrs | Malaysia | Formalin-ether concentration technique, acid-fast stain | 269 | 33.8 | 21.6 | 55.3 |
| 75.0% | Sayasone et al., 2014 | Community | CS | 0-12 yrs | Laos | Kato-Katz | 1313 | 36.3 | 40.4 | 76.7 |
| 75.0% | Matangila et al., 2014 | School | RCT (baseline) | 4-13 yrs | DRC | Kato-Katz | 457 | 26.7 | 12.5 | 39.2 |
| 87.5% | Sanchez et al., 2013 | School | CS | 7-14 yrs | Honduras | Kato-Katz | 320 | 40.3 | 32.2 | 72.5 |
| 87.5% | Ugbomoiko et al., 2012 | Community | CS | 3-17 yrs | Nigeria | Kato-Katz thick smear for stool, urine filtration | 419 | 31.7 | 46.5 | 78.3 |
| 100.0% | Coulibaly et al., 2012 | School | CS | 8-12 yrs | Cote d'Ivoire | Kato-Katz | 316 | 24.4 | 58.2 | 82.6 |
| 75.0% | Odiere et al., 2012 | School | CS | 5-19 yrs | Kenya | Kato-Katz | 4064 | 58 | 7.6 | 65.6 |
| 62.5% | Njenga et al., 2011 | Community | CS | 15-88 yrs | Kenya | Kato Katz, urine filtration, ICT test | 599 | 38.9 | 11.2 | 50.1 |
| 87.5% | Pilger et al., 2011 | Community | CS | 1-90 yrs | Brazil | Hoffman's sedimentation | 683 | 23.1 | 52.9 | 76 |
| 100.0% | Muller et al., 2011 | School | CS | 7-15 yrs | Cote d'Ivoire | Kato-Katz, urine filtration | 156 | 32.7 | 56.4 | 89.1 |
| 62.5% | Anah et al., 2008 | Community | CS | 1-5 yrs | Nigeria | Kato-Katz (1 stool) | 350 | 38 | 11.7 | 49.7 |
| 87.5% | Sousa-Figueiredo et al., 2008 | Community | CS | PSAC | Tanzania (Zanzibar) | Kato-Katz (1 stool) | 112 | 29.5 | 20.5 | 50 |
| 75.0% | Tengco et al., 2008 | Community | CS | 1-5 yrs | Philippines | Concentration technique | 1990 | 40.1 | 15.8 | 55.8 |
| 62.5% | Jardim-Botelho et al., 2008 | School | CS | 6-11 yrs | Brazil | Formalin-ether sedimentation technique | 196 | 26 | 66.8 | 92.9 |
| 87.5% | Ezeamama et al., 2008 | Community | CS (baseline) | 7-18 yrs | Philippines | Kato-Katz | 507 | 7.4 | 91.9 | 99.3 |
| 75.0% | Nkuo-Akenji et al, 2006 | Community | CS | 0-14 yrs | Cameroon | Kato-Katz | 425 | 24 | 14.4 | 38.4 |
| 62.5% | Fleming et al., 2006 | Community | CS | All | Brazil | Formalin-ether concentration technique, Kato-Katz | 1332 | 22.1 | 60.6 | 82.7 |
| 75.0% | Briand et al., 2005 | Community | CS (baseline) | 3-15 yrs | Senegal | Microscopic examination, urine filtration | 474 | 29.5 | 1.5 | 31 |
| 87.5% | Tchuem Tchuente et al., 2003 | School | CS | SAC | Cameroon | Kato Katz, urine filtration | 1044 | 27.5 | 62.7 | 90.2 |
| 62.5% | Thiong'o et al., 2001 | School | CS | 5-20 yrs | Kenya | Kato smears and urine filtration | 3158 | 38.6 | 29.2 | 67.8 |
| 75.0% | Brooker et al., 2000 | School | CS | 8-20 yrs | Kenya | Kato-Katz | 1738 | 26.6 | 65 | 91.6 |
| 87.5% | Scolari et al., 2000 | School | CS | 5-15 yrs | Brazil | Kato-Katz | 236 | 29.2 | 22.9 | 52.1 |
| 50.0% | Lili et al., 2000 | Community | CS | All | China | Kato-Katz | 766 | 39.4 | 35.8 | 75.2 |
| 75.0% | Widjana et al., 2000 | Community | CS | All | Indonesia | Kato-Katz, modified Harada Mori | 2394 | 28.8 | 58.2 | 87 |
| 62.5% | Toma et al., 1999 | Community | CS | All | Indonesia | modified Kato-Katz | 654 | 36.5 | 54.3 | 90.8 |
| 75.0% | Booth et al., 1998 | School | RCT (baseline CS) | 7-12 yrs | Tanzania | Kato-Katz | 1539 | 5.9 | 93.9 | 99.8 |
| 75.0% | Needham et al., 1998 | Community | CS | 1-88 yrs | Vietnam | Kato-Katz | 543 | 7.9 | 90.6 | 98.5 |
| 75.0% | Albonico et al., 1997 | School | CS | SAC | Tanzania (Zanzibar) | Kato-Katz | 3497 | 4.7 | 95.2 | 99.9 |
| 62.5% | Booth et al., 1996 | Community | CS | 5+ yrs | China | Kato-Katz | 1276 | 44.4 | 52.7 | 96.5 |
| 62.5% | Birrie et al., 1994 | Community | CS | All | Ethiopia | Ritchie's method | 1537 | 29 | 15.9 | 44.8 |
| 62.5% | Gbakima et al.,1994 | School | CS | 1-16 yrs | Sierra Leone | Kato thick smear and urine filtration | 1820 | 32.7 | 28.8 | 61.5 |
| 75.0% | Upatham et al., 1989 | Community | Baseline from longitudinal | All | Thailand | modified quick Kato smear | 1142 | 28.5 | 63.4 | 91.9 |
| 75.0% | Upatham et al., 1989 | School | Baseline from longitudinal | 6-12 yrs | Thailand | modified quick Kato smear | 518 | 24.1 | 72.6 | 96.7 |
| 62.5% | Holland et al., 1987 | Community | CS | 3-5 yrs | Panama | Ether concentration technique | 140 | 21.4 | 23.6 | 45 |
| 75.0% | Higgins et al., 1984 | Community | CS | All | Indonesia | McMaster, modified Kato, modified Harada Mori | 1387 | 30.1 | 46.4 | 76.6 |
| 62.5% | Ismid et al., 1981 | Community | CS | 3-20 yrs | Indonesia | modified Kato-Katz, modified Harada-Mori, anal swabs | 158 | 32.3 | 58.2 | 90.5 |
| 62.5% | Sinniah et al, 1978 | Community | CS | All | Malaysia | Sapero and Lawlers, examined by direct smear, brine flotation, formalin ether, and sedimentation techniques | 150 | 35.3 | 46 | 81.3 |
