# Supplementary material for: The extent, nature, and pathogenic consequences of helminth polyparasitism in humans: A meta-analysis
Source: PLoS Negl Trop Dis. 2019 Jun 18;13(6):e0007455. doi: 10.1371/journal.pntd.0007455 (PMC6599140; doi:10.1371/journal.pntd.0007455)
Supplement: S2 Table — (DOCX) [file pntd.0007455.s004.docx]

**S2 Table.** **Study characteristics of Type I and II helminth-protozoa studies included in the meta-analysis.** QA = Quality Assessment; CS = Cross-sectional; UC= unclear.

| **QA Score** | **Study Author and Publication Year** | **Study Population** | **Study Design** | **Age Range** | **Country** | **Diagnostic Methods** | **total (n)** | **Single**  **positive (%)** | **Multiple positive (%)** | **Total positive (%)** |
| --- | --- | --- | --- | --- | --- | --- | --- | --- | --- | --- |
| 75.0% | Chin et al., 2016 | Community | CS | 2-78 yrs | Malaysia | Formalin-ether microscopic method, PCR to identify species | 186 | 40.3 | 37.6 | 78 |
| 62.5% | Llewellyn et al., 2016 | Community | UC | UC | Timor Leste | Multiplex qPCR | 467 | 42 | 51.6 | 93.6 |
| 62.5% | Llewellyn et al., 2016 | Community | CS | 2-84 yrs | Cambodia | Multiplex qPCR | 213 | 36.6 | 11.3 | 47.9 |
| 62.5% | Al-Mekhlafi et al., 2016 | School | CS | 5-15 yrs | Yemen | Microscopic examination of direct saline and iodine wet preparations of stool samples, formol-ether sedimentation technique | 1218 | 34.7 | 9.5 | 44.2 |
| 75.0% | Muller et al., 2016 | School | CS | 9-12 yrs | South Africa | Kato-Katz, Hemastix , CCA, Crypto-Giardia Duo-Strip RDT, Pylori-Strip RDT | 934 | 24.2 | 16.9 | 41.1 |
| 87.5% | Furhimann et al., 2016 | Community | CS | 18+ yrs | Vietnam | Kato-Katz, formalin-ether concentration | 618 | 13.9 | 1.1 | 15 |
| 75.0% | Mekonnen et al., 2016 | Community | CS | 1-90 yrs | Ethiopia | Kato-Katz | 1021 | 39.7 | 12.4 | 52.1 |
| 75.0% | Dib et al., 2015 | Community | CS | 0-16 yrs | Argentina | Stool samples with wet mount (fresh and lugol) and 2 dyes for cryptosporidium, also used 2 concentration techniques: Faust (flotation) and Ritchie (sedimentation) | 115 | 22.6 | 55.7 | 78.3 |
| 87.5% | Sungkar et al., 2015 | Community | CS | 0-80 yrs | Indonesia | Direct-smear and microscopy | 424 | 19.6 | 76 | 95.6 |
| 62.5% | Macchioni et al., 2015 | Community | CS | 2-12 yrs | Bolivia | Microscopic examination in a drop of iodine of both wet smears and sediments after Ridley concentration | 268 | 31.3 | 37.7 | 69 |
| 75.0% | Bless et al., 2015 | School | CS | 5-18 yrs | Cambodia | Kato-Katz, formalin-ether concentration technique, qPCR | 228 | 36.4 | 33.8 | 70.2 |
| 62.5% | Ferreira et al., 2015 | School | CS | 1-5 yrs | São Tomé and Príncipe | Kato-Katz, direct observation of stools in iodine and saline solutions, formol-ether concentration procedure | 444 | 28.6 | 58.1 | 86.7 |
| 75.0% | Munoz-Antoli et al., 2014 | School | CS | 2-15 yrs | Nicaragua | Formol-ether, stained using modified Ziehl-Neelsen technique | 382 | 14.6 | 78.3 | 92.9 |
| 75.0% | Schar et al., 2014 | Community | CS | All | Cambodia | Kato-Katz, Koga Agar culture, Baermann, formalin-ether concentration technique, sodium nitrate and zinc sulfate flotations analysis | 218 | 29.4 | 58.2 | 87.6 |
| 87.5% | Al-Delaimy et al., 2014 | School | CS | 6-12 yrs | Malaysia | Direct smear, formalin-ether sedimentation, Kato-Katz, harada Mori, trichome stain, and modified Ziehl Neelsen | 498 | 28.1 | 70.3 | 98.4 |
| 62.5% | Ahmad et al., 2014 | Both | CS | 1-80 yrs | Malaysia | Formalin-ether concentration technique, stained with iodine, Ziehl-Neelsen staining | 131 | 8.4 | 1.5 | 9.9 |
| 50.0% | Boonjaraspinyo et al., 2013 | Community | CS | 2-80 yrs | Thailand | Formalin-ether concentration | 253 | 31.2 | 5.9 | 37.1 |
| 75.0% | Verhagen et al., 2013 | Community | CS | 4-16 yrs | Venezuela | Wet mount examinations, Kato-Katz, Baermann, high pure PCR | 390 | 31.3 | 36.4 | 67.7 |
| 62.5% | Wassie et al., 2013 | School | CS | 12-20 yrs | Ethiopia | Direct stool microscopy, formalin ether concentration technique | 245 | 17.6 | 2.4 | 20 |
| 100.0% | Coulibaly et al., 2012 | School | CS | 8-12yrs | Cote d'Ivoire | Kato-Katz | 446 | 20.9 | 76 | 96.9 |
| 87.5% | Goncalves et al., 2011 | School | CS | 0-6 yrs | Brazil | Lutz method | 133 | 22.6 | 6.8 | 29.3 |
| 87.5% | Traore et al., 2011 | School | CS | 6-15 yrs | Cote d'Ivoire | Kato-Katz, ether-concentration technique | 166 | 17.5 | 17.5 | 35 |
| 87.5% | Matthys et al., 2011 | School | CS | 7-11 yrs | Tajikistan | Kato-Katz | 594 | 40.9 | 22.2 | 63.1 |
| 75.0% | Hamm et al., 2009 | School | CS baseline | SAC | Togo | Kato-Katz, urine filtration | 729 | 32 | 54 | 86 |
| 62.5% | Korkes et al., 2008 | Community | CS | 2-14 yrs | Brazil | Direct exam, Kato-Katz, Lutz-Hoffman, thermal migration method, Zinc sulfate flotation | 120 | 20 | 10.8 | 30.8 |
| 62.5% | Nematian et al., 2008 | School | CS | SAC | Iran | Microscopic examination of fecal sample, adhesive-tape samples | 19209 | 16.4 | 2 | 18.4 |
| 87.5% | Quihui-Cota et al., 2004 | School | CS | 6-10 yrs | Mexico | Faust technique, Kato Katz | 400 | 14.8 | 62 | 76.8 |
| 75.0% | Keiser et al., 2002 | Community | CS | All | Cote d'Ivoire | Kato-Katz | 260 | 9.2 | 87.3 | 96.5 |
| 75.0% | Guignard et al., 2000 | Community | CS | PSAC+  SAC | Argentina | Teleman method, Kinyoun's, Logal, and trichomic staining direct immunofluorescence, flotation method | 396 | 21.5 | 63.4 | 84.8 |
| 62.5% | Al-Agha et al., 2000 | School | CS | 6-11 yrs | Palestine | Concentration techniques: zinc sulfate centrifugal flotation, formol-ether sedimentation | 209 | 36.8 | 6.2 | 43.1 |
| 87.5% | Saldiva et al., 1999 | Community | CS | 1-12 yrs | Brazil | Hoffman technique | 520 | 23.7 | 55.4 | 79 |
| 75.0% | Gamboa et al., 1998 | Community | CS | <14 yrs | Argentina | Carles-Barthelemy sedimentation and Willis flotation technique | 292 | 32.9 | 21.9 | 54.8 |
| 75.0% | Kang et al., 1998 | Community | CS | 0-65 yrs | India | Saline/iodine prep for formol-ether concentration, smear with safranine-methylene blue stain | 78 | 23.1 | 74.4 | 97.4 |
| 62.5% | Chunge et al., 1995 | Community | CS | All | Kenya | Formol-ether concentration method, direct saline smear | 1011 | 16.2 | 67.1 | 83.3 |
| 75.0% | Ferreira et al., 1994 | Community | CS | All | Brazil | Hoffman sedimentation, Kato-Katz for positive AL and TT | 407 | 30.5 | 15.2 | 45.7 |
| 62.5% | Chunge et al., 1991 | Community | CS | All | Kenya | Direct-smear and modified formol-ether concentration | 1129 | 22.1 | 59.1 | 81.2 |
| 62.5% | Holland et al., 1987 | Community | CS | 3-5 yrs | Panama | Ether concentration technique | 140 | 24.3 | 29.3 | 53.6 |
| 62.5% | Annan et al., 1986 | Community | CS | 1-5 yrs | Ghana | Microscopic examination of stool fixed in 10% formaldehyde | 422 | 30.8 | 39.3 | 70.1 |
| 50.0% | Carney et al., 1974 | Community | CS | All | Indonesia | Direct and formalin-ether concentration methods | 542 | 41 | 36 | 77 |
| 50.0% | Carney et al., 1974b | Community | CS | 1-88 yrs | Indonesia | Direct and formalin-ether concentration methods | 671 | 26 | 66 | 92 |
