# Supplementary material for: The extent, nature, and pathogenic consequences of helminth polyparasitism in humans: A meta-analysis
Source: PLoS Negl Trop Dis. 2019 Jun 18;13(6):e0007455. doi: 10.1371/journal.pntd.0007455 (PMC6599140; doi:10.1371/journal.pntd.0007455)
Supplement: S3 Table — (DOCX) [file pntd.0007455.s005.docx]

**S3 Table.** **Study characteristics of Type I helminth-malaria studies included in the meta-analysis.** QA = Quality Assessment; CS = Cross-sectional.

| **QA Score** | **Study Author and Publication Year** | **Study Population** | **Study Design** | **Age Range** | **Country** | **# helminth. spp. found** | **Helminth Diagnostic** | **Malaria Diagnostic** | **total (n)** | **M+/H-**  **(single)**  **(%)** | **M+/H+**  **(multiple) (%)** |
| --- | --- | --- | --- | --- | --- | --- | --- | --- | --- | --- | --- |
| 77.8% | Sumbele et al., 2017 | Community | CS | 4-60 yrs | Cameroon | 2 | Kato-Katz | Giemsa blood smear | 450 | 27.8 | 5.6 |
| 55.6% | Njua-Yafi et al., 2016 | Community | CS (baseline) | 0-10 yrs | Cameroon | 6 | Formol-ether sedimentation | Venous blood sample for malaria parasitaemia | 318 | 15.7 | 3.5 |
| 66.7% | Burdam et al., 2016 | Community | CS | <5 yrs | Indonesia | 3 | Kato Katz | Peripheral asexual parasitaemia by microscopy | 269 | 5.9 | 7.1 |
| 66.7% | Adedoja et al., 2015 | School | CS | 4-15 yrs | Nigeria | 6 | Formol-ether, Kato-Katz, urine sedimentation | Venous blood samples and thick blood films | 1017 | 6.2 | 14.4 |
| 66.7% | Salim et al., 2015 | Community | CS | 0-9 yrs | Tanzania | 5 | Kato-Katz, ICT | Giemsa-stained thick and thin blood smears | 992 | 8.1 | 5 |
| 77.8% | Kepha et al., 2015 | School | CS | 5-18 yrs | Kenya | 3 | Kato-Katz | Giemsa-stained thick and think smears | 5471 | 32.2 | 14.3 |
| 55.6% | Zeukeng et al., 2014 | Community | CS | All | Cameroon | 2 | Kato-Katz |  | 263 | 57.8 | 19.4 |
| 77.8% | Yapi et al., 2014 | School | CS | 5-16 yrs | Cote d'Ivoire | 3 | Kato-Katz | Blood films for plasmodium | 5104 | 49.8 | 13.5 |
| 66.7% | Kinung'hi et al., 2014 | School | CS | 3-13 yrs | Tanzania | 4 | Kato-Katz | Thick blood smear | 1546 | 11.9 | 17.9 |
| 66.7% | Sanchez-Arcila et al., 2014 | Community | CS | All | Brazil | 4 | Direct wet mount microscopic and concentration techniques | Thick blood smear and/or PCR | 264 | 20.8 | 3.4 |
| 88.9% | Muller et al., 2011 | School | CS | 7-15 yrs | Cote d'Ivoire | 4 | Kato-Katz, urine filtration | RDT | 156 | 7.1 | 64.1 |
| 77.8% | Midzi et al., 2010 | Community | CS (baseline) | 6-17 yrs | Zimbabwe | 5 | Kato-Katz and sedimentation, urine filtration | Giemsa-stained thick blood smears | 491 | 8.1 | 18.5 |
| 66.7% | Nkuo-Akenji et al, 2006 | Community | CS | 0-14 yrs | Cameroon | 3 | Kato-Katz | Giemsa-stained thick blood smear | 425 | 39.8 | 24.7 |
| 55.6% | Adio et al., 2004 | School | CS | 4-15 yrs | Cameroon | 3 | Kato-Katz | Giemsa-stained thick and thin blood smears | 243 | 61.7 | 38.3 |
| 55.6% | van den Biggelaar et al., 2001 | School | CS | 5-15 yrs | Gabon | 3 | Urine filtration; Blood screened for microfilariae | Giemsa-stained thick blood smears | 520 | 22.7 | 20 |
