# Supplementary material for: The extent, nature, and pathogenic consequences of helminth polyparasitism in humans: A meta-analysis
Source: PLoS Negl Trop Dis. 2019 Jun 18;13(6):e0007455. doi: 10.1371/journal.pntd.0007455 (PMC6599140; doi:10.1371/journal.pntd.0007455)
Supplement: S4 Table — (DOCX) [file pntd.0007455.s006.docx]

**S4 Table.** **Study characteristics of Type I helminth-tuberculosis (TB) studies included in the meta-analysis.** QA = Quality Assessment; CS = Cross-sectional; CC = Case-Control; UC= unclear.

| **QA Score** | **Study Author and Publication Year** | **Study Populations** | **Study Design** | **Age Range** | **Country** | **Helminth Diagnostic** | **TB Diagnostic** | **total (n)** | **single (%)** | **multiple (%)** |
| --- | --- | --- | --- | --- | --- | --- | --- | --- | --- | --- |
| 62.5% | Alemu et al., 2017 | PTB patients from TB clinics | CS | 15-65 yrs | Ethiopia | Direct saline and/or formol-ether concentration technique | PTB based on national diagnostic guideline: persistent (>2 weeks) cough, 2 sputum exams with + AFB or 1 sputum exam is + and chest x-ray suggestive for PTB | 213 | 75.6 | 24.4 |
| 70.0% | Mhimbira et al., 2017 | TB patients from TB clinics and hospital | CC | ≥ 18 yrs | Tanzania | Kato-Katz, Baermann method, FLOTAC, adhesive tape test, POC-CCA, urine filtration | Sputum-smear positives for acid-fast bacilli | 597 | 68.2 | 31.8 |
| 87.5% | Li et al., 2015 | PTB patients without HIV undergoing anti-TB treatment | CS | All | China | Modified Kato-Katz | UC | 369 | 92.4 | 7.6 |
| 60.0% | Abate et al., 2015 | TB patients without HIV | CC | 15-60 yrs | Ethiopia | Direct microscopy and Kato-Katz | At least 1 sputum sample + for AFB in the presence of clinical symptoms for active TB) OR clinical symptoms suggestive of TB with 3 - sputum smears, radiographic abnormalities consistent with PTB and no response to 1-week broad spectrum antibiotic therapy | 424 | 63.2 | 36.8 |
| 88.9% | Biraro et al., 2014 | Newly diagnosed sputum smear+ TB patients | Cohort | > 18 yrs | Uganda | Kato-Katz on 3 consecutive daily stool samples | QFN blood test | 146 | 88.4 | 11.6 |
| 66.7% | Chatterjee et al., 2014 | Active PTB-positive identified in community CS study | CS (baseline) | 6-65 yrs | India | Direct microscopy and formal-gasoline concentration techniques | Pulmonary symptoms; smear positive and/or culture positive for Mtb | 25 | 52 | 48 |
| 88.9% | Perez-Porcuna et al., 2014 | Children 0-6 in contact with TB+ adult | CS | 0-6 yrs | Brazil | Spontaneous sedimentation method | QFT blood test | 28 | 78.6 | 21.4 |
| 60.0% | Abate et al., 2012 | Consecutive smear + TB patients | . | 15-65 yrs | Ethiopia | Direct and Kato-Katz technique | Smear + TB (at least 2 sputum smears AFB+ or 1 smear+ slide & x-ray results suggestive of TB) | 112 | 71.4 | 28.6 |
| 75.0% | Kassu et al., 2007 | TB patients in teaching hospital | CS | 15-80 yrs | Ethiopia | Direct and formalin-ether concentration methods | Standard protocol w/clinical, radiological, and histopathological and lab features of patients | 257 | 55.6 | 44.4 |
| 60.0% | Elias et al., 2006 | Smear+ TB patients diagnosed at outpatient dept of teaching hospital; | CC | > 10 yrs | Ethiopia | Direct microscopy and formol-ether concentration | 2/3 morning sputum samples + for AFB; Sputum smear microscopy using sodium hypochlorite concentration technique | 230 | 29.1 | 70.9 |
| 66.7% | Ramos et al., 2006 | Admitted adult hospital patients | Retrospective chart review | Adults | Ethiopia | Stool: unknown | unclear: hospitalized TB patients | 100 | 84 | 16 |
| 75.0% | Resende Co et al., 2006 | Newly diagnosed PTB+, HIV- patients at clinic | CS (baseline) | Adults | Brazil | Lutz, Kato-Katz, Baerman-Moraes | AFB smear-positive | 40 | 72.5 | 27.5 |
| 60.0% | Tristao-Sa et al., 2002 | PTB+ hospitalized patients | CC | Adults | Brazil | Lutz-Hoffman method | UC | 57 | 42.1 | 57.9 |
