# Supplementary material for: The extent, nature, and pathogenic consequences of helminth polyparasitism in humans: A meta-analysis
Source: PLoS Negl Trop Dis. 2019 Jun 18;13(6):e0007455. doi: 10.1371/journal.pntd.0007455 (PMC6599140; doi:10.1371/journal.pntd.0007455)
Supplement: S5 Table — (DOCX) [file pntd.0007455.s007.docx]

**S5 Table. Study characteristics of Type I helminth-HIV studies included in the meta-analysis.** QA = Quality Assessment; CS = Cross-sectional; CC = Case-Control; UC= unclear.

| **QA** | **Study Author and Publication Year** | **Study Population** | **Study Design** | **Age Range** | **# Helminths** | **Country** | **Helminth Diagnostic** | **HIV Diagnostic** | **total (n)** | **single (%)** | **multiple (%)** |
| --- | --- | --- | --- | --- | --- | --- | --- | --- | --- | --- | --- |
| 87.5% | Adeleke et al., 2015 | HIV+ and HAART-naïve patients at outpatient dept of hospital | CS | 18-64 yrs | 7 | South Africa | Formalin ethyl acetate concentration method | Diagnosed at clinic; CD4 count done using PLG methodology and viral load using COBA AmpliPrep HIVE-1 test v2.0 | 231 | 75.3 | 24.7 |
| 66.7% | Oyedeji et al., 2015 | HIV+ children | CS | 0-14 yrs | 2 | Nigeria | Direct method and formal ether concentration technique, modified Ziehl-Neelsen | Positive ELISA and w. blot confirmation | 52 | 98.1 | 1.9 |
| 87.5% | Janssen et al., 2015 | HIV+ patients from clinic (either ART naïve or taking ART for at least 3 months) | CS | >18 yrs | 7 | Gabon | Kato-Katz, modified agar-plate culture technique | UC | 252 | 69.4 | 30.6 |
| 75.0% | Paboriboune et al., 2014 | Admitted patients for care of newly diagnosed HIV infection; ART- naïve | CS | All | 6 | Laos | Kato thick smear, formalin-ethyl concentration technique, modified acid-fast staining | Serological diagnosis using Determine HIV 1/2 and Uni-Gold HIV and ELISA if discordant results | 137 | 41.6 | 58.4 |
| 55.6% | Taye et al., 2014 | HIV+ patients visiting lab for CD4 T-cell count | Comparative CS | All | 7 | Ethiopia | Direct wet mount, formol ether concentration, modified Ziehl-Neelson | UC | 316 | 88 | 12 |
| 75.0% | Efraim et al., 2014 | HIV clinic infected adults who had taken ART for 6-15 months | Retrospective cohort | Adults | 2 | Tanzania | CCA | UC | 351 | 72.4 | 27.6 |
| 75.0% | Arndt et al., 2013 | HIV+ clinic patients who did not meet WHO criteria for ART initiation | CS (nested in RCT) | ≥ 18 yrs | 5 | Kenya | PCR | HIV seropositive | 153 | 64.1 | 35.9 |
| 80.0% | Roka et al., 2013 | HIV+ individuals attending health center | CC | 1-65 yrs | 6 | Equatorial Guinea | Microscopic analysis w/ saline and iodine, formaldehyde-ether concentrations, Ziehl-Neelsen smear | HIV seropositive | 273 | 41.8 | 58.2 |
| 60.0% | Roka et al., 2012 | HIV+ who came to Infectious Diseases Reference Units for HIV check-up | CS | 1-63 yrs | 5 | Equatorial Guinea | Formol-ether method, Ziehl-Neelsen, IC assay, | UC | 260 | 37.7 | 62.3 |
| 70.0% | Tian et al., 2012 | HIV + from local clinic; | CC | 6-65 yrs | 4 | China | Kato-Katz | Screened for anti-HIV antibodies by ELISA and confirmation by western blot immunoassay | 302 | 95.7 | 4.3 |
| 62.5% | Wumba et al., 2012 | HIV/AIDS patients from 4 main hospitals | CS | 15-73 yrs | 2 | DRC | Microscopic examination of feces and direct concentration method of Ritchie protocol | UC | 242 | 97.2 | 2.8 |
| 50.0% | Asma et al., 2011 | HIV patients from hospitals | CS | 1-42 yrs | 3 | Malaysia | Formol-ether sedimentation technique | UC | 346 | 80.9 | 19.1 |
| 60.0% | Mkhize-Kwitshana et al., 2011 | HIV+ from support group at clinic | CS | > 18 yrs | 5 | South Africa | Formol-ether and Kato-Katz | Confirmed by rapid test for HIV, serum retested for antibody detection confirmed by PCR sequencing of viral DNA | 124 | 58.9 | 41.1 |
| 62.5% | Idindili et al., 2011 | HIV+ adults registering for first time at HIV/AIDS management clinics | CS (baseline) | > 18 yrs | 4 | Tanzania | Formol-ether concentration techniques, quantified by Kato-Katz | Clinicians staged HIV status according to WHO and Tanzanian clinical guidelines | 421 | 64.8 | 35.2 |
| 66.7% | Mwambete et al., 2010 | Hospital HIV+ inpatients | CS | 8-76 yrs | 4 | Tanzania | Macroscopic and microscopic examinations | UC | 66 | 81.8 | 18.2 |
| 55.6% | Walson et al., 2010 | HIV=infected adult who were ART-naïve and didn't meet WHO criteria for HAART initation | CS (baseline) | ≥ 18 yrs | 5 | Kenya | Wet prep, Kato-Katz, Formol-ether concentration technique | HIV seropositive | 1541 | 80.7 | 19.3 |
| 55.6% | Hosseinipour et al., 2007 | Individuals attending HIV counseling and testing services or outpatient clinics at 1 hospital | CS (baseline) | Adults | 6 | Malawi | Direct smear of formalin-fixed stool and concentrated smear using ethyl acetate sedimentation | 2 rapid HIV tests (Determine and UniGold: considered + if both were +; HIV ELISA on discordant results) | 266 | 82.7 | 17.3 |
| 60.0% | Da Silva et al., 2005 | HIV/AIDS patients at hospital | CC | 18-76 yrs | 5 | Brazil | Baermann, Lutz, Ritchie methods, Safranin/Methylene Blue, Eber's chromotrope techniques | ELISA and MEIA methods | 100 | 82 | 18 |
| 75.0% | Modjarrad et al., 2005 | HIV+ but asymptomatic for HIV disease | CS | Adults | 6 | Zambia | Formol-ether concentration, Kato-Katz if ova or larvae found | Positive for dual rapid test algorithm | 297 | 75.1 | 24.9 |
| 66.7% | Kallestrup et al., 2005 | HIV+ identified in community CS survey | CS | ≥ 18 yrs | 2 | Zimbabwe | Modified formol-ether concentration technique, urine filtration | Rapid HIV-1/2 test kit on dry blood spot, different rapid test kit ig +, ELISAs for those included in cohort | 407 | 54.5 | 45.5 |
| 75.0% | Brown et al., 2004 | HIV+ patients attending AIDS support organization or Uganda Virus Research Institute | Cohort study (baseline) | Adults | 7 | Uganda | Kato-Katz, modified formol-ether, charcoal cultures | UC | 547 | 46.2 | 53.8 |
| 62.5% | Singh et al., 2004 | HIV+ Patients admitted to hospital or de-addiction centers | CC | All | 4 | India | Normal saline method, flotation method and concentration technique, Baerman modified funnel technique for SS | HIV+ ELISA/Simple/Rapid test | 147 | 30.6 | 69.4 |
| 75.0% | Brown et al., 2003 | Patients attending clinics at AIDS clinic or research institute in Uganda | Cohort study (baseline) | Adult | 5 | Uganda | Kato-Katz, formol-ether concentration, charcoal culture, CCA | UC | 412 | 51 | 49 |
