# Supplementary material for: The extent, nature, and pathogenic consequences of helminth polyparasitism in humans: A meta-analysis
Source: PLoS Negl Trop Dis. 2019 Jun 18;13(6):e0007455. doi: 10.1371/journal.pntd.0007455 (PMC6599140; doi:10.1371/journal.pntd.0007455)
Supplement: S6 Table — (DOCX) [file pntd.0007455.s008.docx]

**S6 Table.** **Quality assessment scores for each study considered in this meta-analysis using the NIH Quality Assessment Tool for Observational Cohort and Cross-sectional Studies.** HH = Helminth-helminth; HP = Helminth-intestinal protozoa; HM = Helminth-malaria; HTB = Helminth-tuberculosis; HHIV = Helminth-HIV; T1 = Type 1 (prevalence difference); T2 = Type 2 (species density distribution model); T3 = Type 3 (association); Y = Yes; N= No; NR = Not reported; UC = Unclear; NA = Not applicable. Questions corresponding to the number listed in the table are presented here:

1.Was the research question or objective in this paper clearly stated?

2. Was the study population clearly specified and defined?

3. Was the participation rate of eligible persons at least 50%?

4. Were all the subjects selected or recruited from the same or similar populations (including the same time period)? Were inclusion and exclusion criteria for being in the study prespecified and applied uniformly to all participants?

5. Was a sample size justification, power description, or variance and effect estimates provided?

6. For the analyses in this paper, were the exposure(s) of interest measured prior to the outcome(s) being measured?

7. Was the timeframe sufficient so that one could reasonably expect to see an association between exposure and outcome if it existed?

8. For exposures that can vary in amount or level, did the study examine different levels of the exposure as related to the outcome (e.g., categories of exposure, or exposure measured as continuous variable)?

9. Were the exposure measures (independent variables) clearly defined, valid, reliable, and implemented consistently across all study participants?

10. Was the exposure(s) assessed more than once over time?

11. Were the outcome measures (dependent variables) clearly defined, valid, reliable, and implemented consistently across all study participants?

12. Were the outcome assessors blinded to the exposure status of participants?

13. Was loss to follow-up after baseline 20% or less?

14. Were key potential confounding variables measured and adjusted statistically for their impact on the relationship between exposure(s) and outcome(s)

| **Study Author and Publication Year** | **Data Type** | **Data Pair** | **Question Number** | | | | | | | | | | | | | | **Quality Assessment Scores** | | | |
| --- | --- | --- | --- | --- | --- | --- | --- | --- | --- | --- | --- | --- | --- | --- | --- | --- | --- | --- | --- | --- |
|  |  |  | **1** | **2** | **3** | **4** | **5** | **6** | **7** | **8** | **9** | **10** | **11** | **12** | **13** | **14** | **T1/T2 HH, HP** | **T1 HM, HTB, HHIV** | **T3 HH, HP** | **T3 HM, HTB, HHIV** |
| Sumbele et al., 2017 | 1,2,3 | HH, HM | Y | Y | Y | Y | Y | N | N | NA | Y | N | Y | NA/NR(M) | NA | N(3) | 87.5% | 77.8% | 63.6% | 58.3% |
| Madinga et al., 2017 | 1,3 | HH | Y | Y | Y | Y | Y | N | N | NA | Y | N | Y | NA | NA | Y(3) | 87.5% |  | 72.7% |  |
| Alemu et al., 2017 | 1 | HTB | Y | Y | NR | Y | N | N | N | NA | Y | N | Y | NA | NA | NA |  | 62.5% |  |  |
| Llewellyn et al., 2016 (Timor Leste) | 1 | HH, HP | Y | Y | NR | NR | Y | NR | NR | NR | Y | N | Y | NA | NR | NA | 62.5% |  |  |  |
| Llewellyn et al., 2016 (Cambodia) | 1 | HH, HP | Y | Y | NR | Y | N | N | N | NA | Y | N | Y | NA | NA | NA | 62.5% |  |  |  |
| Wong et al., 2016 | 1,2,3 | HH | Y | Y | NR | Y | N | N | N | NA | Y | N | Y | NA | NA | N(3) | 62.5% |  | 45.5% |  |
| Worrell et al., 2016 | 1,2,3 | HH | Y | Y | Y | Y | Y | N | N | NA | Y | Y | Y | NA | NA | N(3) | 100.0% |  | 72.7% |  |
| Chin et al., 2016 | 1,2,3 | HP, HH(3) | Y | Y | NR | Y | Y | N | N | NA | Y | N | Y | NA | NA | N(3) | 75.0% |  | 54.5% |  |
| Al-Mekhlafi et al., 2016 | 1,2 | HP | Y | Y | NR | Y | N | N | N | NA | Y | N | Y | NA | NA | NA | 62.5% |  |  |  |
| Muller et al., 2016 | 1,3 | HP (1), HH (3) | Y | Y | Y | Y | N | N | N | NA | Y | N | Y | NA | NA | N(3) | 75.0% |  | 54.5% |  |
| Furhimann et al., 2016 | 1 | HP | Y | Y | Y | Y | Y | N | N | NA | Y | N | Y | NA | NA | NA | 87.5% |  |  |  |
| Mekonnen et al., 2016 | 1,2,3 | HP, HH(3) | Y | Y | NR | Y | Y | N | N | NA | Y | N | Y | NA | NA | N(3) | 75.0% |  | 54.5% |  |
| Njua-Yafi et al., 2016 | 1 | HM | Y | Y | NR | Y | N | N | N | NA | Y | N | Y | NR | NA | NA |  | 55.6% |  |  |
| Burdam et al., 2016 | 1 | HM | Y | Y | N | Y | Y | N | N | NA | Y | N | Y | NR | NA | NA |  | 66.7% |  |  |
| Morenikeji et al., 2016 | 3 | HM | Y | Y | Y | Y | Y | N | N | NA | Y | N | Y | NR | NA | N(3) |  |  |  | 58.3% |
| Drame et al., 2016 | 3 | HH, HM | Y | Y | NR | Y | N | N | N | NA | Y | N | Y | NA/NR(M) | NA | N(3) |  |  | 45.5% | 41.7% |
| Kuong et al., 2016 | 3 | HH | Y | Y | Y | Y | N | N | N | NA | Y | N | Y | NA | NA | N(3) |  |  | 54.5% |  |
| Jeong et al., 2016 | 3 | HH | Y | Y | NR | Y | N | N | N | NA | Y | N | Y | NA | NA | N(3) |  |  | 45.5% |  |
| Kroidl et al., 2016 | 3 | HHIV | Y | Y | Y | Y | N | N | N | NA | Y | N | Y | Yes | NA | Y(3) |  |  |  | 66.7% |
| Kroidl et al., 2016 | 3 | HHIV | Y | Y | Y | Y | N | Y | Y | NA | Y | Y | Y | Yes | No | Y(3) |  |  |  | 84.6% |
| Ayeh-Kumi et al., 2016 | 3 | HM | Y | Y | Y | Y | N | N | N | NA | Y | N | Y | NR | NA | N(3) |  |  |  | 50.0% |
| Becker et al., 2015 | 3 | HH | Y | Y | Y | Y | N | N | N | NA | Y | N | Y | NA | NA | N(3) |  |  | 54.5% |  |
| Ferreira et al., 2015 | 1,2,3 | HH, HP | Y | Y | NR | Y | N | N | N | NA | Y | N | Y | NA | NA | N(3) | 62.5% |  | 45.5% |  |
| Gashaw et al., 2015 | 1,2,3 | HH | Y | Y | Y | Y | Y | N | N | NA | Y | N | Y | NA | NA | N(3) | 87.5% |  | 63.6% |  |
| Xiao et al., 2015 | 3 | HH | Y | Y | Y | Y | N | N | N | NA | Y | N | Y | NA | NA | Y(3) |  |  | 63.6% |  |
| Gordon et a., 2015 | 1,2,3 | HH | Y | Y | NR | Y | Y | N | N | NA | Y | N | Y | NA | NA | N(3) | 75.0% |  | 54.5% |  |
| Nwalorzie et al., 2015 | 1 | HH | Y | Y | Y | Y | N | N | N | NA | Y | Y | Y | NA | NA | NA | 87.5% |  |  |  |
| Hu et al., 2015 | 1,2,3 | HH | Y | N | NR | Y | N | N | N | NA | Y | N | Y | NA | NA | N(3) | 50.0% |  | 36.4% |  |
| Bless et al., 2015 | 1,2 | HP | Y | Y | NR | Y | N | N | N | NA | Y | Y | Y | NA | NA | NA | 75.0% |  |  |  |
| Dib et al., 2015 | 1 | HP | Y | Y | Y | Y | N | N | N | NA | Y | N | Y | NA | NA | NA | 75.0% |  |  |  |
| Sungkar et al., 2015 | 1 | HP | Y | Y | Y | Y | Y | N | N | NA | Y | N | Y | NA | NA | NA | 87.5% |  |  |  |
| Macchioni et al. ,2015 | 1 | HP | Y | Y | NR | Y | N | N | N | NA | Y | N | Y | NA | NA | NA | 62.5% |  |  |  |
| Kepha et al., 2015 | 1,3 | HM,HH(3) | Y | Y | Y | Y | Y | N | N | NA | Y | N | Y | NA/NR(M) | NA | Y(3) |  | 77.8% | 72.7% | 66.7% |
| Salim et al., 2015 | 1,3 | HM | Y | Y | NR | Y | Y | N | N | NA | Y | N | Y | NR | NA | N(3) |  | 66.7% |  | 50.0% |
| Adedoja et al., 2015 | 1,3 | HM | Y | Y | NR | Y | N | N | N | NA | Y | Y | Y | NR | NA | N(3) |  | 66.7% | 54.5% | 50.0% |
| Janssen et al., 2015 | 1 | HHIV | Y | Y | NR | Y | Y | N | N | NA | Y | Y | Y | NA | NA | NA |  | 87.5% |  |  |
| Adeleke et al., 2015 | 1 | HHIV | Y | Y | Y | Y | Y | N | N | NA | Y | N | Y | NA | NA | NA |  | 87.5% |  |  |
| Oyedeji et al., 2015 | 1 | HHIV | Y | Y | Y | Y | N | N | N | NA | Y | N | Y | NR | NA | NA |  | 66.7% |  |  |
| Li et al., 2015 | 1 | HTB | Y | Y | Y | Y | N | N | N | NA | Y | Y | Y | NA | NA | NA |  | 87.5% |  |  |
| Sanya et al., 2015 | 3 | HHIV | Y | Y | NR | Y | Y | N | N | NA | Y | N | Y | NR | NA | Y(3) |  |  |  | 58.3% |
| Tafatatha et al., 2015 (study 1) | 3 | HHIV | Y | Y | NR | Y | N | N | N | NA | Y | N | Y | NR | NA | Y(3) |  |  |  | 50.0% |
| Tafatatha et al., 2015 (study 2) | 3 | HHIV | Y | Y | Y | Y | N | N | N | NA | Y | N | Y | NR | NA | Y(3) |  |  |  | 58.3% |
| Yapi et al., 2014 | 1,3 | HM,HH(3) | Y | Y | Y | Y | Y | N | N | NA | Y | N | Y | NA/NR(M) | NA | Y(3) |  | 77.8% | 72.7% | 66.7% |
| Matangila et al., 2014 | 1 | HH | Y | Y | NR | Y | Y | N | N | NA | Y | N | Y | NA | NA | NA | 75.0% |  |  |  |
| Kinung'hi et al., 2014 | 1,3 | HM | Y | Y | NR | Y | N | N | N | NA | Y | Y | Y | NR | NA | N(3) |  | 66.7% |  | 50.0% |
| Sanchez-Arcila et al., 2014 | 1 | HM | Y | Y | NR | Y | Y | N | N | NA | Y | N | Y | NR | NA | NA |  | 66.7% |  |  |
| Munoz-Antoli et al., 2014 | 1,2 | HP | Y | Y | NR | Y | Y | N | N | NA | Y | N | Y | NA | NA | N(3) | 75.0% |  |  |  |
| Mejia Torres et al., 2014 | 1 | HH | Y | Y | NR | Y | Y | N | N | NA | Y | N | Y | NA | NA | NA | 75.0% |  |  |  |
| Sayasone et al., 2014 | 1 | HH | Y | Y | Y | Y | N | N | N | NA | Y | N | Y | NA | NA | NA | 75.0% |  |  |  |
| Ranjitkar et al., 2014 | 3 | HH | Y | N | NR | Y | N | N | N | NA | Y | N | Y | NA | NA | N(3) |  |  | 36.4% |  |
| Zeukeng et al., 2014 | 1,3 | HM,HH(3) | Y | Y | NR | Y | N | N | N | NA | Y | N | Y | NR | NA | N(3) | 62.5% | 55.6% | 36.4% | 33.3% |
| Vonghachack et al., 2014 | 1,2 | HH | Y | Y | Y | Y | N | N | N | NA | Y | Y | Y | NA | NA | NA | 87.5% |  |  |  |
| Lee et al., 2014 | 1 | HH | Y | Y | NR | Y | N | N | N | NA | Y | N | Y | NA | NA | NA | 62.5% |  |  |  |
| Al-Delaimy et al., 2014 | 1,2 | HP | Y | Y | Y | Y | Y | N | N | NA | Y | N | Y | NA | NA | NA | 87.5% |  |  |  |
| Schar et al., 2014 | 1,2 | HP | Y | Y | NR | Y | N | N | N | NA | Y | Y | Y | NA | NA | NA | 75.0% |  |  |  |
| Ahmad et al., 2014 | 1,2 | HP | Y | N | N | Y | Y | N | N | NA | Y | N | Y | NA | NA | NA | 62.5% |  |  |  |
| Salim et al., 2014 | 3 | HH, HM | Y | Y | NR | Y | Y | N | N | NA | Y | N | Y | NA/NR(M) | NA | Y(3) |  |  | 63.6% | 58.3% |
| Hurlimann et al, 2014b | 3 | HM | Y | Y | NR | Y | Y | N | N | NA | Y | N | Y | NR | NA | N(3) |  |  |  | 50.0% |
| Hurlimann et al, 2014 | 3 | HH, HP, HM | Y | Y | Y | Y | N | N | N | NA | Y | N | Y | NA/NR(M) | NA | Y(3) |  |  | 63.6% | 58.3% |
| Bragagnoli et al, 2014 | 3 | HH, HP | Y | Y | Y | Y | N | N | N | NA | Y | N | Y | NA | NA | N(3) |  |  | 54.5% |  |
| Mathewos et al., 2014 | 3 | HH | Y | Y | Y | Y | Y | N | N | NA | Y | N | Y | NA | NA | N(3) |  |  | 63.6% |  |
| Casmo et al., 2014 | 3 | HH | Y | Y | NR | Y | N | N | N | NA | Y | N | Y | NA | NA | N(3) |  |  | 45.5% |  |
| Mazigo et al., 2014 | 1,3 | HHIV | Y | Y | NR | Y | Y | N | N | NA | Y | N | Y | NR | NA | Y(3) |  | 66.7% |  | 58.3% |
| Paboriboune et al., 2014 | 1 | HHIV | Y | Y | NR | Y | N | N | N | NA | Y | Y | Y | NA | NA | NA |  | 75.0% |  |  |
| Efraim et al., 2014 | 1 | HHIV | Y | Y | Y | Y | N | N | N | NA | Y | N | Y | NA | NA | NA |  | 75.0% |  |  |
| Taye et al., 2014 | 1,3 | HHIV | Y | Y | NR | Y | N | N | N | NA | Y | N | Y | NR | NA | N(3) |  | 55.6% |  | 41.7% |
| Chatterjee et al., 2014 | 1 | HTB | Y | Y | Y | Y | N | N | N | NA | Y | N | Y | NR | NA | NA |  | 66.7% |  |  |
| Doumbo et al., 2014 | 3 | HH, HM | Y | Y | Y | Y | N | N | N | NA | Y | N | Y | NR | NA | N(3) |  |  | 54.5% | 50.0% |
| Mamo, 2014 | 3 | HH, HP | Y | Y | Y | Y | Y | N | N | NA | Y | N | Y | NA | NA | N(3) |  |  | 63.6% |  |
| Biraro et al., 2014 | 1,3 | HTB | Y | Y | Y | Y | Y | N | N | NA | Y | Y | Y | NR | NA | Y(3) |  | 88.9% |  | 75.0% |
| Belizario et al., 2014 | 3 | HTB | Y | Y | NR | Y | N | N | N | NA | Y | N | Y | NR | NA | N(3) |  |  |  | 41.7% |
| Perez-Porcuna et al., 2014 | 1 | HTB | Y | Y | Y | Y | Y | N | N | NA | Y | Y | Y | NR | NA | NA | 88.9% |  |  |  |
| Boonjaraspinyo et al., 2013 | 1,2,3 | HP (1,2); HH (3) | Y | N | NR | Y | N | N | N | NA | Y | N | Y | NA | NA | N(3) | 50.0% |  | 36.4% |  |
| Wassie et al., 2013 | 1 | HP | Y | Y | NR | Y | N | N | N | Y | Y | N | Y | NA | NA | NA | 62.5% |  |  |  |
| Oleyede et al., 2013 | 3 | HTB | Y | Y | Y | Y | N | N | N | NA | Y | N | Y | NR | NA | N(3) |  |  |  | 50.0% |
| Verhagen et al., 2013 | 1,2 | HP | Y | Y | NR | Y | N | N | N | NA | Y | Y | Y | NA | NA | NA | 75.0% |  |  |  |
| Sanchez et al., 2013 | 1,2 | HH | Y | Y | Y | Y | Y | N | N | NA | Y | N | Y | NA | NA | NA | 87.5% |  |  |  |
| Bustinduy et al., 2013 | 3 | HH, HM | Y | Y | NR | Y | N | N | N | NA | Y | N | Y | NA/NR(M) | NA | N(3) |  |  | 45.5% | 41.7% |
| Amollo et al., 2013 | 3 | HH | Y | Y | Y | Y | Y | N | N | NA | Y | N | Y | NA | NA | N(3) |  |  | 63.6% |  |
| Soares Magalhaes et al., 2013 | 3 | HH | Y | Y | Y | Y | Y | N | N | NA | Y | N | Y | NA | NA | N(3) |  |  | 63.6% |  |
| Meurs et al., 2013 | 3 | HH | Y | Y | Y | Y | N | N | N | NA | Y | Y | Y | NA | NA | N(3) |  |  | 63.6% |  |
| Arndt et al., 2013 | 1 | HHIV | Y | Y | Y | Y | N | N | N | NA | Y | N | Y | NA | NA | NA |  | 75.0% |  |  |
| Gouvras et al., 2013 | 3 | HH | Y | Y | NR | Y | N | N | N | NA | Y | N | Y | NA | NA | N(3) |  |  | 45.5% |  |
| Tchuem Tchuente et al., 2013 | 3 | HH | Y | Y | NR | Y | N | N | N | NA | Y | Y | Y | NA | NA | N(3) |  |  | 54.5% |  |
| Abanyie et al., 2013 | 3 | HM | Y | Y | N | Y | Y | N | N | NA | Y | N | Y | NR | NA | N(3) |  |  |  | 50.0% |
| Mulu et al., 2013 | 3 | HM | Y | N | NR | Y | N | N | N | NA | Y | N | Y | NR | NA | N(3) |  |  |  | 33.3% |
| Ugbomoiko et al., 2012 | 1,3 | HH | Y | Y | Y | Y | Y | N | N | NA | Y | N | Y | NA | NA | Y(3) | 87.5% |  | 72.7% |  |
| Coulibaly et al., 2012 | 1,3 | HH, HP | Y | Y | Y | Y | Y | N | N | NA | Y | Y | Y | NA | NA | Y(3) | 100.0% |  | 81.8% |  |
| Odiere et al., 2012 | 1,2 | HH | Y | Y | NR | Y | Y | N | N | NA | Y | N | Y | NA | NA | NA | 75.0% |  |  |  |
| Righetti et al., 2012 | 3 | HM | Y | Y | N | Y | Y | N | N | NA | Y | N | Y | NR | NA | Y(3) |  |  |  | 58.3% |
| Florey et al., 2012 | 3 | HM | Y | Y | N | Y | N | N | N | NA | Y | Y | Y | NR | NA | N(3) |  |  |  | 50.0% |
| Samuels et al., 2012 | 3 | HH; HM | Y | Y | NR | Y | N | N | N | NA | Y | Y | Y | NA/NR(M) | NA | N(3) |  |  | 54.5% | 50.0% |
| Conlan et al., 2012 | 3 | HH | Y | Y | Y | Y | Y | N | N | NA | Y | N | Y | NA | NA | Y(3) |  |  | 72.7% |  |
| Wumba et al., 2012 | 1 | HHIV | Y | Y | Y | Y | N | N | N | NA | Y | N | NR | NA | NA | NA |  | 62.5% |  |  |
| Boaitey et al., 2012 | 3 | HHIV | Y | Y | NR | Y | N | N | N | NA | Y | N | Y | NR | NA | N(3) |  |  |  | 41.7% |
| Humphries et al., 2011 | 3 | HM | Y | Y | Y | Y | Y | N | N | NA | Y | N | Y | NR | NA | Y(3) |  |  |  | 66.7% |
| Njenga et al., 2011 | 1,2 | HH | Y | Y | NR | Y | N | N | N | NA | Y | N | Y | NA | NA | NA | 62.5% |  |  |  |
| Pilger et al., 2011 | 1,2 | HH | Y | Y | Y | Y | Y | N | N | NA | Y | N | Y | NA | NA | NA | 87.5% |  |  |  |
| Traore et al., 2011 | 1 | HP | Y | Y | Y | Y | Y | N | N | NA | Y | N | Y | NA | NA | NA | 87.5% |  |  |  |
| Goncalves et al., 2011 | 1,2 | HP | Y | Y | NR | Y | Y | N | N | NA | Y | Y | Y | NA | NA | NA | 87.5% |  |  |  |
| Matthys et al., 2011 | 1 | HP | Y | Y | Y | Y | Y | N | N | NA | Y | N | Y | NA | NA | NA | 87.5% |  |  |  |
| Muller et al., 2011 | 1,2,3 | HM, HH | Y | Y | Y | Y | Y | N | N | NA | Y | Y | Y | NA/NR(M) | NA | N(3) | 100.0% | 88.9% | 72.7% | 66.7% |
| Akue et al., 2011 | 3 | HH | Y | Y | NR | Y | Y | N | N | NA | Y | N | Y | NA | NA | N(3) |  |  | 54.5% |  |
| Sanyaolu et al., 2011 | 3 | HHIV | Y | Y | Y | Y | N | N | N | NA | Y | N | Y | Yes | NA | Y(3) |  |  |  | 66.7% |
| Asma et al., 2011 | 1 | HHIV | Y | Y | NR | Y | N | N | N | NA | Y | N | NR | NA | NA | NA |  | 50.0% |  |  |
| Idindili et al., 2011 | 1 | HHIV | Y | Y | NR | Y | N | N | N | NA | Y | N | Y | NA | NA | NA |  | 62.5% |  |  |
| Pullan et al., 2010 | 3 | HM | Y | Y | Y | Y | N | N | N | NA | Y | Y | Y | NR | NA | NT3) |  |  |  | 58.3% |
| Midzi et al., 2010 | 1 | HM | Y | Y | NR | Y | Y | N | N | NA | Y | Y | Y | NR | NA | NA |  | 77.8% |  |  |
| Mahgoub et al., 2010 | 3 | HH | Y | Y | NR | Y | N | N | N | NA | Y | Y | Y | NA | NA | N(3) |  |  | 54.5% |  |
| Mwambete et al., 2010 | 1,3 | HHIV | Y | Y | NR | Y | Y | N | N | NA | Y | N | Y | NR | NA | N(3) |  | 66.7% |  | 50.0% |
| Walson et al., 2010 | 1 | HHIV | Y | Y | NR | Y | N | N | N | NA | Y | N | Y | NR | NA | NA |  | 55.6% |  |  |
| Koukounari et al., 2010 | 3 | HH | Y | Y | NR | Y | Y | N | N | NA | Y | Y | Y | NA | NA | N(3) |  |  | 63.6% |  |
| Mazigo et al., 2010 | 3 | HM | Y | Y | NR | Y | N | N | N | NA | Y | N | Y | NR | NA | Y(3) |  |  |  | 50.0% |
| Babatunde et al., 2010 | 3 | HHIV | Y | N | NR | Y | N | N | N | NA | Y | N | Y | NR | NA | UC |  |  |  | 33.3% |
| Nguhiu et al., 2009 | 3 | HH; HP | Y | Y | Y | Y | N | N | N | NA | Y | N | Y | NA | NA | N(3) |  |  | 54.5% |  |
| Hamm et al., 2009 | 1,3 | HP (1), HH (3) | Y | Y | NR | Y | N | N | N | NA | Y | Y | Y | NA | NA | N(3) | 75.0% |  | 54.5% |  |
| Kung'u et al., 2009 | 3 | HM | Y | Y | NR | Y | N | N | N | NA | Y | Y | Y | NR | NA | Y(3) |  |  |  | 58.3% |
| Assefa et al., 2009 | 3 | HHIV | Y | Y | NR | Y | N | N | N | NA | Y | N | Y | NR | NA | N(3) |  |  |  | 41.7% |
| Anah et al., 2008 | 1,2,3 | HH | Y | Y | NR | Y | N | N | N | NA | Y | N | Y | NA | NA | N(3) | 62.5% |  | 45.5% |  |
| Sousa-Figueiredo et al., 2008 | 1 | HH | Y | Y | Y | Y | Y | N | N | NA | Y | N | Y | NA | NA | NA | 87.5% |  |  |  |
| Tengco et al., 2008 | 1,2 | HH | Y | Y | Y | Y | N | N | N | NA | Y | N | Y | NA | NA | NA | 75.0% |  |  |  |
| Jardim-Botelho et al., 2008 | 1,2 | HH | Y | Y | NR | Y | N | N | N | NA | Y | N | Y | NA | NA | NA | 62.5% |  |  |  |
| Ezeamama et al., 2008 | 1 | HH | Y | Y | Y | Y | N | N | N | NA | Y | Y | Y | NA | NA | NA | 87.5% |  |  |  |
| Korkes et al., 2008 | 1 | HP | Y | Y | N | Y | N | N | N | NA | Y | N | Y | NA | NA | NA | 62.5% |  |  |  |
| Nematian et al., 2008 | 1,2 | HP | Y | Y | NR | Y | N | N | N | NA | Y | N | Y | NA | NA | NA | 62.5% |  |  |  |
| Midzi et al., 2008 | 3 | HM | Y | Y | NR | Y | N | N | N | NA | Y | Y | Y | NR | NA | N(3) |  |  |  | 50.0% |
| Kassu et al., 2007 | 1 | HTB | Y | Y | NR | Y | N | N | N | NA | Y | Y | Y | NA | NA | NA |  | 75.0% |  |  |
| Hosseinipour et al., 2007 | 1,3 | HHIV | Y | Y | NR | Y | N | N | N | NA | Y | N | Y | NR | NA | N(3) |  | 55.6% |  | 41.7% |
| Nkuo-Akenji et al, 2006 | 1,3 | HH, HM | Y | Y | Y | Y | N | N | N | NA | Y | N | Y | NA/NR(M) | NA | N(3) | 75.0% | 66.7% | 54.5% | 50.0% |
| Raso et al., 2006 | 3 | HH | Y | Y | Y | Y | N | N | N | NA | Y | N | Y | NA | NA | N(3) |  |  | 54.5% |  |
| Fleming et al., 2006 | 1,2,3 | HH | Y | Y | NR | Y | N | N | N | NA | Y | N | Y | NA | NA | Y(3) | 62.5% |  | 54.5% |  |
| Ramos et al., 2006 | 1,3 | HTB | Y | Y | Y | Y | N | N | N | NA | Y | N | Y | NR | NA | Y(3) |  | 66.7% |  | 58.3% |
| Resende Co et al., 2006 | 1 | HTB | Y | Y | NR | Y | N | N | N | NA | Y | Y | Y | NA | NA | NA |  | 75.0% |  |  |
| Nielsen et al., 2006 | 3 | HH, HM, HHIV | Y | Y | NR | Y | N | N | N | NA | Y | N | Y | NR | NA | Y(3) |  |  | 54.5% | 50.0% |
| Moges et al., 2006 | 3 | HHIV | Y | Y | NR | Y | N | N | N | NA | Y | N | Y | NR | NA | N(3) |  |  |  | 41.7% |
| Modjarrad et al., 2005 | 1 | HHIV | Y | Y | NR | Y | N | N | N | NA | Y | Y | Y | NA | NA | NA |  | 75.0% |  |  |
| Briand et al., 2005 | 1,2,3 | HH | Y | Y | Y | Y | N | N | N | NA | Y | N | Y | NA | NA | N(3) | 75.0% |  | 54.5% |  |
| Bhumiratana et al., 2005 | 3 | HH | Y | Y | NR | Y | N | N | N | NA | Y | N | Y | NA | NA | N(3) |  |  | 45.5% |  |
| Kallestrup et al., 2005 | 1,3 | HHIV | Y | Y | NR | Y | N | N | N | NA | Y | N | Y | Yes | NA | N(3) |  | 66.7% |  | 50.0% |
| Lyke et al., 2005 | 3 | HM | Y | Y | N | Y | Y | Y | Y | NA | Y | N | Y | NR | Yes | N(3) |  |  |  | 69.2% |
| Shapiro et al., 2005 | 3 | HM | Y | Y | Y | Y | N | N | Y | NA | Y | N | Y | NR | Yes | Y(3) |  |  |  | 69.2% |
| Singh et al., 2004 | 1 | HHIV | Y | Y | NR | Y | N | N | N | NA | Y | N | Y | NA | NA | NA |  | 62.5% |  |  |
| Quihui-Cota et al., 2004 | 1 | HP | Y | Y | N | Y | Y | N | N | NA | Y | Y | Y | NA | NA | NA | 87.5% |  |  |  |
| Adio et al., 2004 | 1 | HM | Y | Y | NR | Y | N | N | N | NA | Y | N | Y | NR | NA | NA |  | 55.6% |  |  |
| Raso et al., 2004 | 3 | HH; HP | Y | Y | Y | Y | Y | N | N | NA | Y | Y | Y | NA | NA | Y(3) |  |  | 81.8% |  |
| Brown et al., 2004 | 1 | HHIV | Y | Y | Y | Y | N | N | N | NA | Y | N | Y | NA | Yes | NA |  | 75.0% |  |  |
| Engelbrecht et al., 2003 | 3 | HH | Y | Y | NR | Y | N | N | N | NA | Y | N | Y | NR | NA | N(3) |  |  | 45.5% | 41.7% |
| Tchuem Tchuente et al., 2003 | 1,2 | HH | Y | Y | Y | Y | Y | N | N | NA | Y | N | Y | NA | NA | NA | 87.5% |  |  |  |
| Brown et al., 2003 | 1 | HHIV | Y | Y | Y | Y | N | N | N | NA | Y | N | Y | NA | NA | NA |  | 75.0% |  |  |
| Keiser et al., 2002 | 1,3 | HP, HH(3) | Y | Y | Y | Y | N | N | N | NA | Y | N | Y | NA | NA | Y(3) | 75.0% |  | 63.6% |  |
| Keiser et al., 2002b | 3 | HP, HH | Y | Y | Y | Y | N | N | N | NA | Y | Y | Y | NA | NA | Y(3) |  |  | 72.7% |  |
| Nacher et al., 2002 | 3 | HM | Y | Y | Y | Y | N | Y | Y | NA | Y | N | Y | NR | NR | Y(3) |  |  |  | 69.2% |
| van den Biggelaar et al., 2001 | 1 | HM | Y | Y | NR | Y | N | N | N | NA | Y | N | Y | NR | NA | NA |  | 55.6% |  |  |
| Thiong'o et al., 2001 | 1,2 | HH | Y | Y | NR | Y | N | N | N | NA | Y | N | Y | NA | NA | NA | 62.5% |  |  |  |
| Smith et al., 2001 | 3 | HH | Y | Y | NR | Y | N | N | N | NA | Y | N | Y | NA | NA | N(3) |  |  | 45.5% |  |
| Feitosa et al., 2001 | 3 | HHIV | Y | Y | N | Y | N | N | N | NA | Y | N | Y | Yes | NA | N(3) |  |  |  | 50.0% |
| Guignard et al., 2000 | 1 | HP | Y | Y | Y | Y | N | N | N | NA | Y | N | Y | NA | NA | NA | 75.0% |  |  |  |
| Brooker et al., 2000 | 1,2 | HH | Y | Y | Y | Y | N | N | N | NA | Y | N | Y | NA | NA | NA | 75.0% |  |  |  |
| Scolari et al., 2000 | 1,2 | HH | Y | Y | Y | Y | Y | N | N | NA | Y | N | Y | NA | NA | NA | 87.5% |  |  |  |
| Lili et al., 2000 | 1,2 | HH | Y | N | NR | Y | N | N | N | NA | Y | N | Y | NA | NA | NA | 50.0% |  |  |  |
| Widjana et al., 2000 | 1,2 | HH | Y | Y | Y | Y | N | N | N | NA | Y | N | Y | NA | NA | NA | 75.0% |  |  |  |
| Al-Agha et al., 2000 | 1,2,3 | HP, HH(3) | Y | Y | NR | Y | N | N | N | NA | Y | N | Y | NA | NA | N(3) | 62.5% |  | 45.5% |  |
| Stoltzfus et al., 2000 | 3 | HH | Y | Y | Y | Y | N | N | N | NA | Y | N | Y | NA | NA | Y(3) |  |  | 63.6% |  |
| Fontanet et al., 2000 | 3 | HHIV | Y | Y | Y | Y | N | N | N | NA | Y | N | Y | NR | NA | N(3) |  |  |  | 50.0% |
| Saldiva et al., 1999 | 1,3 | HP, HH(3) | Y | Y | Y | Y | N | N | N | NA | Y | Y | Y | NA | NA | N(3) | 87.5% |  | 63.6% |  |
| Toma et al., 1999 | 1,2 | HH | Y | Y | N | Y | N | N | N | NA | Y | N | Y | NA | NA | NA | 62.5% |  |  |  |
| Brooker et al., 1999 | 3 | HH, HM | Y | Y | NR | Y | Y | N | N | NA | Y | N | Y | NR | NA | N(3) |  |  | 54.5% | 50.0% |
| Booth et al., 1998 | 1,2,3 | HH | Y | Y | Y | Y | N | N | N | NA | Y | N | Y | NA | NA | N(3) | 75.0% |  | 54.5% |  |
| Needham et al., 1998 | 1,2,3 | HH | Y | Y | Y | Y | N | N | N | NA | Y | N | Y | NA | NA | N(3) | 75.0% |  | 54.5% |  |
| Gamboa et al., 1998 | 1,2 | HP | Y | Y | NR | Y | N | N | N | NA | Y | Y | Y | NA | NA | NA | 75.0% |  |  |  |
| Kang et al., 1998 | 1,2 | HH, HP | Y | Y | NR | Y | N | N | N | NA | Y | Y | Y | NA | NA | NA | 75.0% |  |  |  |
| Ravindran et al., 1998 | 3 | HM | Y | N | NR | Y | N | N | N | NA | Y | N | Y | NR | NA | N(3) |  |  |  | 33.3% |
| Albonico et al., 1997 | 1,2 | HH | Y | Y | Y | Y | N | N | N | NA | Y | N | Y | NA | NA | NA | 75.0% |  |  |  |
| Booth et al., 1996 | 1,2 | HH | Y | Y | NR | Y | N | N | N | NA | Y | N | Y | NA | NA | NA | 62.5% |  |  |  |
| Hadju et al., 1995 | 3 | HH | Y | Y | Y | Y | N | N | N | NA | Y | N | Y | NA | NA | N(3) |  |  | 54.5% |  |
| Chunge et al., 1995 | 1 | HP | Y | Y | NR | Y | N | N | N | NA | Y | N | Y | NA | NA | NA | 62.5% |  |  |  |
| Fischer et al., 1995 | 3 | HHIV | Y | Y | N | Y | N | N | N | NA | Y | N | Y | NR | NA | Y(3) |  |  |  | 50.0% |
| Birrie et al., 1994 | 1,2 | HH | Y | Y | NR | Y | N | N | N | NA | Y | N | Y | NA | NA | NA | 62.5% |  |  |  |
| Gbakima et al.,1994 | 1,2 | HH | Y | Y | NR | Y | N | N | N | NA | Y | N | Y | NA | NA | NA | 62.5% |  |  |  |
| Ferreira et al., 1994 | 1,3 | HP, HH(3) | Y | Y | Y | Y | N | N | N | NA | Y | N | Y | NA | NA | N(3) | 75.0% |  | 54.5% |  |
| Schuurkamp, 1994 | 3 | HM | Y | Y | NR | UC | N | N | N | NA | Y | N | Y | NR | NA | N(3) |  |  |  | 33.3% |
| Gallin et al., 1993 | 3 | HHIV | Y | N | NR | Y | N | N | N | NA | Y | N | Y | NR | NA | N(3) |  |  |  | 33.3% |
| McGarvey et al., 1992 | 3 | HH | Y | Y | Y | Y | N | N | N | NA | Y | N | Y | NA | NA | N(3) |  |  | 54.5% |  |
| Robertson et al., 1992 | 3 | HH | Y | Y | NR | Y | N | N | N | NA | Y | N | Y | NA | NA | N(3) |  |  | 45.5% |  |
| Chunge et al., 1991 | 1,2 | HP | Y | Y | NR | Y | N | N | N | NA | Y | N | Y | NA | NA | NA | 62.5% |  |  |  |
| Upatham et al., 1989 | 1,3 | HH | Y | Y | Y | Y | N | N | N | NA | Y | N | Y | NA | NA | N(3) | 75.0% |  | 54.5% |  |
| Holland et al., 1987 | 1,2,3 | HH, HP | Y | Y | NR | Y | N | N | N | NA | Y | N | Y | NA | NA | N(3) | 62.5% |  | 45.5% |  |
| Annan et al., 1986 | 1,2 | HP | Y | Y | NR | Y | N | N | N | NA | Y | N | Y | NA | NA | NA | 62.5% |  |  |  |
| Higgins et al., 1984 | 1,2,3 | HH | Y | Y | Y | Y | N | N | N | NA | Y | N | Y | NA | NA | N(3) | 75.0% |  | 54.5% |  |
| Adeyeba et al, 1984 | 3 | HH, HP | Y | N | NR | Y | N | N | N | NA | Y | N | Y | NA | NA | N(3) |  |  | 36.4% |  |
| Kassim et al., 1982 | 3 | HM | Y | N | NR | Y | N | N | N | NA | Y | N | Y | NR | NA | N(3) |  |  |  | 33.3% |
| Ismid et al., 1981 | 1,2,3 | HH | Y | Y | NR | Y | N | N | N | NA | Y | N | Y | NA | NA | N(3) | 62.5% |  | 45.5% |  |
| Sinniah et al, 1978 | 1,2,3 | HH | Y | Y | NR | Y | N | N | N | NA | Y | N | Y | NA | NA | N(3) | 62.5% |  | 45.5% |  |
| Carney et al., 1974 | 1 | HP | Y | N | NR | Y | N | N | N | NA | Y | N | Y | NA | NA | NA | 50.0% |  |  |  |
| Carney et al., 1974b | 1 | HP | Y | N | NR | Y | N | N | N | NA | Y | N | Y | NA | NA | NA | 50.0% |  |  |  |
| Buck et al., 1969 | 3 | HH | Y | Y | Y | Y | N | N | N | NA | Y | N | Y | NR | NA | N(3) | 75.0% |  | 50.0% |  |
