# Supplementary material for: The extent, nature, and pathogenic consequences of helminth polyparasitism in humans: A meta-analysis
Source: PLoS Negl Trop Dis. 2019 Jun 18;13(6):e0007455. doi: 10.1371/journal.pntd.0007455 (PMC6599140; doi:10.1371/journal.pntd.0007455)
Supplement: S7 Table — (DOCX) [file pntd.0007455.s009.docx]

**S7 Table.** **Quality assessment scores for each study considered in this meta-analysis using the NIH Quality Assessment Tool for Case-Control Studies.** HH = Helminth-helminth; HM = Helminth-malaria; HTB = Helminth-tuberculosis; HHIV = Helminth-HIV; T1 = Type 1 (prevalence difference); T3 = Type 3 (association); Y = Yes; N= No; NR = Not reported; NA = Not applicable.

Questions corresponding to the number listed in the table are presented here:

1. Was the research question or objective in this paper clearly stated and appropriate?
2. Was the study population clearly specified and defined?
3. Did the authors include a sample size justification?
4. Were controls selected or recruited from the same or similar population that gave rise to the cases (including the same timeframe)?
5. Were the definitions, inclusion and exclusion criteria, algorithms or processes used to identify or select cases and controls valid, reliable, and implemented consistently across all study participants?
6. Were the cases clearly defined and differentiated from controls?
7. If less than 100 percent of eligible cases and/or controls were selected for the study, were the cases and/or controls randomly selected from those eligible?
8. Was there use of concurrent controls?
9. Were the investigators able to confirm that the exposure/risk occurred prior to the development of the condition or event that defined a participant as a case?
10. Were the measures of exposure/risk clearly defined, valid, reliable, and implemented consistently (including the same time period) across all study participants?
11. Were the assessors of exposure/risk blinded to the case or control status of participants?
12. Were key potential confounding variables measured and adjusted statistically in the analyses? If matching was used, did the investigators account for matching during study analysis?

| **Author** | **Data Type** | **Data Pair** | **Question Number** | | | | | | | | | | | | **Quality Assessment Scores** | |
| --- | --- | --- | --- | --- | --- | --- | --- | --- | --- | --- | --- | --- | --- | --- | --- | --- |
|  |  |  | **1** | **2** | **3** | **4** | **5** | **6** | **7** | **8** | **9** | **10** | **11** | **12** | **T1** | **T3** |
| Tian et al., 2012 | 1,3 | HHIV | Y | Y | N | Y | Y | Y | NR | NA | N | Y | Y | N(T3) | 70.0% | 63.6% |
| Da Silva et al., 2005 | 1,3 | HHIV | Y | Y | N | Y | Y | Y | NR | NA | N | Y | NR | N(T3) | 60.0% | 54.5% |
| Mkhize-Kwitshana et al., 2011 | 1,3 | HHIV | Y | Y | N | Y | Y | Y | NR | NA | N | Y | NR | N(T3) | 60.0% | 54.5% |
| Roka et al., 2013 | 1,3 | HHIV | Y | Y | Y | Y | Y | Y | Y | NA | N | Y | NR | N(T3) | 80.0% | 72.7% |
| Roka et al., 2012 | 1,3 | HHIV | Y | Y | N | Y | Y | Y | NR | NA | N | Y | NR | N(T3) | 60.0% | 54.5% |
| Mhimbira et al., 2017 | 1,3 | HTB | Y | Y | Y | Y | Y | Y | NR | NA | N | Y | NR | Y(T3) | 70.0% | 72.7% |
| Abate et al., 2015 | 1,3 | HTB | Y | Y | N | Y | Y | Y | NR | NA | N | Y | NR | N(T3) | 60.0% | 54.5% |
| Abate et al., 2012 | 1,3 | HTB | Y | Y | N | Y | Y | Y | NR | NA | N | Y | NR | N(T3) | 60.0% | 54.5% |
| Elias et al., 2006 | 1,3 | HTB | Y | Y | N | Y | Y | Y | NR | NA | N | Y | NR | Y(T3) | 60.0% | 63.6% |
| Tristao-Sa et al., 2002 | 1,3 | HTB | Y | Y | N | Y | Y | Y | NR | NA | N | Y | NR | Y(T3) | 60.0% | 63.6% |
| Fernandez-Nino et al., 2012 | 3 | HM | Y | Y | N | Y | Y | Y | NR | NA | N | Y | Y | Y(T3) | 70.0% | 72.7% |
| Efunshile et al., 2015 | 3 | HM | Y | Y | Y | Y | Y | Y | NR | NA | N | Y | N | N(T3) | 70.0% | 63.6% |
| Le Hesran et al., 2004 | 3 | HM | Y | Y | N | Y | Y | N | NR | NA | N | Y | NR | N(T3) | 50.0% | 45.5% |
| Jongwutiwes et al., 2014 | 3 | HHIV | Y | Y | N | Y | Y | Y | NR | NA | N | Y | NR | N(T3) | 60.0% | 54.5% |
| Escobedo et al., 1999 | 3 | HHIV | Y | N | N | Y | Y | Y | NR | NA | N | Y | NR | N(T3) | 50.0% | 45.5% |
| Hailemariam et al., 2004 | 3 | HHIV | Y | Y | N | Y | Y | Y | NR | NA | N | Y | NR | N(T3) | 60.0% | 54.5% |
| Marchi Blatt et al., 2003 | 3 | HHIV | Y | N | N | Y | Y | Y | NR | NA | N | Y | NR | N(T3) | 50.0% | 45.5% |
| Parraga et al., 1996 | 3 | HH | Y | Y | N | Y | Y | Y | NR | NA | N | Y | NA | N(T3) | 66.7% | 60.0% |
| Cabral et al., 2015 | 3 | HHIV, HTB | Y | Y | N | Y | Y | Y | NR | NA | N | NR | NR | Y(T3) |  | 54.5% |
