# Supplementary figures and images for: The extent, nature, and pathogenic consequences of helminth polyparasitism in humans: A meta-analysis
Source: PLoS Negl Trop Dis. 2019 Jun 18;13(6):e0007455. doi: 10.1371/journal.pntd.0007455 (PMC6599140; doi:10.1371/journal.pntd.0007455)

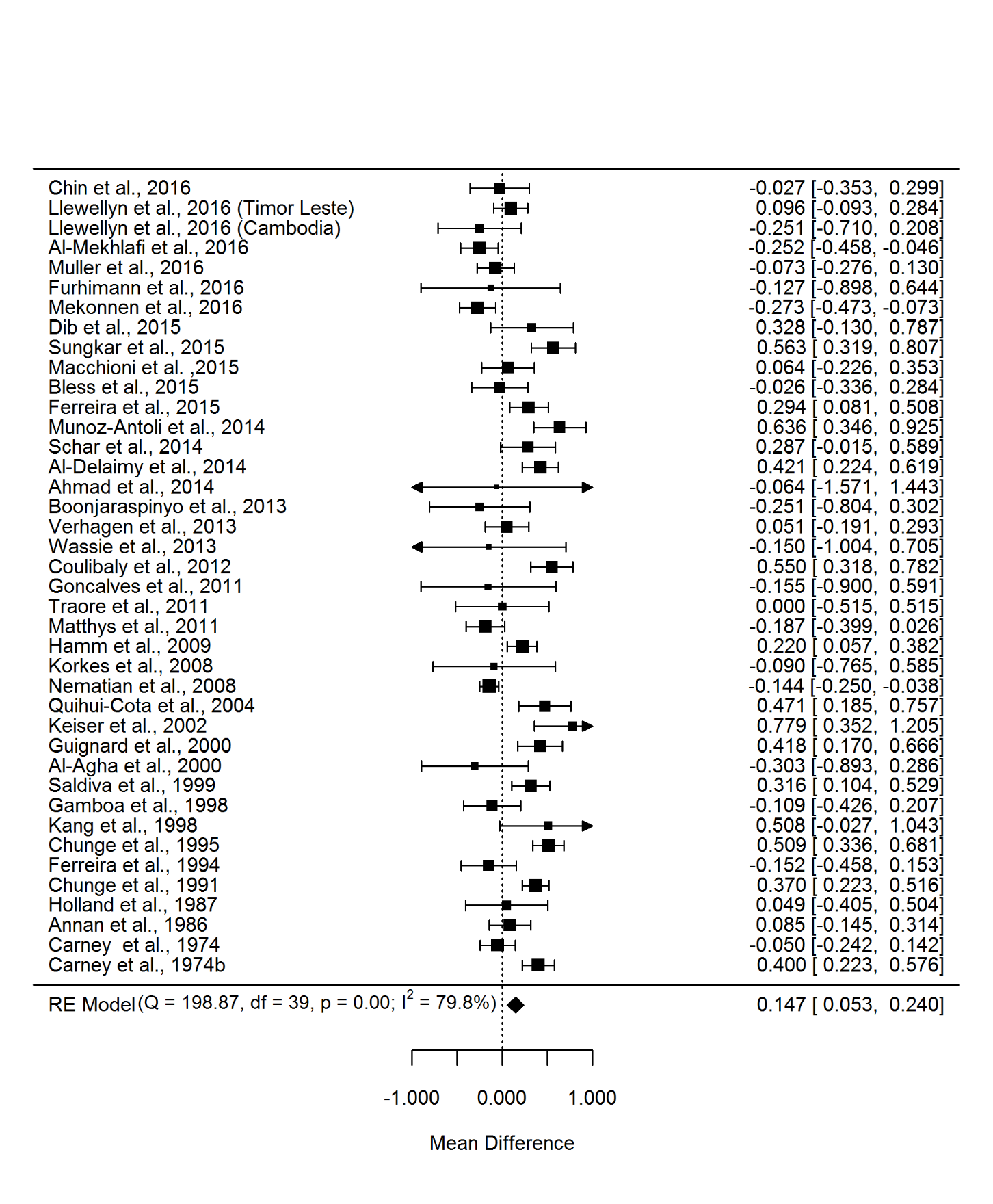

Supplement: S1 Fig — (TIF) [file pntd.0007455.s010.tif]

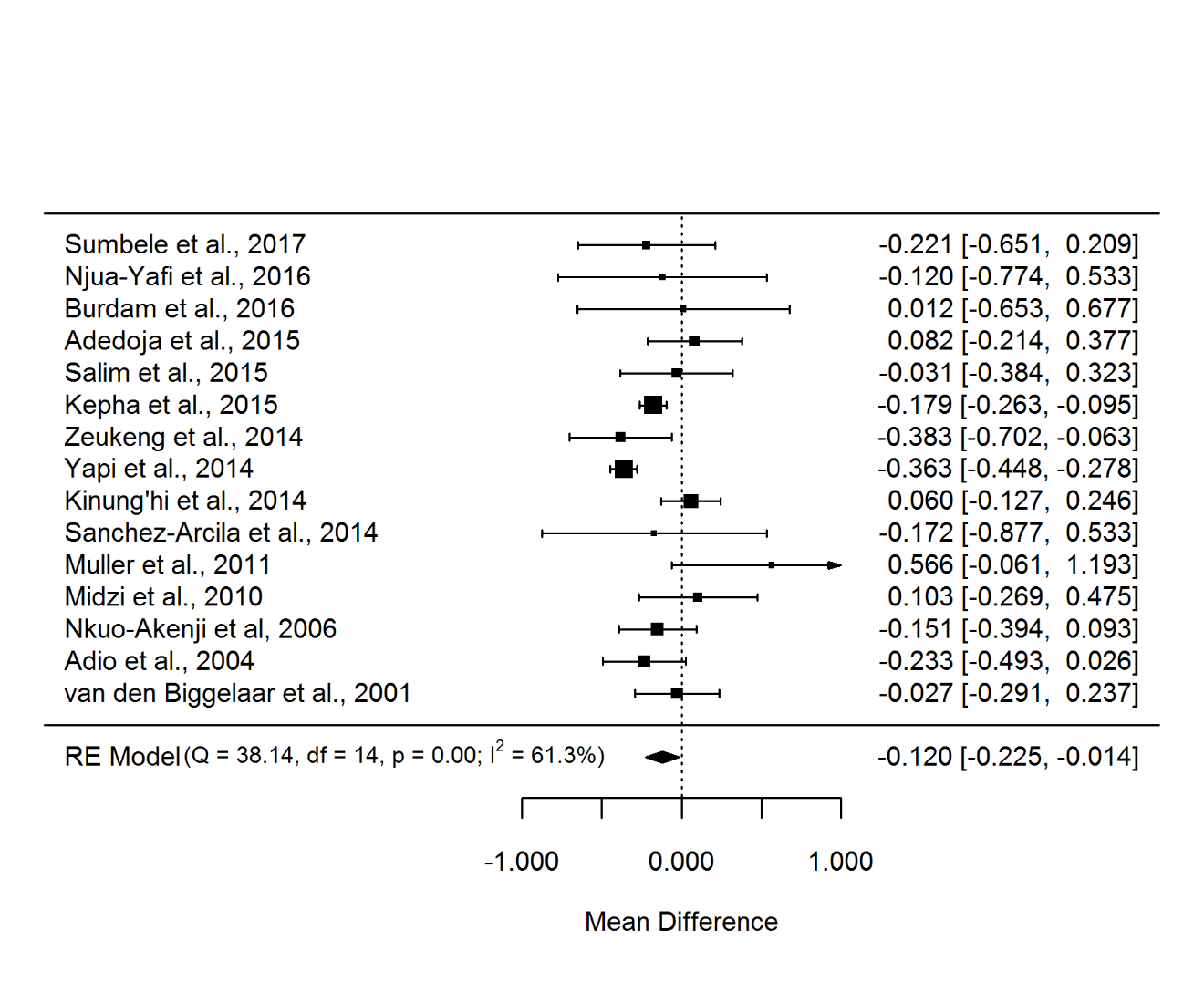

Supplement: S2 Fig — (TIF) [file pntd.0007455.s011.tif]

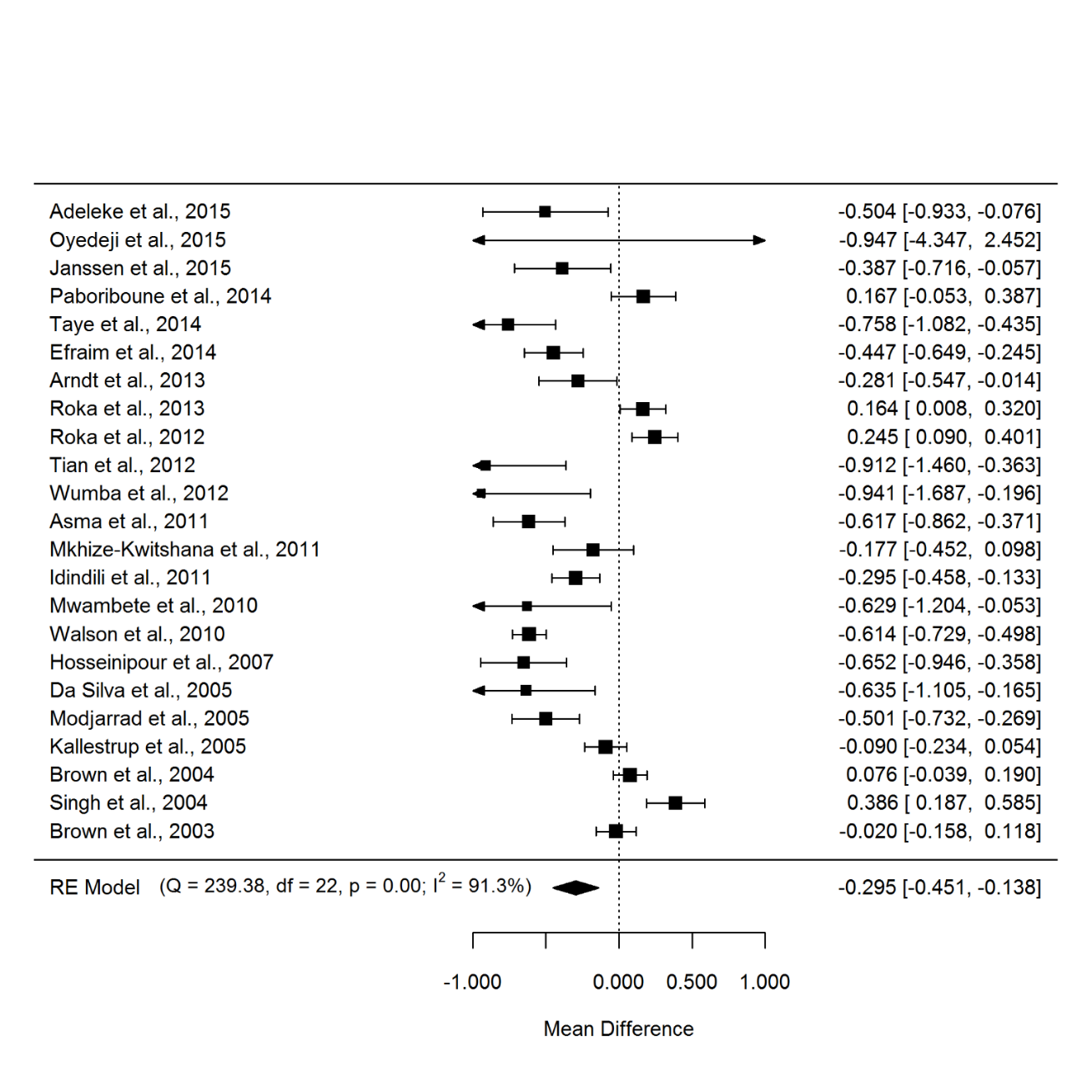

Supplement: S3 Fig — (TIF) [file pntd.0007455.s012.tif]

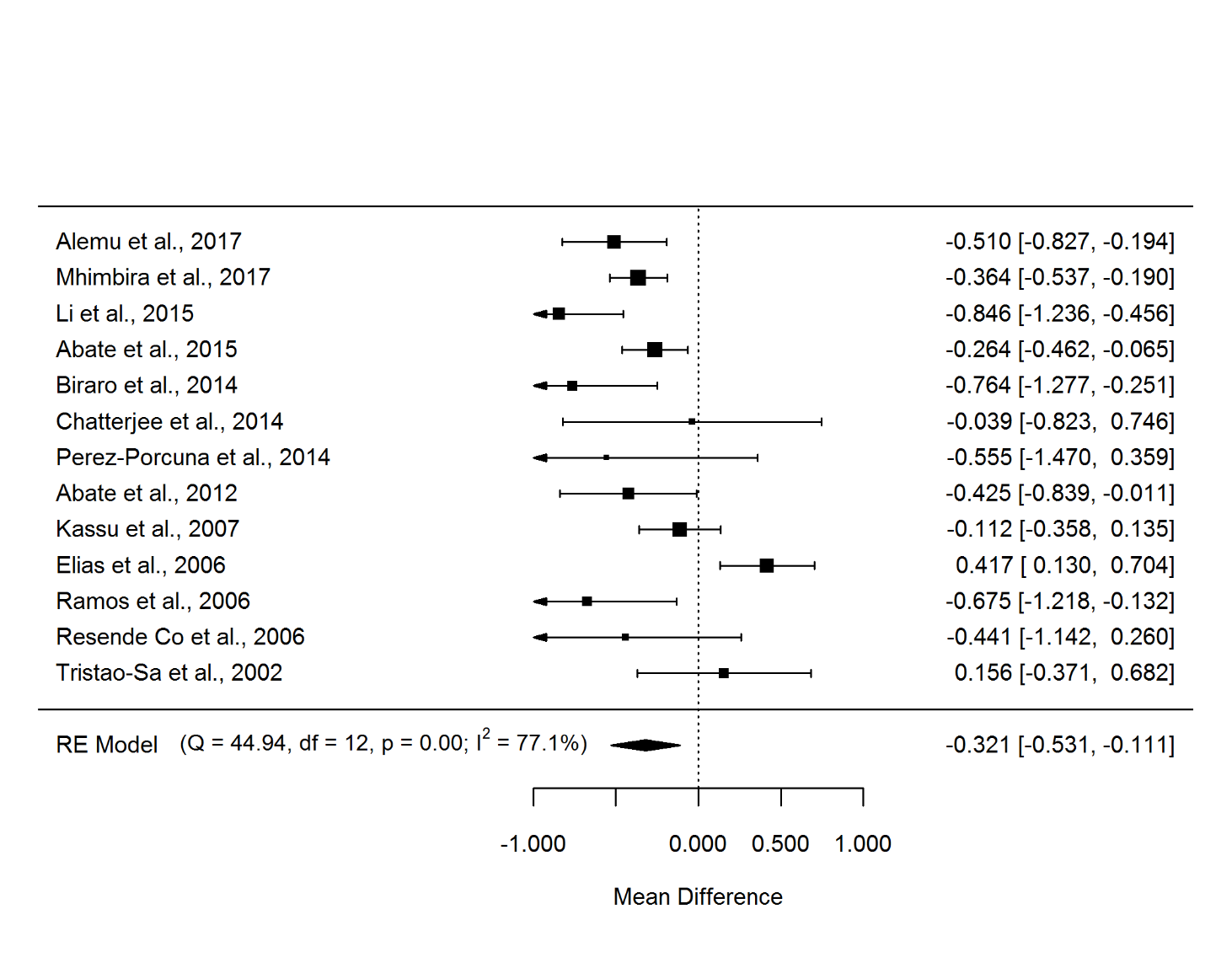

Supplement: S4 Fig — (TIF) [file pntd.0007455.s013.tif]

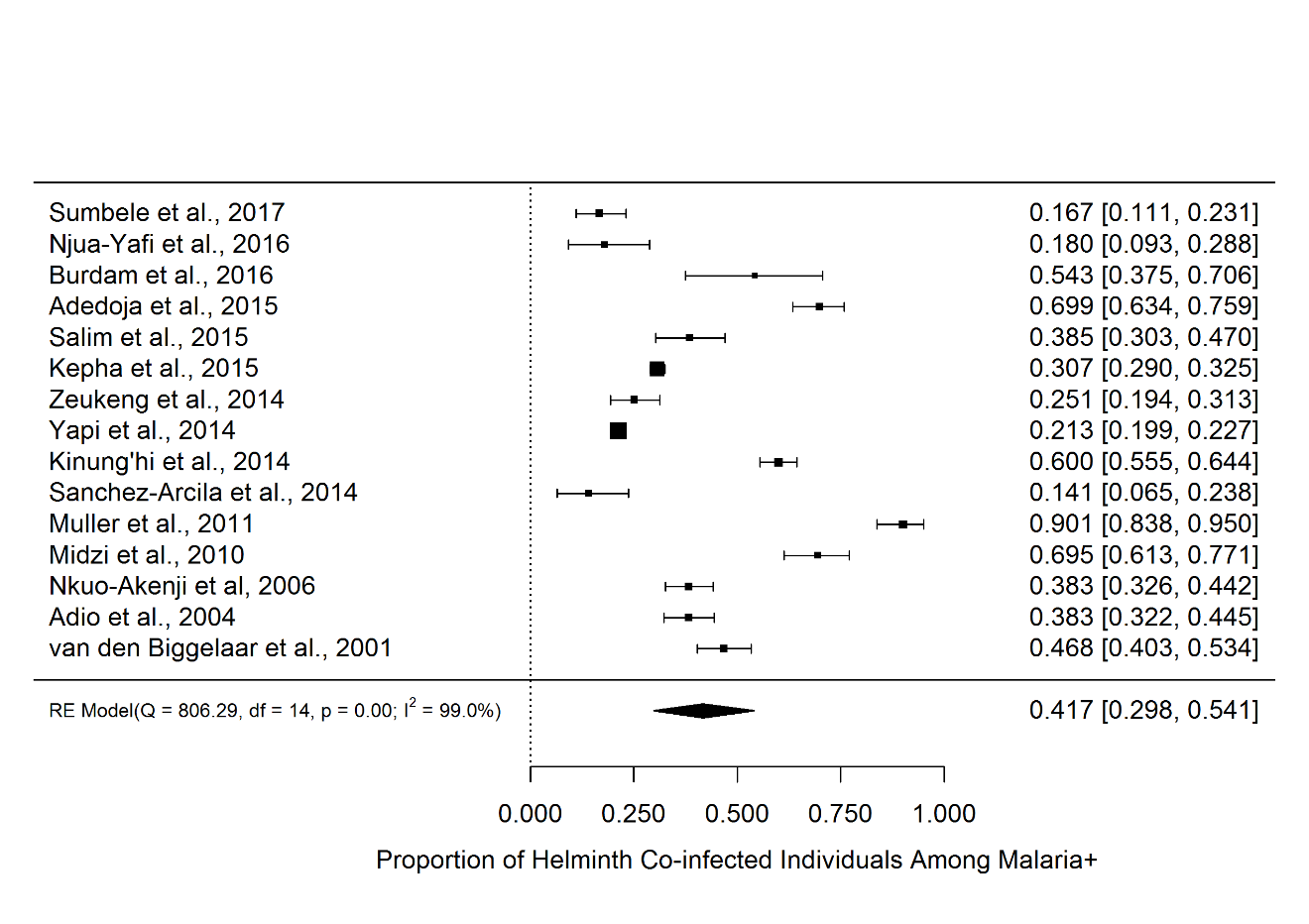

Supplement: S5 Fig — (TIF) [file pntd.0007455.s014.tif]

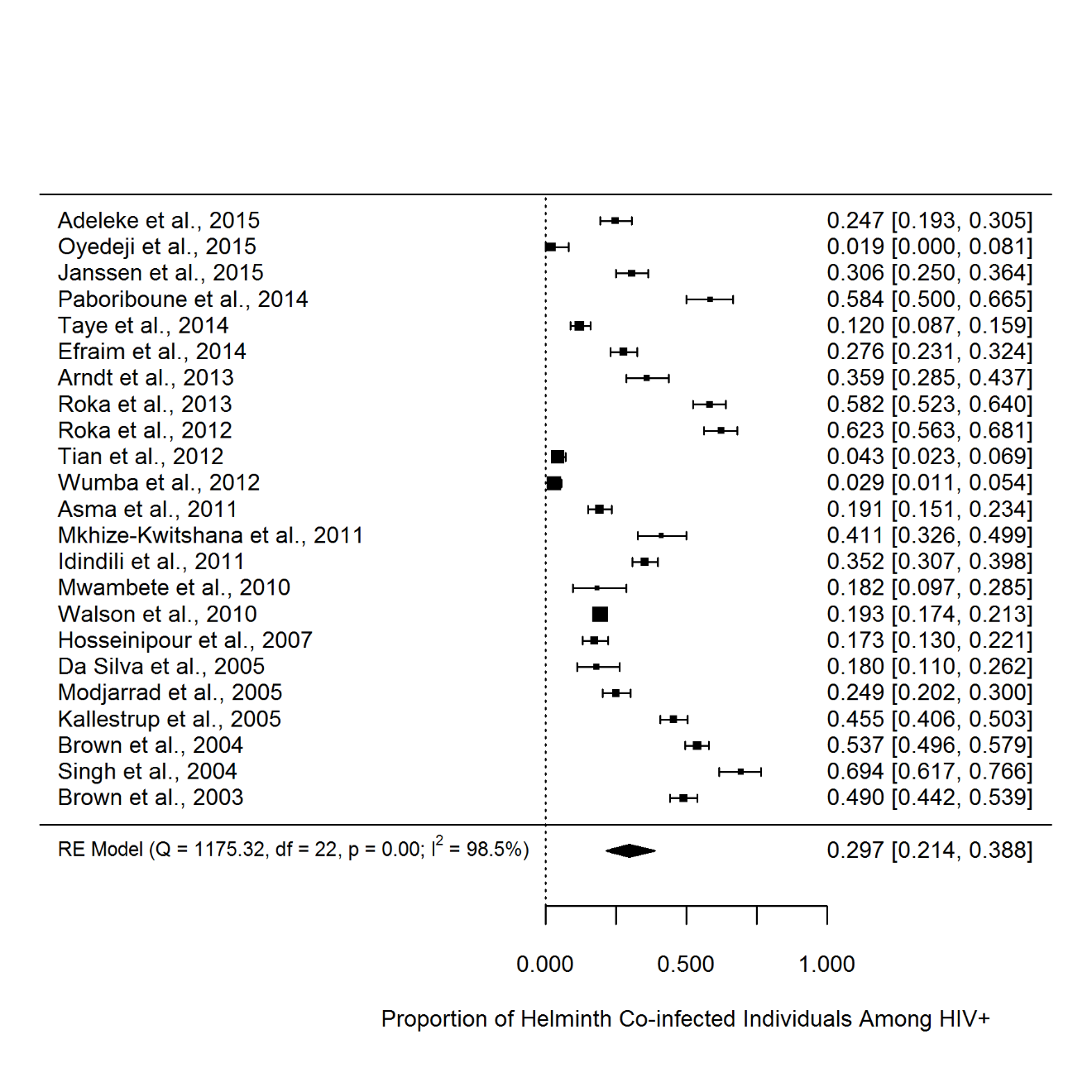

Supplement: S6 Fig — (TIF) [file pntd.0007455.s015.tif]

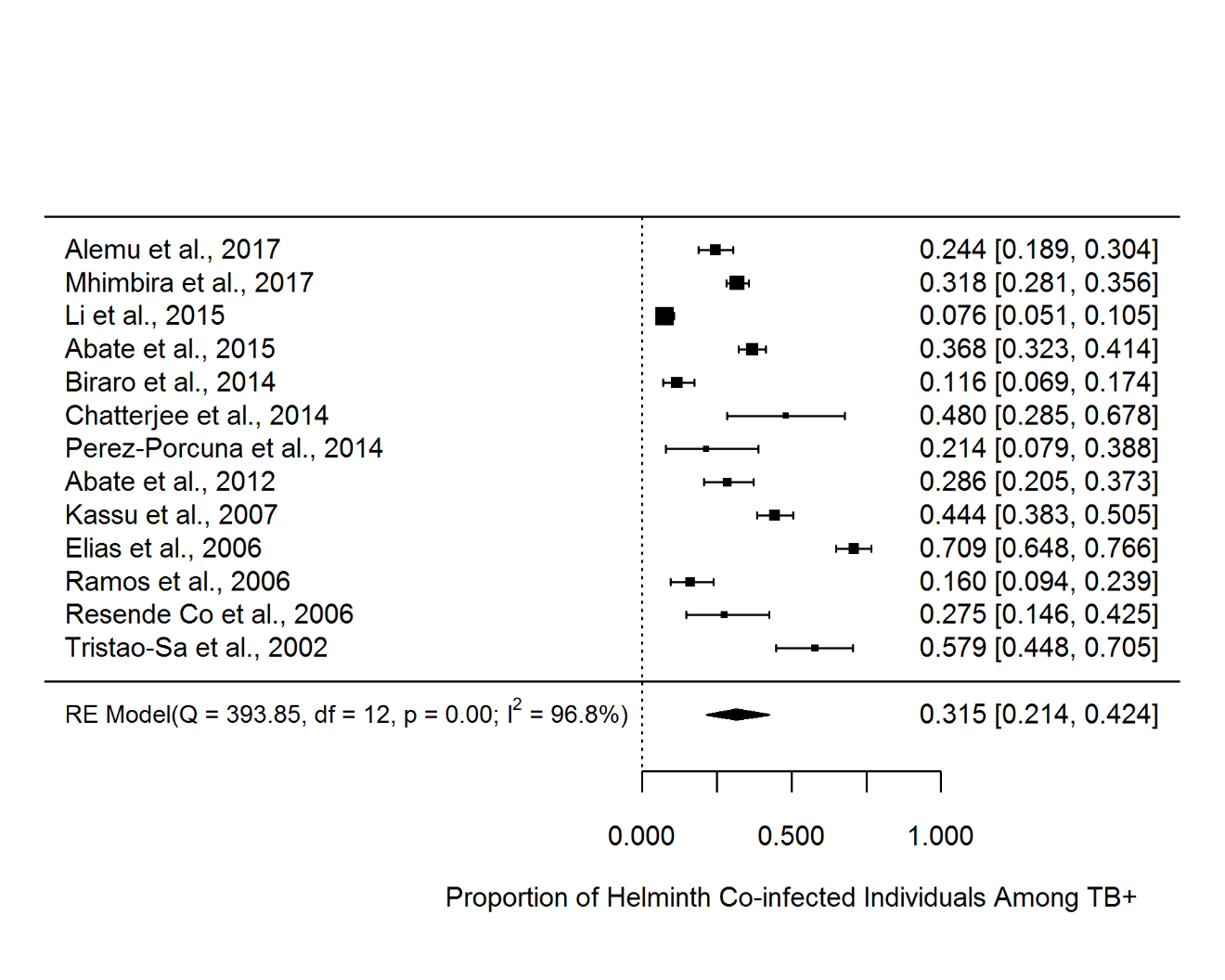

Supplement: S7 Fig — (TIF) [file pntd.0007455.s016.tif]

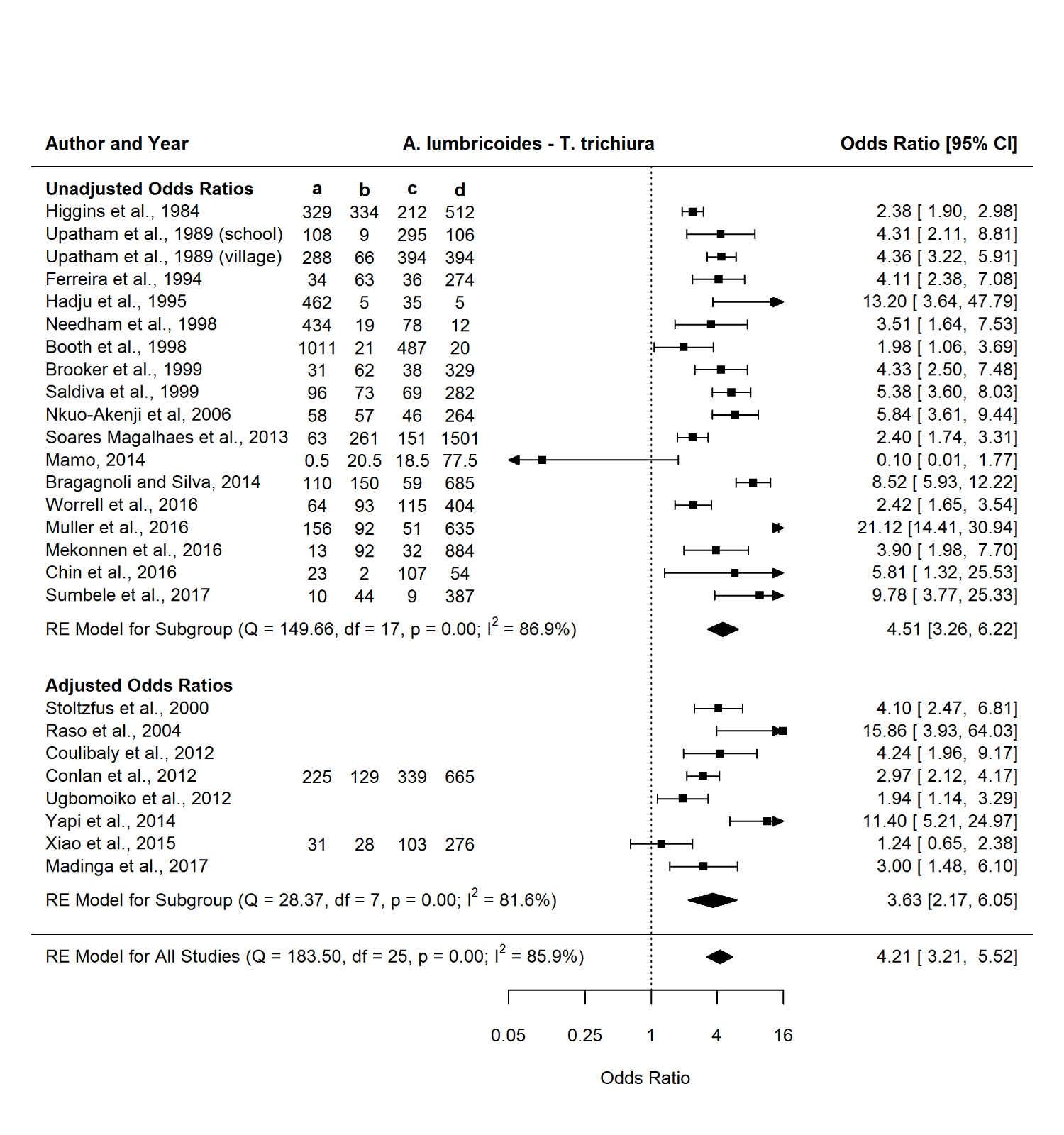

Supplement: S8 Fig — a = AL+/TT+; b = AL+/TT-; c = AL-/TT+; d = AL-/TT-; RE = random effects. Odds ratio compares the odds of A. lumbricoides infection among T. trichiura-positive individuals (a/c) compared to the odds of A lumbricoides infection among T. trichiura-negative individuals (b/d). (TIF) [file pntd.0007455.s017.tif]

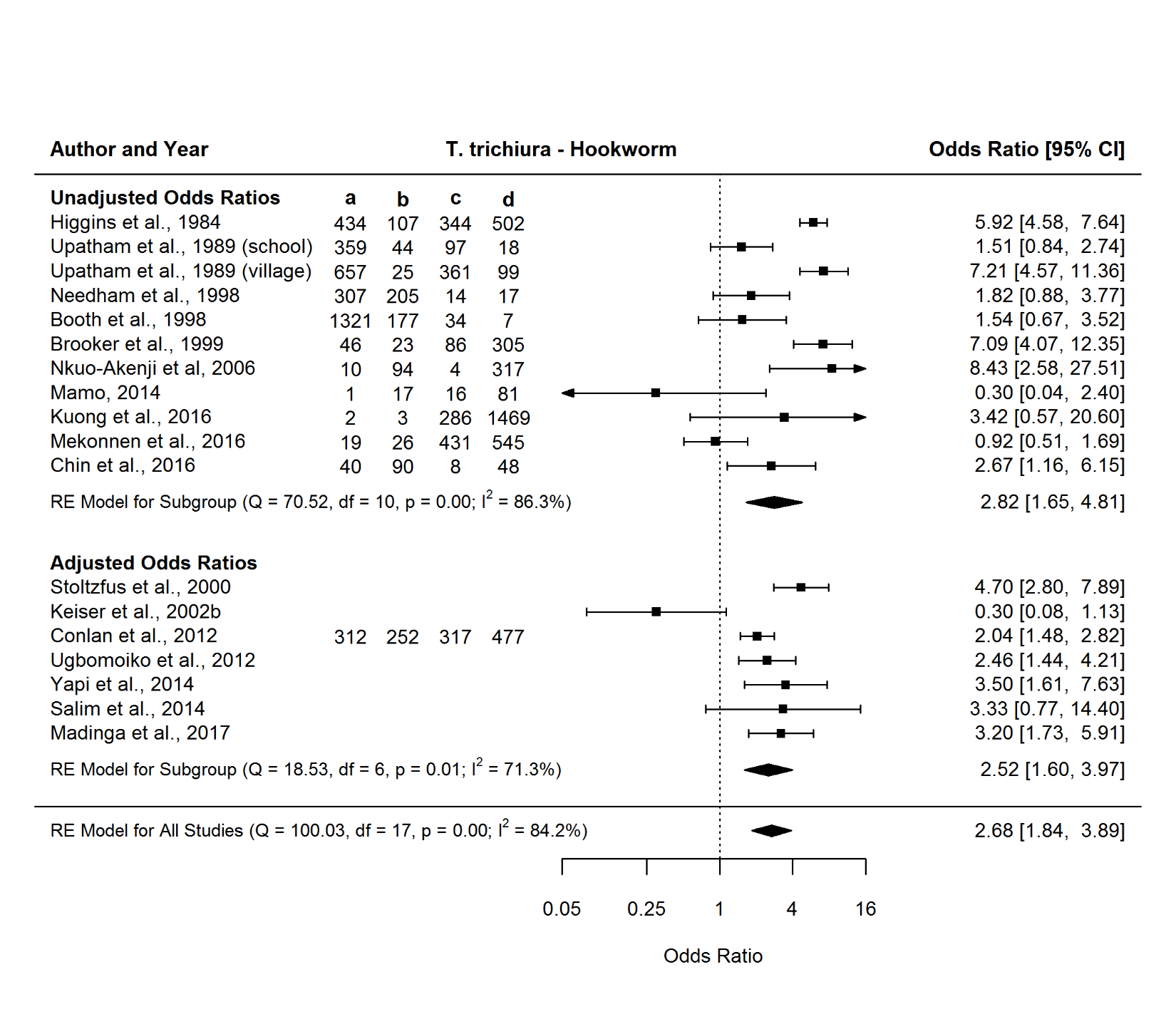

Supplement: S9 Fig — a = TT+/HW+; b = TT+/HW-; c = TT-/HW+; d = TT-/HW-; RE = random effects. Odds ratio compares the odds of T. trichiura infection among hookworm-positive individuals (a/c) compared to the odds of T. trichiura infection among hookworm-negative individuals (b/d). (TIF) [file pntd.0007455.s018.tif]

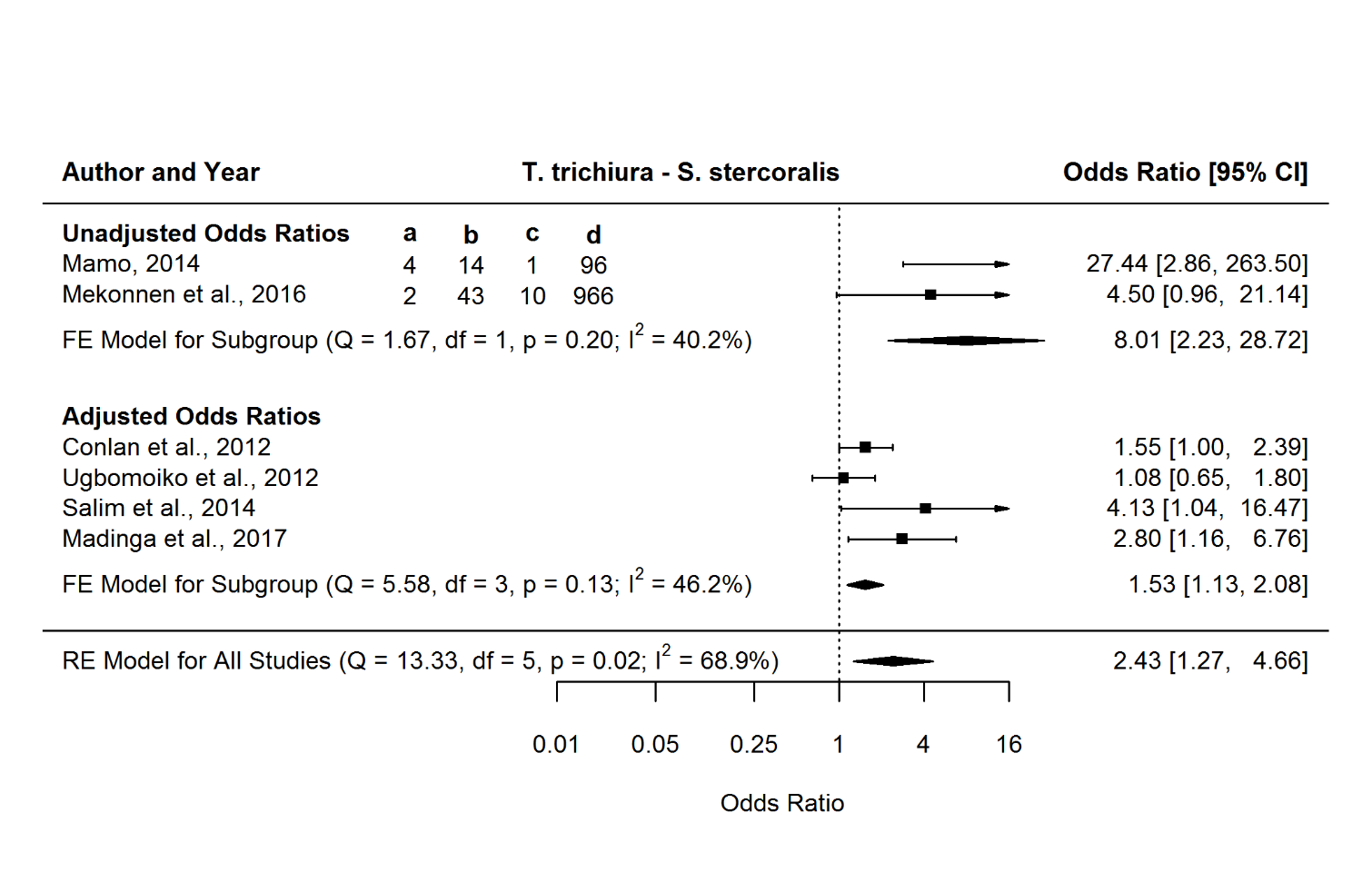

Supplement: S10 Fig — a = TT+/SS+; b = TT+/SS-; c = TT-/SS+; d = TT-/SS-; RE = random effects. Odds ratio compares the odds of T. trichiura infection among S. stercoralis-positive individuals (a/c) compared to the odds of T. trichiura infection among S. stercoralis-negative individuals (b/d). (TIF) [file pntd.0007455.s019.tif]

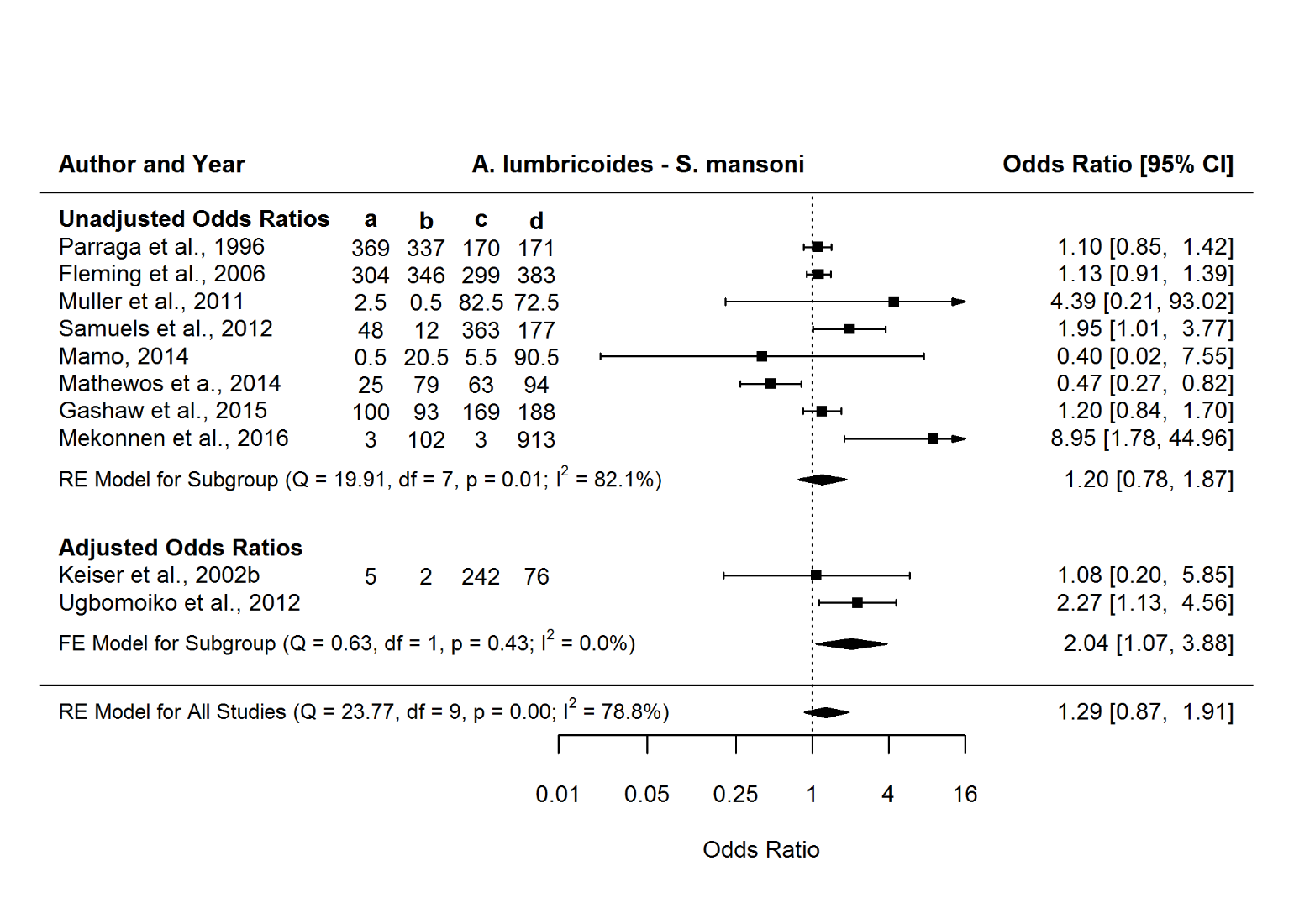

Supplement: S11 Fig — a = AL+/SM+; b = AL+/SM-; c = AL-/SM+; d = AL-/SM-; RE = random effects. Odds ratio compares the odds of A. lumbricoides infection among S. mansoni-positive individuals (a/c) compared to the odds of A lumbricoides infection among S. mansoni-negative individuals (b/d). (TIF) [file pntd.0007455.s020.tif]

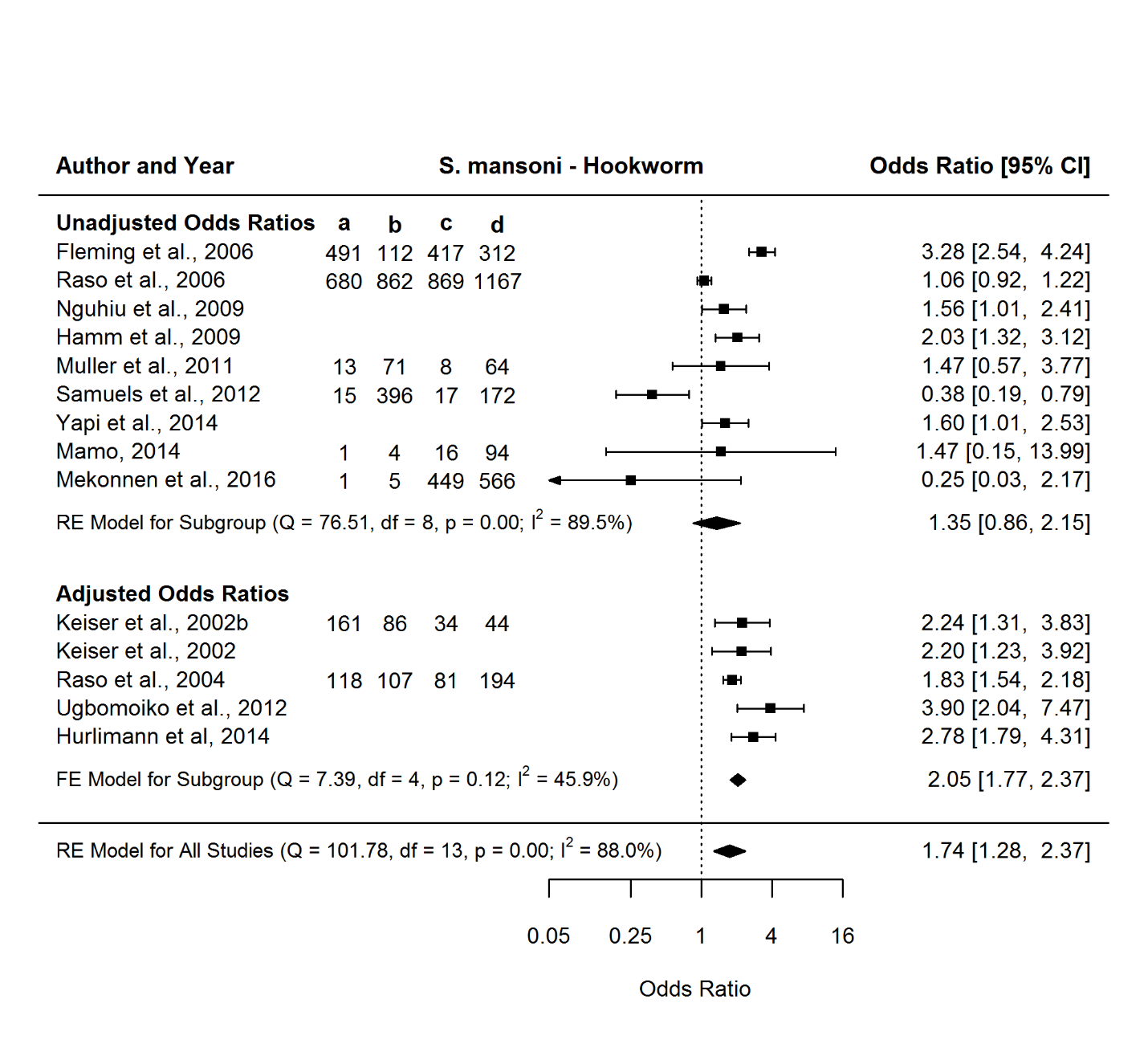

Supplement: S12 Fig — a = SM+/HW+; b = SM+/HW-; c = SM-/HW+; d = SM-/HW-; RE = random effects. Odds ratio compares the odds of S. mansoni infection among hookworm-positive individuals (a/c) compared to the odds of S. mansoni infection among hookworm-negative individuals (b/d). (TIF) [file pntd.0007455.s021.tif]

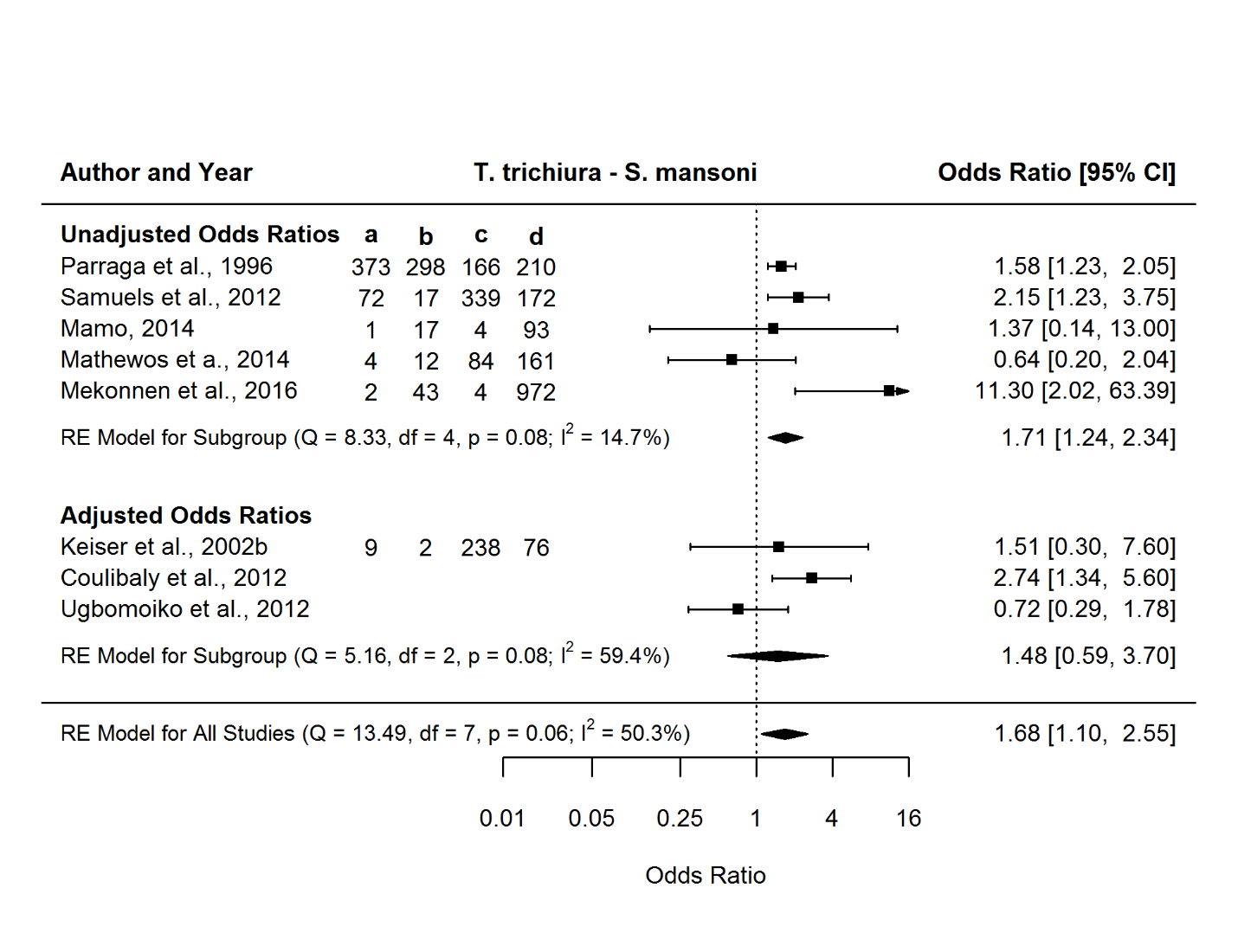

Supplement: S13 Fig — a = TT+/SM+; b = TT+/SM-; c = TT-/SM+; d = TT-/SM-; RE = random effects. Odds ratio compares the odds of T. trichiura infection among S. mansoni-positive individuals (a/c) compared to the odds of T. trichiura infection among S. mansoni-negative individuals (b/d). (TIF) [file pntd.0007455.s022.tif]

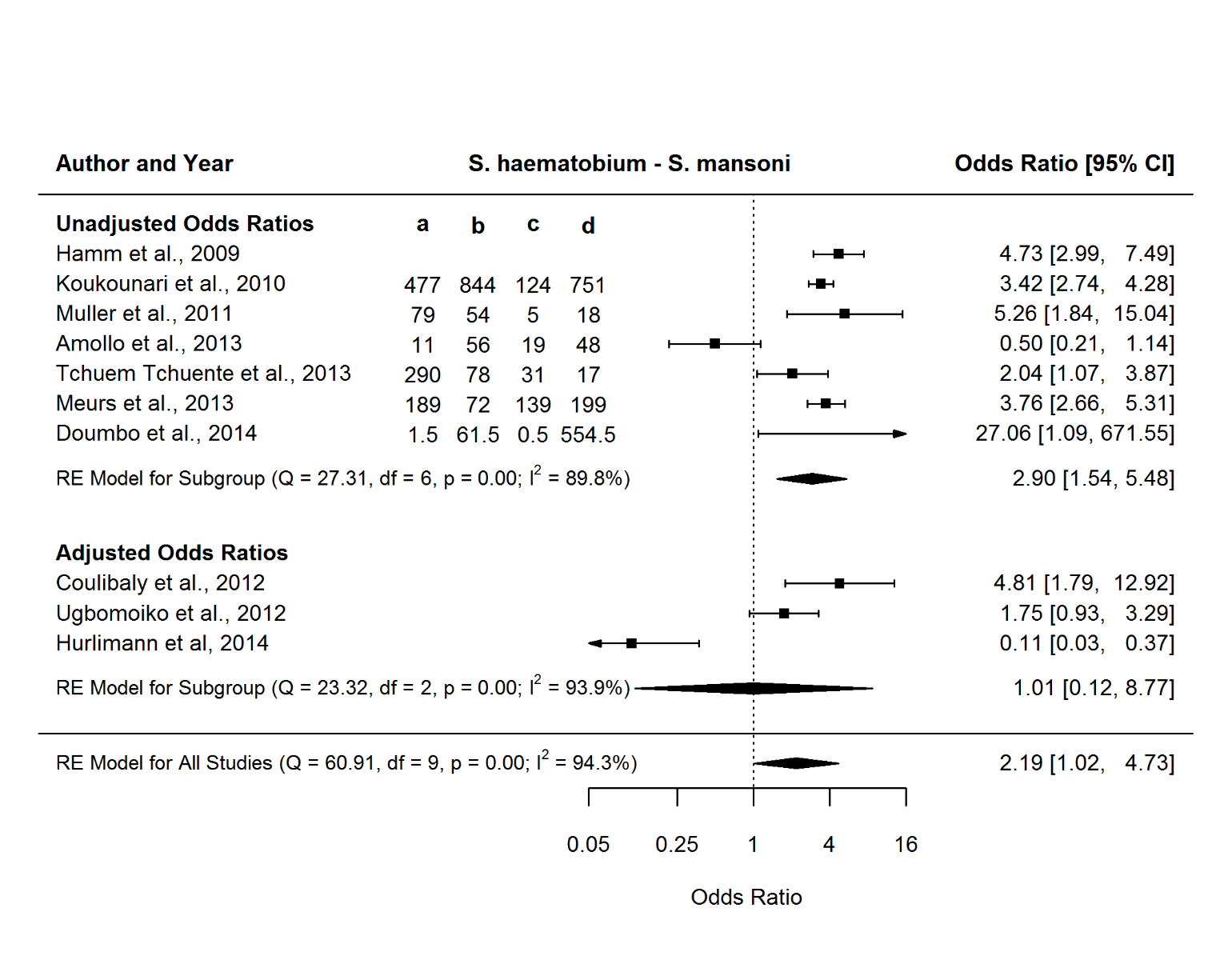

Supplement: S14 Fig — a = SH+/SM+; b = SH+/SM-; c = SH-/SM+; d = SH-/SM-; RE = random effects. Odds ratio compares the odds of S. haematobium infection among S. mansoni-positive individuals (a/c) compared to the odds of S. haematobium infection among S. mansoni-negative individuals (b/d). (TIF) [file pntd.0007455.s023.tif]

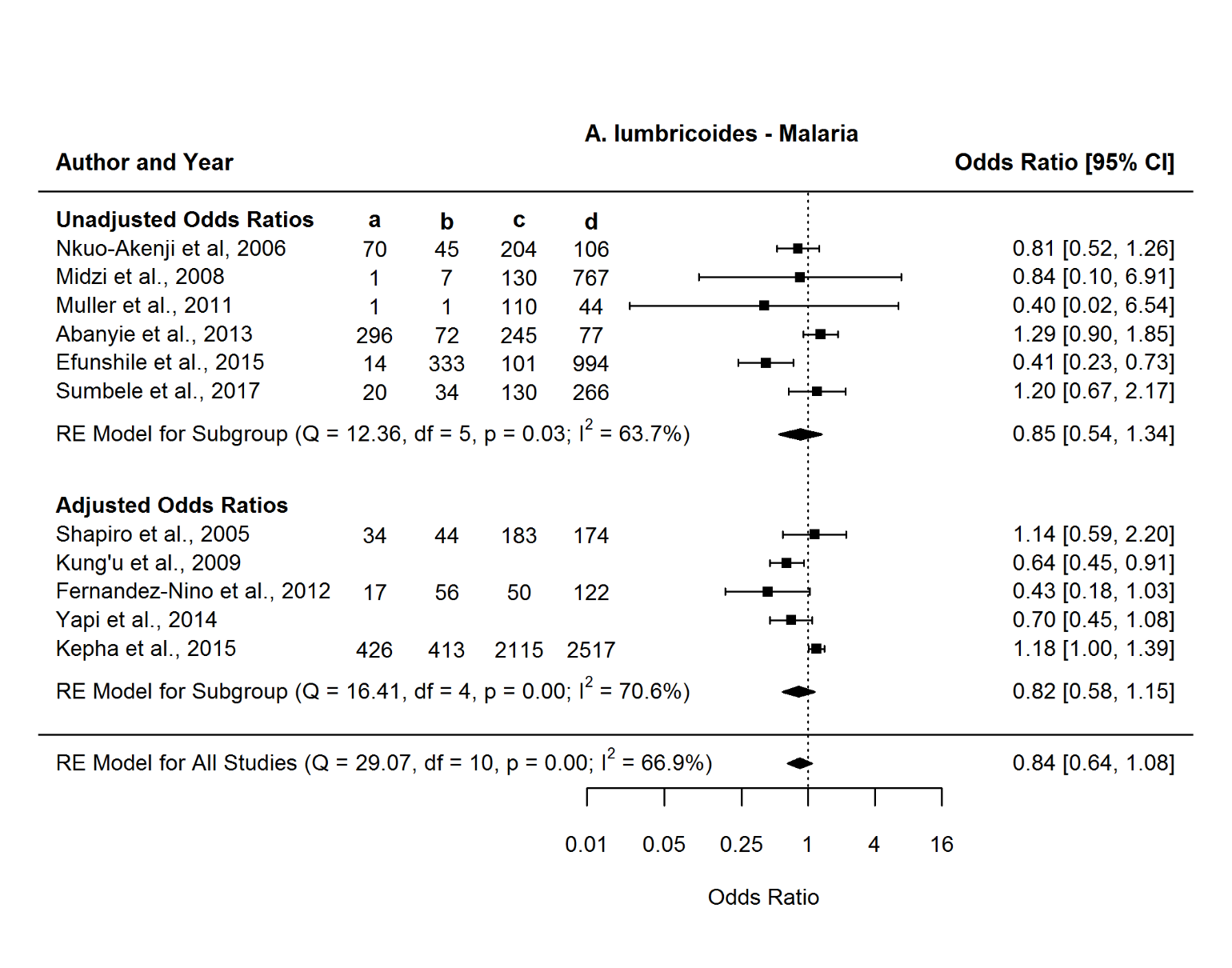

Supplement: S15 Fig — a = AL+/M+; b = AL+/M-; c = AL-/M+; d = AL-/M-; RE = random effects. Odds ratio compares the odds of A. lumbricoides infection among malaria-positive individuals (a/c) compared to the odds of A. lumbricoides infection among malaria-negative individuals (b/d). (TIF) [file pntd.0007455.s024.tif]

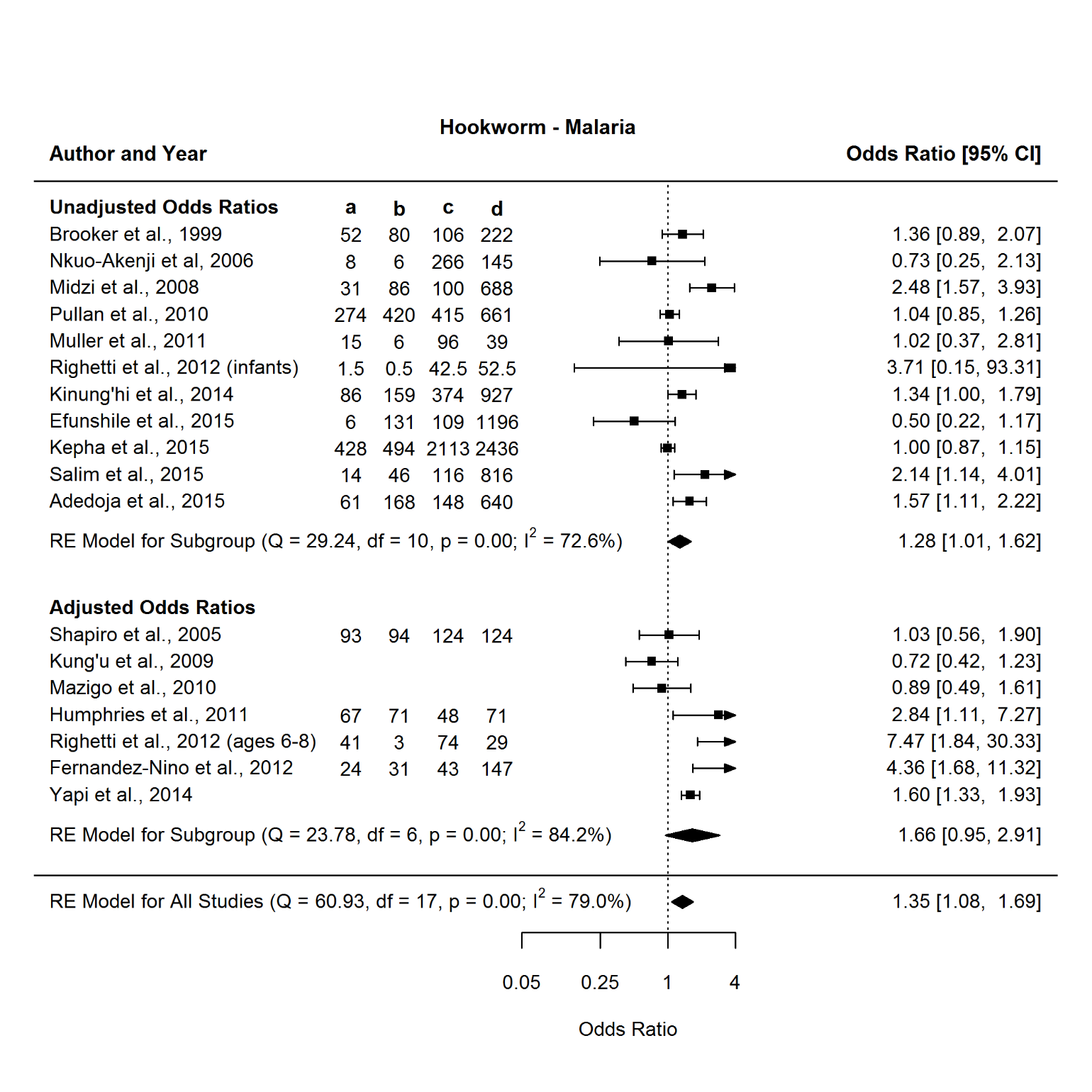

Supplement: S16 Fig — a = HW+/M+; b = HW+/M-; c = HW-/M+; d = HW-/M-; RE = random effects. Odds ratio compares the odds of hookworm infection among malaria-positive individuals (a/c) compared to the odds of hookworm infection among malaria-negative individuals (b/d). (TIF) [file pntd.0007455.s025.tif]

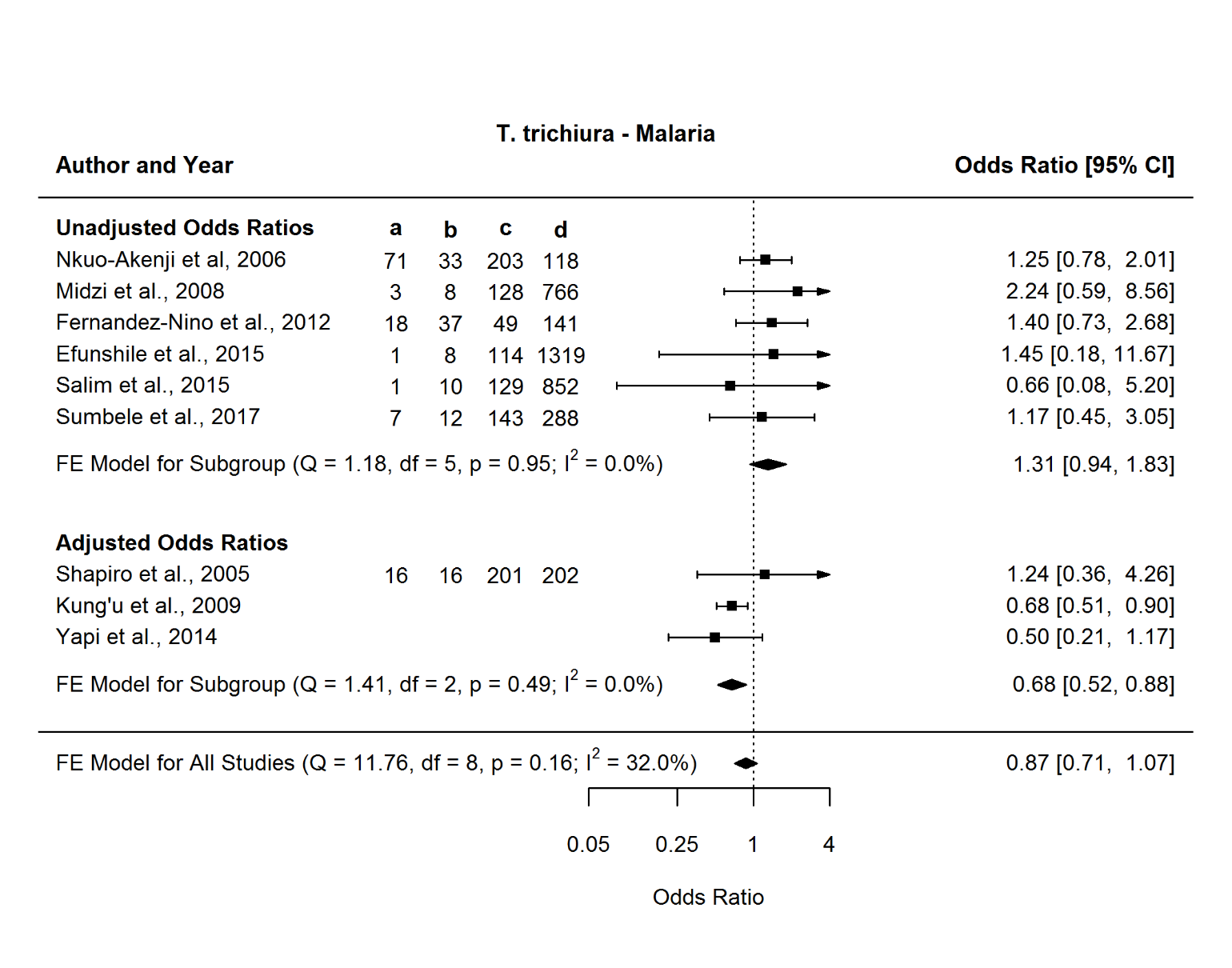

Supplement: S17 Fig — a = TT+/M+; b = TT+/M-; c = TT-/M+; d = TT-/M-; RE = random effects. Odds ratio compares the odds of T. trichiura infection among malaria-positive individuals (a/c) compared to the odds of T. trichiura infection among malaria-negative individuals (b/d). (TIF) [file pntd.0007455.s026.tif]

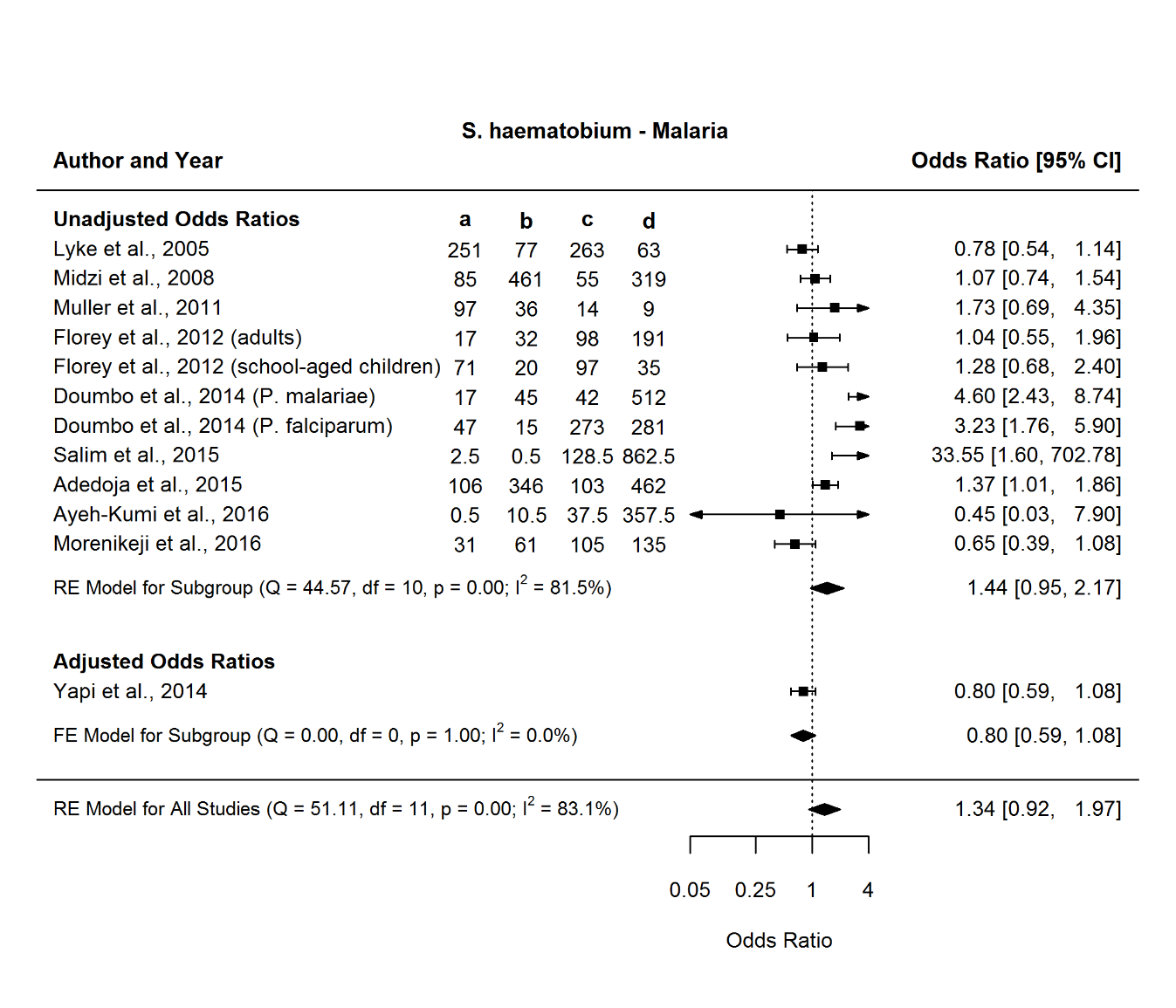

Supplement: S18 Fig — a = SH+/M+; b = SH+/M-; c = SH-/M+; d = SH-/M-; RE = random effects. Odds ratio compares the odds of S. haematobium infection among malaria-positive individuals (a/c) compared to the odds of S. haematobium infection among malaria-negative individuals (b/d). (TIF) [file pntd.0007455.s027.tif]

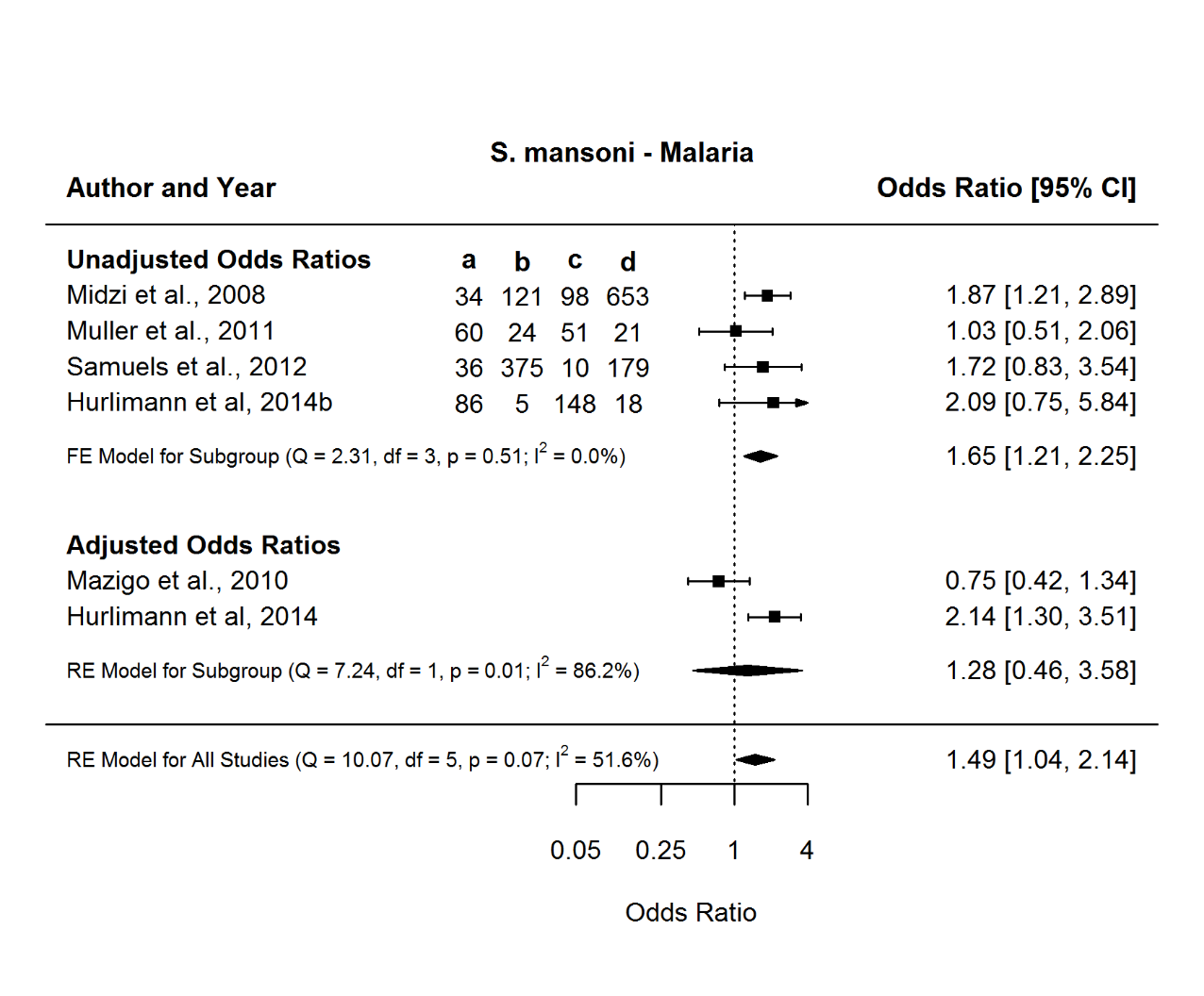

Supplement: S19 Fig — a = SM+/M+; b = SM+/M-; c = SM-/M+; d = SM-/M-; RE = random effects. Odds ratio compares the odds of S. mansoni infection among malaria-positive individuals (a/c) compared to the odds of S. mansoni infection among malaria-negative individuals (b/d). (TIF) [file pntd.0007455.s028.tif]
